# Supplementary material for: Testing the effectiveness of a mobile approach avoidance intervention and measuring approach biases in an ecological momentary assessment context: study protocol for a randomised-controlled trial
Source: BMJ Open. 2023 Apr 25;13(4):e070443. doi: 10.1136/bmjopen-2022-070443 (PMC10151942; doi:10.1136/bmjopen-2022-070443)
Supplement: Supplementary data [file bmjopen-2022-070443supp001.pdf]

**Supplementary material for the manuscript "Study protocol for a Randomized-Controlled Trial to Test the Effectiveness of a Mobile Approach Avoidance Intervention and to Measure Approach Biases in an EMA context"**

In this study, 219 valid German-speaking participants performed a single-session online approach-avoidance training where they were trained to avoid chocolate and approach fruit; their approach biases towards chocolate and fruit were measured before and after. Participants performed one of two variants of the AAT, both as a training and as an approach bias measurement. The irrelevant-feature AAT involved moving stimuli towards or away from oneself on the basis of the shape of a frame around them. The dual-feature AAT involved moving stimuli towards or away from oneself on the basis of the combination of stimulus content and frame type, such that one of the frames meant that foods were approached and objects avoided, while the other frame meant that foods were avoided and objects approached. Half of all participants in each training group received active training, and the other half received sham training. The pre- and post-training approach bias measurements each included 112 trials, of which half involved trained stimuli and half untrained stimuli, and in which all stimulus categories were approached and avoided equally often. The training involved 224 trials, of which 64 featured fruit stimuli, 64 featured chocolate stimuli, and 96 featured object stimuli. Control stimuli were always approached and avoided equally often. In the sham trainings, chocolate and fruit stimuli were approached and avoided equally often as well (32 times each). In the active training, chocolate stimuli were approached 56 times and avoided 8 times; vice versa for fruit stimuli. Our power analysis focused on the effect of the dual-feature AAT training on approach biases towards trained fruit stimuli. This training included 52 participants in the active training condition and 55 participants in the sham training condition.
