# Supplementary material for: Testing the effectiveness of a mobile approach avoidance intervention and measuring approach biases in an ecological momentary assessment context: study protocol for a randomised-controlled trial
Source: BMJ Open. 2023 Apr 25;13(4):e070443. doi: 10.1136/bmjopen-2022-070443 (PMC10151942; doi:10.1136/bmjopen-2022-070443)
Supplement: Supplementary data [file bmjopen-2022-070443supp002.pdf]

Supplementary Table 1: Statistical power for a range of effect sizes and sample sizes.

Sample size	*Effect size (Hedges' g)*										
	.28	*.36*	*.43*	*.5*	*.57*	*.65*	*.72*	*.79*	*.86*	*.93*	*1.01*
80	.19	.28	.38	.52	.64	.72	.82	.90	.94	.98	.98
90	.24	.34	.43	.58	.70	.76	.89	.94	.96	.96	.99
100	.17	.32	.46	.65	.69	.83	.86	.94	.98	.98	1
110	.24	.42	.57	.66	.82	.85	.92	.97	.99	1	1
120	.23	.41	.62	.66	.81	.89	.94	.97	1	1	.99
130	.34	.36	.62	.78	.86	.90	.97	.99	.99	1	.99
140	.32	.49	.56	.77	.88	.97	.97	.99	1	.99	1
150	.36	.52	.63	.78	.88	.94	.97	1	1	1	1
160	.35	.57	.68	.82	.90	.96	.98	.99	.99	1	1
170	.41	.54	.72	.83	.92	.97	.98	.99	1	1	1
180	.37	.54	.70	.90	.92	.98	.99	.99	1	1	1
