# Supplementary material for: Testing the effectiveness of a mobile approach avoidance intervention and measuring approach biases in an ecological momentary assessment context: study protocol for a randomised-controlled trial
Source: BMJ Open. 2023 Apr 25;13(4):e070443. doi: 10.1136/bmjopen-2022-070443 (PMC10151942; doi:10.1136/bmjopen-2022-070443)
Supplement: Supplementary data [file bmjopen-2022-070443supp003.pdf]

BMJ Publishing Group Limited (BMJ) disclaims all liability and responsibility arising from any reliance placed on this supplemental material which has been supplied by the author(s)

Teil A: Zahlencode

A1. **Bitte geben Sie hier Ihren Zahlencode aus der Mail ein:**

Teil B: Demografische Variablen

Bitte beantworten Sie zunächst einige Fragen zu Ihrer Person.

Leider keine Teilnahme möglich

Eine der Apps, die für die Studie benötigt wird, funktioniert momentan nur mit Android-Betriebssystemen. Deshalb können Sie leider ohne ein entsprechendes Smartphone nicht an der Studie teilnehmen. Falls Sie an einer anderen psychologischen Studie teilnehmen wollen, finden Sie eine Studienliste unserer Abteilung auf dieser Seite: https://sites.google.com/site/eatingandanxietylab/mitmachen

Sie können diesen Fragebogen jetzt schließen.

Leider keine Teilnahme möglich

Leider erfüllen Sie nicht die Einschlusskriterien für diese Studie. Falls Sie an einer anderen psychologischen Studie teilnehmen wollen, finden Sie eine Studienliste unserer Abteilung auf dieser Seite: https://sites.google.com/site/eatingandanxietylab/mitmachen

Sie können diesen Fragebogen jetzt schließen.

Leider keine Teilnahme möglich

Leider erfüllen Sie nicht die Einschlusskriterien für diese Studie. Falls Sie an einer anderen psychologischen Studie teilnehmen wollen, finden Sie eine Studienliste unserer Abteilung auf dieser Seite: https://sites.google.com/site/eatingandanxietylab/mitmachen

Sie können diesen Fragebogen jetzt schließen.

B1. Um an der Studie teilnehmen zu können, benötigen Sie ein Smartphone mit einem Android-Betriebssystem. Bitte geben Sie daher hier die auf Sie passende Antwort an:

- ☐ Ich habe ein Smartphone mit Android-Betriebssystem zur Verfügung (ein eigenes, oder für die Dauer der Studie aus meinem Umfeld organisiert).
- ☐ Ich habe kein Smartphone mit Android-Betriebssystem, wäre aber bereit, mir für die Studie eines von der Uni Salzburg auszuleihen.
- ☐ Ich habe kein Smartphone mit Android-Betriebssystem zur Verfügung und wäre nicht bereit, mir für die Studie eines von der Uni Salzburg auszuleihen.

B2. Bitte geben Sie Ihr Alter an.

☐☐☐☐☐☐☐☐☐☐

B3. Bitte geben Sie Ihr Geschlecht an.

- weiblich ☐
- männlich ☐
- divers ☐

B4. Sind Sie schwanger?

- Ja ☐
- Nein ☐

B5. Bitte wählen Sie alle auf Sie zutreffenden Optionen bezüglich Ihres Menstruationszyklus und/oder das von Ihnen aktuell verwendete Verhütungsmittel aus.

- Trifft nicht zu ☐
- Natürlicher Menstruationszyklus ☐
- Verwendung der Kupferspirale, Kupferkette, Goldspirale ☐
- Verwendung der Hormonspirale ☐
- Verwendung der Pille ☐
- Verwendung der Pille, die IMMER eingenommen wird ☐
- Dreimonatsspritze ☐
- Hormonimplantat ☐
- Verhütungspflaster ☐
- Hormonersatztherapie ☐
- Menopause ☐

BMJ Publishing Group Limited (BMJ) disclaims all liability and responsibility arising from any reliance placed on this supplemental material which has been supplied by the author(s)

Sonstiges

Sonstiges

B6. **Bitte geben Sie den Starttag Ihrer letzten Menstruation an.**

B7. **Bitte geben Sie den Starttag Ihrer vorletzten Menstruation an.**

B8. **Bitte geben Sie den Starttag Ihrer drittletzten Menstruation an.**

BMJ Publishing Group Limited (BMJ) disclaims all liability and responsibility arising from any reliance placed on this supplemental material which has been supplied by the author(s)

B9. Bitte tragen Sie Ihre Nationalität ein.

B10. Was ist Ihr höchster erreichter Bildungsabschluss?

- Ohne Schulabschluss ☐
- Hauptschulabschluss ☐
- abgeschlossene Lehre ☐
- mittlere Reife ☐
- Abitur / Matura ☐
- abgeschlossenes Bachelorstudium ☐
- abgeschlossenes Masterstudium ☐
- Sonstiges ☐

Sonstiges

B11. Welcher Berufsgruppe gehören Sie an?

- Student/in ☐
- Auszubildende/r ☐
- Schüler/in ☐
- Angestellte/r ☐
- Selbstständige/r ☐
- Sonstiges ☐

Sonstiges

B12. Wie ernähren Sie sich?

- Vegan (Ernährung ohne von Tieren stammenden Nahrungsmittel) ☐
- Vegetarisch (fleisch- und fischfreie Ernährung) ☐
- Pescetarisch (fleisch- aber nicht fischfreie Ernährung) ☐
- Omnivor (kein Nahrungsmittelverzicht) ☐
- Sonstiges ☐

Sonstiges

B13. Bitte geben Sie Ihre aktuelle Körpergröße an.

Angabe in Zentimetern.

B14. Bitte geben Sie Ihr aktuelles Gewicht an.

Angabe in Kilogramm.

B15. Haben Sie eine Nahrungsmittelunverträglichkeit?

Ja ☐

Nein ☐

B16. Welche Nahrungsmittelunverträglichkeit haben Sie?

B17. Haben Sie aktuell (d.h. innerhalb der letzten 12 Wochen) eine diagnostizierte Essstörung?

Ja ☐

Nein ☐

Teil C: Konsumziele 1/9

Auf den nächsten 9 Seiten bekommen Sie Bilder von insgesamt 90 Nahrungsmitteln angezeigt und die Aufforderung, jeweils Ihren vergangenen und zukünftigen Konsum der abgebildeten Nahrungsmittel anzugeben. Die Anpassung der Studie auf Ihre Ernährungsziele erfolgt aufgrund Ihrer Antworten in diesem Teil des Fragebogens. Für eine möglichst gute Individualisierung ist es daher wichtig, dass Sie die Fragen möglichst genau beantworten.

C1. Erdnussflips

An wie vielen Tagen haben Sie in den letzten drei Wochen Erdnussflips gegessen?|an keinem Tag|an allen 21 Tagen

An wie vielen Tagen wollen Sie in den nächsten drei Wochen Erdnussflips essen?|an keinem Tag|an allen 21 Tagen

C2. gemischtes Müsli

An wie vielen Tagen haben Sie in den letzten drei Wochen gemischtes Müsli gegessen?|an keinem Tag|an allen 21 Tagen

An wie vielen Tagen wollen Sie in den nächsten drei Wochen gemischtes Müsli essen?|an keinem Tag|an allen 21 Tagen

C3. Nussmischung

An wie vielen Tagen haben Sie in den letzten drei Wochen Studentenfutter gegessen?|an keinem Tag|an allen 21 Tagen

An wie vielen Tagen wollen Sie in den nächsten drei Wochen Studentenfutter essen?|an keinem Tag|an allen 21 Tagen

C4. Fisch

An wie vielen Tagen haben Sie in den letzten drei Wochen Fisch gegessen?|an keinem Tag|an allen 21 Tagen

An wie vielen Tagen wollen Sie in den nächsten drei Wochen Fisch essen?|an keinem Tag|an allen 21 Tagen

C5. salziger Snackmix

An wie vielen Tagen haben Sie in den letzten drei Wochen salzigen Snackmix gegessen?|an keinem Tag|an allen 21 Tagen

An wie vielen Tagen wollen Sie in den nächsten drei Wochen salzigen Snackmix essen?|an keinem Tag|an allen 21 Tagen

C6. Pizza

An wie vielen Tagen haben Sie in den letzten drei Wochen Pizza gegessen?|an keinem Tag|an allen 21 Tagen

An wie vielen Tagen wollen Sie in den nächsten drei Wochen Pizza essen?|an keinem Tag|an allen 21 Tagen

C7. Honig

An wie vielen Tagen haben Sie in den letzten drei Wochen Honig gegessen?|an keinem Tag|an allen 21 Tagen

An wie vielen Tagen wollen Sie in den nächsten drei Wochen Honig essen?|an keinem Tag|an allen 21 Tagen

C8. Chicken Nuggets

An wie vielen Tagen haben Sie in den letzten drei Wochen Chicken Nuggets gegessen?|an keinem Tag|an allen 21 Tagen

An wie vielen Tagen wollen Sie in den nächsten drei Wochen Chicken Nuggets essen?|an keinem Tag|an allen 21 Tagen

C9. Tofu

An wie vielen Tagen haben Sie in den letzten drei Wochen Tofu gegessen?|an keinem Tag|an allen 21 Tagen

An wie vielen Tagen wollen Sie in den nächsten drei Wochen Tofu essen?|an keinem Tag|an allen 21 Tagen

C10. Paprika

An wie vielen Tagen haben Sie in den letzten drei Wochen Paprika gegessen?|an keinem Tag|an allen 21 Tagen

An wie vielen Tagen wollen Sie in den nächsten drei Wochen Paprika essen?|an keinem Tag|an allen 21 Tagen

Teil D: Konsumziele 2/9

D1. Tee

An wie vielen Tagen haben Sie in den letzten drei Wochen Tee getrunken?|an keinem Tag|an allen 21 Tagen

An wie vielen Tagen wollen Sie in den nächsten drei Wochen Tee trinken?|an keinem Tag|an allen 21 Tagen

D2. Fischstäbchen

An wie vielen Tagen haben Sie in den letzten drei Wochen Fischstäbchen gegessen?|an keinem Tag|an allen 21 Tagen

An wie vielen Tagen wollen Sie in den nächsten drei Wochen Fischstäbchen essen?|an keinem Tag|an allen 21 Tagen

D3. Pilze

An wie vielen Tagen haben Sie in den letzten drei Wochen Pilze gegessen?|an keinem Tag|an allen 21 Tagen

An wie vielen Tagen wollen Sie in den nächsten drei Wochen Pilze essen?|an keinem Tag|an allen 21 Tagen

D4. salziges Gebäck

An wie vielen Tagen haben Sie in den letzten drei Wochen salziges Gebäck gegessen?|an keinem Tag|an allen 21 Tagen

An wie vielen Tagen wollen Sie in den nächsten drei Wochen salziges Gebäck essen?|an keinem Tag|an allen 21 Tagen

D5. Gurke

An wie vielen Tagen haben Sie in den letzten drei Wochen Gurke gegessen?|an keinem Tag|an allen 21 Tagen

An wie vielen Tagen wollen Sie in den nächsten drei Wochen Gurke essen?|an keinem Tag|an allen 21 Tagen

D6. Quinoa

An wie vielen Tagen haben Sie in den letzten drei Wochen Quinoa gegessen?|an keinem Tag|an allen 21 Tagen

An wie vielen Tagen wollen Sie in den nächsten drei Wochen Quinoa essen?|an keinem Tag|an allen 21 Tagen

D7. Kartoffelbrei

An wie vielen Tagen haben Sie in den letzten drei Wochen Kartoffelbrei gegessen?|an keinem Tag|an allen 21 Tagen

An wie vielen Tagen wollen Sie in den nächsten drei Wochen Kartoffelbrei essen?|an keinem Tag|an allen 21 Tagen

D8. Schokonüsse

An wie vielen Tagen haben Sie in den letzten drei Wochen Schokonüsse gegessen?|an keinem Tag|an allen 21 Tagen

An wie vielen Tagen wollen Sie in den nächsten drei Wochen Schokonüsse essen?|an keinem Tag|an allen 21 Tagen

D9. Hummus

An wie vielen Tagen haben Sie in den letzten drei Wochen Hummus gegessen?|an keinem Tag|an allen 21 Tagen

An wie vielen Tagen wollen Sie in den nächsten drei Wochen Hummus essen?|an keinem Tag|an allen 21 Tagen

D10. Kiwi

An wie vielen Tagen haben Sie in den letzten drei Wochen Kiwi gegessen?|an keinem Tag|an allen 21 Tagen

An wie vielen Tagen wollen Sie in den nächsten drei Wochen Kiwi essen?|an keinem Tag|an allen 21 Tagen

Teil E: Konsumziele 3/9

E1. Lasagne

An wie vielen Tagen haben Sie in den letzten drei Wochen Lasagne gegessen?|an keinem Tag|an allen 21 Tagen

An wie vielen Tagen wollen Sie in den nächsten drei Wochen Lasagne essen?|an keinem Tag|an allen 21 Tagen

E2. Nüsse im Teigmantel

An wie vielen Tagen haben Sie in den letzten drei Wochen Nüsse im Teigmantel gegessen?|an keinem Tag|an allen 21 Tagen

An wie vielen Tagen wollen Sie in den nächsten drei Wochen Nüsse im Teigmantel essen?|an keinem Tag|an allen 21 Tagen

E3. Zucchini

An wie vielen Tagen haben Sie in den letzten drei Wochen Zucchini gegessen?|an keinem Tag|an allen 21 Tagen

An wie vielen Tagen wollen Sie in den nächsten drei Wochen Zucchini essen?|an keinem Tag|an allen 21 Tagen

E4. Wassereis

An wie vielen Tagen haben Sie in den letzten drei Wochen Wassereis gegessen?|an keinem Tag|an allen 21 Tagen

An wie vielen Tagen wollen Sie in den nächsten drei Wochen Wassereis essen?|an keinem Tag|an allen 21 Tagen

E5. Linsen

An wie vielen Tagen haben Sie in den letzten drei Wochen Linsen gegessen?|an keinem Tag|an allen 21 Tagen

An wie vielen Tagen wollen Sie in den nächsten drei Wochen Linsen essen?|an keinem Tag|an allen 21 Tagen

E6. Avocado

An wie vielen Tagen haben Sie in den letzten drei Wochen Avocado gegessen?|an keinem Tag|an allen 21 Tagen

An wie vielen Tagen wollen Sie in den nächsten drei Wochen Avocado essen?|an keinem Tag|an allen 21 Tagen

E7. Möhren

An wie vielen Tagen haben Sie in den letzten drei Wochen Möhren gegessen?|an keinem Tag|an allen 21 Tagen

An wie vielen Tagen wollen Sie in den nächsten drei Wochen Möhren essen?|an keinem Tag|an allen 21 Tagen

E8. Cappuccino

An wie vielen Tagen haben Sie in den letzten drei Wochen Cappuccino getrunken?|an keinem Tag|an allen 21 Tagen

An wie vielen Tagen wollen Sie in den nächsten drei Wochen Cappuccino trinken?|an keinem Tag|an allen 21 Tagen

E9. Salzcracker

An wie vielen Tagen haben Sie in den letzten drei Wochen Cracker gegessen?|an keinem Tag|an allen 21 Tagen

An wie vielen Tagen wollen Sie in den nächsten drei Wochen Cracker essen?|an keinem Tag|an allen 21 Tagen

E10. Milch

An wie vielen Tagen haben Sie in den letzten drei Wochen Milch getrunken?|an keinem Tag|an allen 21 Tagen

An wie vielen Tagen wollen Sie in den nächsten drei Wochen Milch trinken?|an keinem Tag|an allen 21 Tagen

Teil F: Konsumziele 4/9

F1. Karamellriegel

An wie vielen Tagen haben Sie in den letzten drei Wochen Karamellriegel gegessen?|an keinem Tag|an allen 21 Tagen

An wie vielen Tagen wollen Sie in den nächsten drei Wochen Karamellriegel essen?|an keinem Tag|an allen 21 Tagen

F2. Marmelade

An wie vielen Tagen haben Sie in den letzten drei Wochen Marmelade gegessen?|an keinem Tag|an allen 21 Tagen

An wie vielen Tagen wollen Sie in den nächsten drei Wochen Marmelade essen?|an keinem Tag|an allen 21 Tagen

F3. Frühlingsrollen

An wie vielen Tagen haben Sie in den letzten drei Wochen Frühlingsrollen gegessen?|an keinem Tag|an allen 21 Tagen

An wie vielen Tagen wollen Sie in den nächsten drei Wochen Frühlingsrollen essen?|an keinem Tag|an allen 21 Tagen

F4. Butter

An wie vielen Tagen haben Sie in den letzten drei Wochen Butter gegessen?|an keinem Tag|an allen 21 Tagen

An wie vielen Tagen wollen Sie in den nächsten drei Wochen Butter essen?|an keinem Tag|an allen 21 Tagen

F5. Mandarine/ Orange

An wie vielen Tagen haben Sie in den letzten drei Wochen Mandarine/ Orange gegessen?|an keinem Tag|an allen 21 Tagen

An wie vielen Tagen wollen Sie in den nächsten drei Wochen Mandarine/ Orange essen?|an keinem Tag|an allen 21 Tagen

F6. Müsliriegel

An wie vielen Tagen haben Sie in den letzten drei Wochen Müsliriegel gegessen?|an keinem Tag|an allen 21 Tagen

An wie vielen Tagen wollen Sie in den nächsten drei Wochen Müsliriegel essen?|an keinem Tag|an allen 21 Tagen

F7. Weingummis

An wie vielen Tagen haben Sie in den letzten drei Wochen Weingummis gegessen?|an keinem Tag|an allen 21 Tagen

An wie vielen Tagen wollen Sie in den nächsten drei Wochen Weingummis essen?|an keinem Tag|an allen 21 Tagen

F8. Schnitzel

An wie vielen Tagen haben Sie in den letzten drei Wochen Schnitzel gegessen?|an keinem Tag|an allen 21 Tagen

An wie vielen Tagen wollen Sie in den nächsten drei Wochen Schnitzel essen?|an keinem Tag|an allen 21 Tagen

F9. Chips

An wie vielen Tagen haben Sie in den letzten drei Wochen Chips gegessen?|an keinem Tag|an allen 21 Tagen

An wie vielen Tagen wollen Sie in den nächsten drei Wochen Chips essen?|an keinem Tag|an allen 21 Tagen

F10. Gemüsesuppe

An wie vielen Tagen haben Sie in den letzten drei Wochen Gemüsesuppe gegessen?|an keinem Tag|an allen 21 Tagen

An wie vielen Tagen wollen Sie in den nächsten drei Wochen Gemüsesuppe essen?|an keinem Tag|an allen 21 Tagen

Teil G: Konsumziele 5/9

G1. Ei

An wie vielen Tagen haben Sie in den letzten drei Wochen Eier (in jeglicher Zubereitungsform) gegessen?|an keinem Tag|an allen 21 Tagen

An wie vielen Tagen wollen Sie in den nächsten drei Wochen Eier (in jeglicher Zubereitungsform) essen?|an keinem Tag|an allen 21 Tagen

G2. Tortellini

An wie vielen Tagen haben Sie in den letzten drei Wochen Tortellini gegessen?|an keinem Tag|an allen 21 Tagen

An wie vielen Tagen wollen Sie in den nächsten drei Wochen Tortellini essen?|an keinem Tag|an allen 21 Tagen

G3. Bratwurst

An wie vielen Tagen haben Sie in den letzten drei Wochen Bratwurst gegessen?|an keinem Tag|an allen 21 Tagen

An wie vielen Tagen wollen Sie in den nächsten drei Wochen Bratwurst essen?|an keinem Tag|an allen 21 Tagen

G4. Schokoladencornflakes

An wie vielen Tagen haben Sie in den letzten drei Wochen Schokoladencornflakes gegessen?|an keinem Tag|an allen 21 Tagen

An wie vielen Tagen wollen Sie in den nächsten drei Wochen Schokoladencornflakes essen?|an keinem Tag|an allen 21 Tagen

G5. Hähnchenfleisch

An wie vielen Tagen haben Sie in den letzten drei Wochen Hähnchenfleisch gegessen?|an keinem Tag|an allen 21 Tagen

An wie vielen Tagen wollen Sie in den nächsten drei Wochen Hähnchenfleisch essen?|an keinem Tag|an allen 21 Tagen

G6. Softdrinks

An wie vielen Tagen haben Sie in den letzten drei Wochen Softdrinks getrunken?|an keinem Tag|an allen 21 Tagen

An wie vielen Tagen wollen Sie in den nächsten drei Wochen Softdrinks trinken?|an keinem Tag|an allen 21 Tagen

G7. Popcorn

An wie vielen Tagen haben Sie in den letzten drei Wochen Popcorn gegessen?|an keinem Tag|an allen 21 Tagen

An wie vielen Tagen wollen Sie in den nächsten drei Wochen Popcorn essen?|an keinem Tag|an allen 21 Tagen

G8. Pfannkuchen/ Palatschinken

An wie vielen Tagen haben Sie in den letzten drei Wochen Pfannkuchen/ Palatschinken gegessen?|an keinem Tag|an allen 21 Tagen

An wie vielen Tagen wollen Sie in den nächsten drei Wochen Pfannkuchen/ Palatschinken essen?|an keinem Tag|an allen 21 Tagen

G9. Banane

An wie vielen Tagen haben Sie in den letzten drei Wochen Banane gegessen?|an keinem Tag|an allen 21 Tagen

An wie vielen Tagen wollen Sie in den nächsten drei Wochen Banane essen?|an keinem Tag|an allen 21 Tagen

G10. Käse

An wie vielen Tagen haben Sie in den letzten drei Wochen Käse gegessen?|an keinem Tag|an allen 21 Tagen

An wie vielen Tagen wollen Sie in den nächsten drei Wochen Käse essen?|an keinem Tag|an allen 21 Tagen

Teil H: Konsumziele 6/9

H1. Knödel

An wie vielen Tagen haben Sie in den letzten drei Wochen Knödel gegessen?|an keinem Tag|an allen 21 Tagen

An wie vielen Tagen wollen Sie in den nächsten drei Wochen Knödel essen?|an keinem Tag|an allen 21 Tagen

H2. Pralinen

An wie vielen Tagen haben Sie in den letzten drei Wochen Pralinen gegessen?|an keinem Tag|an allen 21 Tagen

An wie vielen Tagen wollen Sie in den nächsten drei Wochen Pralinen essen?|an keinem Tag|an allen 21 Tagen

H3. Burger

An wie vielen Tagen haben Sie in den letzten drei Wochen Burger gegessen?|an keinem Tag|an allen 21 Tagen

An wie vielen Tagen wollen Sie in den nächsten drei Wochen Burger essen?|an keinem Tag|an allen 21 Tagen

H4. Weintrauben

An wie vielen Tagen haben Sie in den letzten drei Wochen Weintrauben gegessen?|an keinem Tag|an allen 21 Tagen

An wie vielen Tagen wollen Sie in den nächsten drei Wochen Weintrauben essen?|an keinem Tag|an allen 21 Tagen

H5. Gnocchi

An wie vielen Tagen haben Sie in den letzten drei Wochen Gnocchi gegessen?|an keinem Tag|an allen 21 Tagen

An wie vielen Tagen wollen Sie in den nächsten drei Wochen Gnocchi essen?|an keinem Tag|an allen 21 Tagen

H6. Pommes

An wie vielen Tagen haben Sie in den letzten drei Wochen Pommes gegessen?|an keinem Tag|an allen 21 Tagen

An wie vielen Tagen wollen Sie in den nächsten drei Wochen Pommes essen?|an keinem Tag|an allen 21 Tagen

H7. Waffeln

An wie vielen Tagen haben Sie in den letzten drei Wochen Waffeln gegessen?|an keinem Tag|an allen 21 Tagen

An wie vielen Tagen wollen Sie in den nächsten drei Wochen Waffeln essen?|an keinem Tag|an allen 21 Tagen

H8. Käsespätzle

An wie vielen Tagen haben Sie in den letzten drei Wochen Käsespätzle gegessen?|an keinem Tag|an allen 21 Tagen

An wie vielen Tagen wollen Sie in den nächsten drei Wochen Käsespätzle essen?|an keinem Tag|an allen 21 Tagen

H9. Speiseeis

An wie vielen Tagen haben Sie in den letzten drei Wochen Speiseeis gegessen?|an keinem Tag|an allen 21 Tagen

An wie vielen Tagen wollen Sie in den nächsten drei Wochen Speiseeis essen?|an keinem Tag|an allen 21 Tagen

H10. Sushi

An wie vielen Tagen haben Sie in den letzten drei Wochen Sushi gegessen?|an keinem Tag|an allen 21 Tagen

An wie vielen Tagen wollen Sie in den nächsten drei Wochen Sushi essen?|an keinem Tag|an allen 21 Tagen

Teil I: Konsumziele 7/9

I1. Äpfel

An wie vielen Tagen haben Sie in den letzten drei Wochen Äpfel gegessen?|an keinem Tag|an allen 21 Tagen

An wie vielen Tagen wollen Sie in den nächsten drei Wochen Äpfel essen?|an keinem Tag|an allen 21 Tagen

I2. Salat

An wie vielen Tagen haben Sie in den letzten drei Wochen Salat gegessen?|an keinem Tag|an allen 21 Tagen

An wie vielen Tagen wollen Sie in den nächsten drei Wochen Salat essen?|an keinem Tag|an allen 21 Tagen

I3. Fruchtsaft

An wie vielen Tagen haben Sie in den letzten drei Wochen Fruchtsaft getrunken?|an keinem Tag|an allen 21 Tagen

An wie vielen Tagen wollen Sie in den nächsten drei Wochen Fruchtsaft trinken?|an keinem Tag|an allen 21 Tagen

I4. Kartoffeln

An wie vielen Tagen haben Sie in den letzten drei Wochen Kartoffeln gegessen?|an keinem Tag|an allen 21 Tagen

An wie vielen Tagen wollen Sie in den nächsten drei Wochen Kartoffeln essen?|an keinem Tag|an allen 21 Tagen

I5. Leberkäse

An wie vielen Tagen haben Sie in den letzten drei Wochen Leberkäse gegessen?|an keinem Tag|an allen 21 Tagen

An wie vielen Tagen wollen Sie in den nächsten drei Wochen Leberkäse essen?|an keinem Tag|an allen 21 Tagen

I6. Nachos

An wie vielen Tagen haben Sie in den letzten drei Wochen Nachos gegessen?|an keinem Tag|an allen 21 Tagen

An wie vielen Tagen wollen Sie in den nächsten drei Wochen Nachos essen?|an keinem Tag|an allen 21 Tagen

I7. Vollmilchschokolade

An wie vielen Tagen haben Sie in den letzten drei Wochen Vollmilchschokolade gegessen?|an keinem Tag|an allen 21 Tagen

An wie vielen Tagen wollen Sie in den nächsten drei Wochen Vollmilchschokolade essen?|an keinem Tag|an allen 21 Tagen

I8. Nudeln

An wie vielen Tagen haben Sie in den letzten drei Wochen Nudeln gegessen?|an keinem Tag|an allen 21 Tagen

An wie vielen Tagen wollen Sie in den nächsten drei Wochen Nudeln essen?|an keinem Tag|an allen 21 Tagen

I9. Nougatcreme

An wie vielen Tagen haben Sie in den letzten drei Wochen Nougatcreme gegessen?|an keinem Tag|an allen 21 Tagen

An wie vielen Tagen wollen Sie in den nächsten drei Wochen Nougatcreme essen?|an keinem Tag|an allen 21 Tagen

I10. Wraps

An wie vielen Tagen haben Sie in den letzten drei Wochen Wraps gegessen?|an keinem Tag|an allen 21 Tagen

An wie vielen Tagen wollen Sie in den nächsten drei Wochen Wraps essen?|an keinem Tag|an allen 21 Tagen

Teil J: Konsumziele 8/9

J1. Kekse

An wie vielen Tagen haben Sie in den letzten drei Wochen Kekse gegessen?|an keinem Tag|an allen 21 Tagen

An wie vielen Tagen wollen Sie in den nächsten drei Wochen Kekse essen?|an keinem Tag|an allen 21 Tagen

J2. Beeren

An wie vielen Tagen haben Sie in den letzten drei Wochen Beeren gegessen?|an keinem Tag|an allen 21 Tagen

An wie vielen Tagen wollen Sie in den nächsten drei Wochen Beeren essen?|an keinem Tag|an allen 21 Tagen

J3. **Ofenbaguette**

An wie vielen Tagen haben Sie in den letzten drei Wochen Ofenbaguette gegessen?|an keinem Tag|an allen 21 Tagen

An wie vielen Tagen wollen Sie in den nächsten drei Wochen Ofenbaguette essen?|an keinem Tag|an allen 21 Tagen

J4. **Muffin**

An wie vielen Tagen haben Sie in den letzten drei Wochen Muffins gegessen?|an keinem Tag|an allen 21 Tagen

An wie vielen Tagen wollen Sie in den nächsten drei Wochen Muffins essen?|an keinem Tag|an allen 21 Tagen

J5. **süßes Gebäck**

An wie vielen Tagen haben Sie in den letzten drei Wochen süßes Gebäck gegessen?|an keinem Tag|an allen 21 Tagen

An wie vielen Tagen wollen Sie in den nächsten drei Wochen süßes Gebäck essen?|an keinem Tag|an allen 21 Tagen

J6. **Wurst**

An wie vielen Tagen haben Sie in den letzten drei Wochen Wurst gegessen?|an keinem Tag|an allen 21 Tagen

An wie vielen Tagen wollen Sie in den nächsten drei Wochen Wurst essen?|an keinem Tag|an allen 21 Tagen

J7. **Kuchen**

An wie vielen Tagen haben Sie in den letzten drei Wochen Kuchen gegessen?|an keinem Tag|an allen 21 Tagen

An wie vielen Tagen wollen Sie in den nächsten drei Wochen Kuchen essen?|an keinem Tag|an allen 21 Tagen

J8. Kichererbsen

An wie vielen Tagen haben Sie in den letzten drei Wochen Kichererbsen gegessen?|an keinem Tag|an allen 21 Tagen

An wie vielen Tagen wollen Sie in den nächsten drei Wochen Kichererbsen essen?|an keinem Tag|an allen 21 Tagen

J9. Bitterschokolade

An wie vielen Tagen haben Sie in den letzten drei Wochen Bitterschokolade gegessen?|an keinem Tag|an allen 21 Tagen

An wie vielen Tagen wollen Sie in den nächsten drei Wochen Bitterschokolade essen?|an keinem Tag|an allen 21 Tagen

J10. Bratnudeln

An wie vielen Tagen haben Sie in den letzten drei Wochen Bratnudeln gegessen?|an keinem Tag|an allen 21 Tagen

An wie vielen Tagen wollen Sie in den nächsten drei Wochen Bratnudeln essen?|an keinem Tag|an allen 21 Tagen

Teil K: Konsumziele 9/9

K1. Baguette

An wie vielen Tagen haben Sie in den letzten drei Wochen Baguette gegessen?|an keinem Tag|an allen 21 Tagen

An wie vielen Tagen wollen Sie in den nächsten drei Wochen Baguette essen?|an keinem Tag|an allen 21 Tagen

K2. Döner

An wie vielen Tagen haben Sie in den letzten drei Wochen Döner gegessen?|an keinem Tag|an allen 21 Tagen

An wie vielen Tagen wollen Sie in den nächsten drei Wochen Döner essen?|an keinem Tag|an allen 21 Tagen

K3. Toast

An wie vielen Tagen haben Sie in den letzten drei Wochen Toast gegessen?|an keinem Tag|an allen 21 Tagen

An wie vielen Tagen wollen Sie in den nächsten drei Wochen Toast essen?|an keinem Tag|an allen 21 Tagen

K4. Rindfleisch

An wie vielen Tagen haben Sie in den letzten drei Wochen Rindfleisch gegessen?|an keinem Tag|an allen 21 Tagen

An wie vielen Tagen wollen Sie in den nächsten drei Wochen Rindfleisch essen?|an keinem Tag|an allen 21 Tagen

K5. Joghurt

An wie vielen Tagen haben Sie in den letzten drei Wochen Joghurt gegessen?|an keinem Tag|an allen 21 Tagen

An wie vielen Tagen wollen Sie in den nächsten drei Wochen Joghurt essen?|an keinem Tag|an allen 21 Tagen

K6. Schokoladenwaffeln

An wie vielen Tagen haben Sie in den letzten drei Wochen Schokoladenwaffeln gegessen?|an keinem Tag|an allen 21 Tagen

An wie vielen Tagen wollen Sie in den nächsten drei Wochen Schokoladenwaffeln essen?|an keinem Tag|an allen 21 Tagen

K7. Fleischpflanzerl/ Fleischfrikadellen

An wie vielen Tagen haben Sie in den letzten drei Wochen Fleischpflanzerl/ Fleischfrikadellen gegessen?|an keinem Tag|an allen 21 Tagen

An wie vielen Tagen wollen Sie in den nächsten drei Wochen Fleischpflanzerl/ Fleischfrikadellen essen?|an keinem Tag|an allen 21 Tagen

K8. Gemüselaibchen/ Gemüsefrikadellen

An wie vielen Tagen haben Sie in den letzten drei Wochen Gemüselaibchen/ Gemüsefrikadellen gegessen?|an keinem Tag|an allen 21 Tagen

An wie vielen Tagen wollen Sie in den nächsten drei Wochen Gemüselaibchen/ Gemüsefrikadellen essen?|an keinem Tag|an allen 21 Tagen

K9. Vollkornbrot

An wie vielen Tagen haben Sie in den letzten drei Wochen Vollkornbrot gegessen?|an keinem Tag|an allen 21 Tagen

An wie vielen Tagen wollen Sie in den nächsten drei Wochen Vollkornbrot essen?|an keinem Tag|an allen 21 Tagen

K10. Schokoladenriegel

An wie vielen Tagen haben Sie in den letzten drei Wochen Schokoladenriegel gegessen?|an keinem Tag|an allen 21 Tagen

An wie vielen Tagen wollen Sie in den nächsten drei Wochen Schokoladenriegel essen?|an keinem Tag|an allen 21 Tagen

Vielen Dank für Ihre Teilnahme an der ersten Befragung. Wir werden die erhobenen Daten zu Ihren Konsumzielen nun auswerten und uns bei Ihnen melden, um Sie über die weitere Studienteilnahme zu informieren und einen Termin für die Besprechung der Hauptstudienphase zu vereinbaren.

Sie können dieses Fenster nun schließen.
