# Supplementary material for: Testing the effectiveness of a mobile approach avoidance intervention and measuring approach biases in an ecological momentary assessment context: study protocol for a randomised-controlled trial
Source: BMJ Open. 2023 Apr 25;13(4):e070443. doi: 10.1136/bmjopen-2022-070443 (PMC10151942; doi:10.1136/bmjopen-2022-070443)
Supplement: Supplementary data [file bmjopen-2022-070443supp004.pdf]

UNIVERSITÄT SALZBURG
Fachbereich Psychologie
Centre for Cognitive Neuroscience,
Fachbereich für Psychologie

Univ.-Prof. Dr. Jens Blechert
jens.blechert@sbg.ac.at
Tel.: +43 / (0) 662 /8044 - 5163
Fax.: +43 / (0) 662 / 8044 – 5126

Hellbrunnerstraße 34
A-5020 Salzburg – Austria

<u>TeilnehmerInneninformation und Einwilligungserklärung</u>

zur Teilnahme an der Studie:

‚Kognitiv-Affektive Mechanismen von Essregulationstrainings ‘

Interne Bezeichnung: Cognitive-affective mechanisms of food biases trainings (AAI)

Liebe/r Interessent/in,

wir laden Sie ein an der oben genannten Studie teilzunehmen.

Im Folgenden finden Sie einige Informationen, die für Sie von Bedeutung sind, wenn Sie sich für eine Teilnahme interessieren.

Bitte unterschreiben Sie diese Einverständniserklärung nur

- wenn Sie Art und Ablauf der Studie verstanden haben
- wenn Sie bereit sind, einer Teilnahme zuzustimmen und
- wenn Sie sich über Ihre Rechte als Teilnehmer im Klaren sind.

Allgemeine Informationen und Ziele der Studie

Die Ernährungspsychologie beschäftigt sich mit Grundlagen und Behandlungsmöglichkeiten von ungesunden Essensentscheidungen sowie mit Ess- und Gewichtsstörungen. Da das Verlangen nach schmackhaften Nahrungsmitteln das Essverhalten im Alltag beeinflusst, ist es wichtig, dass wir ein besseres Verständnis für diese Zusammenhänge gewinnen, auch um Abhilfe verschaffen zu können. In dieser Studie geht es speziell darum, wie automatische Annäherungstendenzen zu Essensreizen optimal erfasst werden können, was die zugrundeliegenden Mechanismen sind und wie das Annäherungsverhalten reduziert werden kann.

Ablauf der Studie

Bei Ihrer Studienteilnahme werden wir Sie bitten, verschiedene Fragebögen zum psychischen Befinden auszufüllen. Des Weiteren wird Ihre Teilnahme nachfolgende Prozeduren beinhalten:

Aulbach MB, *et al. BMJ Open* 2023; 13:e070443. doi: 10.1136/bmjopen-2022-070443

- *Betrachten von Bildern von Nahrungsmitteln*: Sie werden verschiedene Nahrungsmittel am Bildschirm betrachten und bewerten.
- *Reaktionszeitmessung via Smartphone*: Sie werden gebeten, mit schnellen Bewegungen des Smartphones auf Bilder von Nahrungsmitteln zu reagieren.
- *Fragebögen via Smartphone*: Zweimal pro Tag werden Sie gebeten über das Smartphone verschiedene Fragen zu Ihrem momentanen Empfinden und Ihrem vergangenen Essverhalten zu beantworten.
- *Reaktionszeittraining in einer von zwei Gruppen.* Ergänzend zu den oben beschriebenen Studienteilen werden Sie zudem per Zufallsauswahl *einer von zwei Studiengruppen zugeteilt*. Aus wissenschaftlichen Gründen ist eine zufällige Gruppenzuteilung notwendig, Sie können sich also nicht eine der Gruppen aussuchen. Beide Gruppen beinhalten die Bearbeitung von Reaktionszeitaufgaben am Smartphone über mehrere Termine hinweg, jedoch unterscheidet sich die Art der Reaktionszeitaufgabe geringfügig. Am Ende der Studie erklären wir Ihnen ausführlich die Hintergründe der Trainings.

Freiwilligkeit der Teilnahme

Ihre Teilnahme an dieser Erhebung ist freiwillig. Sie können diese jederzeit, ohne Angabe von Gründen, beenden. Die Ablehnung Ihrer Teilnahme oder ein vorzeitiges Beenden kann mündlich oder schriftlich (siehe Kontaktdaten am Ende des Dokuments) erfolgen, hat keine nachteiligen Folgen und Sie werden anteilig kompensiert. Sie können Ihre Einwilligung zur Speicherung der Daten bis zum Ende der Datenerhebung widerrufen, ohne dass Ihnen daraus Nachteile entstehen.

Ausschlusskriterien

Sie können an unserer Studie teilnehmen, wenn Sie zwischen 18 und 60 Jahren alt sind und ausreichende Deutschkenntnisse besitzen, um die Instruktionen während der Studie zu verstehen. Nicht teilnehmen können außerdem Personen, die schwanger sind, in ihrer Entscheidungsfähigkeit eingeschränkt sind oder wenn eine diagnostizierte Essstörung vorliegt.

Pflichten bei einer Teilnahme

Als Studienteilnehmer/-innen werden Sie gebeten, den Anweisungen der Studienleitung so weit wie möglich zu folgen und die Fragebögen aufrichtig zu beantworten. Sollten sich während der Studie Probleme und Schwierigkeiten ergeben, informieren Sie bitte die Studienleitung darüber.

Rechte bei einer Teilnahme

Ihre Teilnahme erfolgt freiwillig und Sie können sich jederzeit, auch ohne Angabe von Gründen, aus der Studie zurückziehen, und/ oder eine Löschung der erfassten Daten verlangen, ohne dass Ihnen daraus Nachteile irgendwelcher Art entstehen.

Nutzen bei einer Teilnahme

Ihre Teilnahme an dieser Studie dient der klinisch-psychologischen Wissenschaft. Neue Erkenntnisse sollen zum besseren Verständnis der Ursachen von Überessen und starkem Verlangen nach schmackhaftem Essen beitragen. Außerdem profitieren Sie durch die Teilnahme an dem Reaktionszeittraining bezüglich Ihres

Aulbach MB, *et al. BMJ Open* 2023; 13:e070443. doi: 10.1136/bmjopen-2022-070443

selbstgesteckten Ernährungsziels. Nach Studienende erhalten Sie auf Wunsch ausführliche schriftliche Informationen über Sinn und Zweck der Studie sowie eine individualisierte Rückmeldung und Erklärung ihrer Ergebnisse. Des Weiteren erhalten Sie eine Aufwandsentschädigung.

Aufwandsentschädigung

Während wir davon ausgehen, dass Ihr Essverhalten vom Reaktionszeittraining profitieren wird, entsteht durch die Beantwortung der Fragebögen jedoch zusätzlicher zeitlicher Aufwand, für den wir sie finanziell kompensieren. Abhängig von der Vollständigkeit ihr Daten erhalten Sie sechs bis acht VP-Stunden beziehungsweise zwischen 40 und 60 Euro.

Mögliche Risiken und Unannehmlichkeiten

Die oben beschriebenen Prozeduren sind nicht gesundheitsschädlich und entsprechen wissenschaftlichen Standards. Sie können vorrübergehend negative physische und emotionale Empfindungen hervorrufen. Diese Empfindungen sowie spätere diesbezügliche Erinnerungen sind jedoch erfahrungsgemäß vorübergehender Natur. Sollten Sie unerwarteter Weise unter anhaltenden Belastungen aufgrund Ihrer Studienteilnahme leiden, so melden Sie sich umgehend bei einer der unten aufgeführten Kontaktpersonen.

Vertraulichkeit und Schutz der Daten

Bei den Daten ist zu unterscheiden zwischen personenbezogenen Daten, mit denen Sie direkt identifizierbar sind (z.B.: Name, Telefonnummer, Adresse), und pseudonymisierten (verschlüsselten) Daten, bei denen alle Informationen, die direkten Rückschluss auf Ihre Identität zulassen, durch einen Teilnahmecode ersetzt werden. Der **‚Schlüssel'** (Abgleich von Pseudonym mit personenbezogenen Daten) ist nur der Studienleitung zugänglich, wird nach dem Stand der Technik in passwort-geschützten Dokumenten geschützt und getrennt von den verschlüsselten Datensätzen aufbewahrt. Sämtliche Personen, die Zugang zu personenbezogenen Daten erhalten, unterliegen im Umgang mit den Daten dem österreichischen und europäischen Datenschutzrecht und sind dem Datengeheimnis verpflichtet.

Im Rahmen dieser Studie erfolgt die Verarbeitung von folgenden Daten:
- *Personenbezogenen Daten*: Name, E-mail, Telefonnummer. Diese Daten erleichtern uns die Studienadministration und die Sicherstellung einer hohen Datenqualität.
- *Pseudonymisierte Daten*: Alle Eingaben, die Sie über die App ‚m-Path' machen, werden dort unter einem selbstgewählten Teilnahmecode („Pseudonym") auf Servern der Universität Leuven (Belgien) gespeichert. Ihre Daten sind also innerhalb der App nicht Ihrer Person zu zuordnen. Auch die Daten in der App ‚Picture Game' werden nur über einen pseudonymisierten Teilnahmecode gespeichert und über einen Account der Studienleitung von Google firebase übertragen. Für beide Apps gilt: Eine Zuordnung zu Ihrer Person ist nur mit zusätzlichen Daten möglich, die ausschließlich Mitarbeitenden der PLUS vorliegen (‚Schlüssel', siehe oben).

Aulbach MB, *et al. BMJ Open* 2023; 13:e070443. doi: 10.1136/bmjopen-2022-070443

Ihnen steht bezüglich Ihrer bei uns gespeicherten pseudonymisierten Daten grundsätzlich das Recht auf Auskunft, Richtigstellung, Löschung und Einschränkung zu. Sie können Ihre Einwilligung auch jederzeit widerrufen. Ein Widerruf hat zur Folge, dass wir Ihre Daten ab diesem Zeitpunkt zu den oben genannten Zwecken nicht mehr verarbeiten. Für einen Widerruf wenden Sie sich bitte an die unten angeführte Studienleitung. Wenn Sie glauben, dass wir gegen datenschutzrechtliche Vorschriften verstoßen, können Sie sich bei dem Datenschutzbeauftragten der Universität Salzburg (datenschutz@sbg.ac.at) oder bei einer Datenschutzbehörde beschweren. Weitere Informationen finden Sie unter www.uni-salzburg.at/impressum.

Wir weisen darauf hin, dass wissenschaftliche Publikationen geplant sind und dabei Studienergebnisse in ausschließlich anonymer Weise veröffentlicht werden.

Die personenbezogenen Daten und der zugehörige Schlüssel wird **1 Jahr nach Studienende gelöscht**. Ebenso werden alle Daten in der App zu dem Zeitpunkt gelöscht. Damit erlöschen auch ihre Möglichkeiten der Einsicht/Berichtigung/Export/Löschung dieser Daten, da wir Sie ihren Daten dann nicht mehr zuordnen können (da der ‚Schlüssel' gelöscht wurde). Wir behalten einzig ihren Namen und diese Einverständniserklärung als Dokumentation ihrer Studienteilname für **30 Jahre**.

Welche Kontaktpersonen stehen zur Verfügung?

Bei anfälligen Fragen, die während oder nach Abschluss der Studie auftreten, können Sie sich unter gesundheitspsychologie07@plus.ac.at oder der +43 677 616 767 07 melden und jederzeit an die Studienleitung wenden:

- Hannah van Alebeek, MSc., Fachbereich Psychologie, Universität Salzburg, Hellbrunnerstrasse 34, 5020 Salzburg, hannah.vanalebeek@plus.ac.at

Wir freuen uns auf die Zusammenarbeit!

Diese Studie wurde von der Ethikkommission der Universität Salzburg evaluiert.
Kontaktperson: Mag.[a] Clara Gröblacher, Kapitelgasse 4, A-5020 Salzburg, Tel: +43-662-8044 2391, clara.groeblacher@sbg.ac.at

Einverständniserklärung

Ich habe die oben beschriebenen Informationen vollumfänglich gelesen und verstanden.

Meine Teilnahme erfolgt freiwillig und ich weiß, dass ich mich jederzeit, auch ohne Angabe von Gründen, von der Studie zurückziehen kann, ohne dass mir daraus Nachteile irgendwelcher Art entstehen.

Ich bin bereit, an dieser Studie teilzunehmen und bin mit der Erhebung und Verwendung persönlicher Daten nach Maßgabe der TeilnehmerInneninformation einverstanden.

Ort/Datum ______________________________

Name TeilnehmerIn ______________________________

Unterschrift TeilnehmerIn ______________________________

Unterschrift
Studienverantwortliche Person ______________________________

Aulbach MB, *et al. BMJ Open* 2023; 13:e070443. doi: 10.1136/bmjopen-2022-070443

– Dieses Dokument bleibt bei der Versuchsleitung –

Einverständniserklärung

Ich habe die oben beschriebenen Informationen vollumfänglich gelesen und verstanden. Außerdem nehme ich zur Kenntnis, dass ich für Wege zum/vom Untersuchungsort, nicht wege-/unfallversichert bin.

Meine Teilnahme erfolgt freiwillig und ich weiß, dass ich mich jederzeit, auch ohne Angabe von Gründen, von der Studie zurückziehen kann, ohne dass mir daraus Nachteile irgendwelcher Art entstehen.

Ich bin bereit, an dieser Studie teilzunehmen und bin mit der Erhebung und Verwendung persönlicher Daten nach Maßgabe der TeilnehmerInneninformation einverstanden. Eine Kopie dieser Studieninformation und Einverständniserklärung wurde mir ausgehändigt.

Ort/Datum ______________________________

Name TeilnehmerIn ______________________________

Unterschrift TeilnehmerIn ______________________________

Unterschrift
Studienverantwortliche Person ______________________________

Aulbach MB, *et al. BMJ Open* 2023; 13:e070443. doi: 10.1136/bmjopen-2022-070443
