# Supplementary material for: Testing the effectiveness of a mobile approach avoidance intervention and measuring approach biases in an ecological momentary assessment context: study protocol for a randomised-controlled trial
Source: BMJ Open. 2023 Apr 25;13(4):e070443. doi: 10.1136/bmjopen-2022-070443 (PMC10151942; doi:10.1136/bmjopen-2022-070443)
Supplement: Author's manuscript [file bmjopen-2022-070443.draft_revisions.pdf]

BMJ Open

BMJ Open is committed to open peer review. As part of this commitment we make the peer review history of every article we publish publicly available.

When an article is published we post the peer reviewers' comments and the authors' responses online. We also post the versions of the paper that were used during peer review. These are the versions that the peer review comments apply to.

The versions of the paper that follow are the versions that were submitted during the peer review process. They are not the versions of record or the final published versions. They should not be cited or distributed as the published version of this manuscript.

BMJ Open is an open access journal and the full, final, typeset and author-corrected version of record of the manuscript is available on our site with no access controls, subscription charges or pay-per-view fees (http://bmjopen.bmj.com).

If you have any questions on BMJ Open's open peer review process please email info.bmjopen@bmj.com

BMJ Open

A Randomized-Controlled Trial on Mobile Approach Avoidance Training for Measuring and Changing Food Preferences in Everyday Life in an EMA context

Journal:	*BMJ Open*
Manuscript ID	bmjopen-2022-070443
Article Type:	Protocol
Date Submitted by the Author:	23-Nov-2022
Complete List of Authors:	Aulbach, Matthias; Paris Lodron Universitat Salzburg, Department of Psychology<br>van Alebeek, Hannah; Centre for Cognitive Neuroscience, Department of Psychology<br>Kahveci, Sercan; Centre for Cognitive Neuroscience, Department of Psychology<br>Blechert, Jens; Centre for Cognitive Neuroscience, Department of Psychology
Keywords:	Information technology < BIOTECHNOLOGY & BIOINFORMATICS, World Wide Web technology < BIOTECHNOLOGY & BIOINFORMATICS, NUTRITION & DIETETICS, PREVENTIVE MEDICINE, PUBLIC HEALTH

SCHOLARONE™
Manuscripts

A Randomized-Controlled Trial on Mobile Approach Avoidance Training for Measuring and Changing Food Preferences in Everyday Life in an EMA context

Matthias Burkard Aulbach[1]*

Hannah van Alebeek[1]

Sercan Kahveci[1]

Jens Blechert[1]

[1]Department of Psychology, Centre for Cognitive Neuroscience, Paris-Lodron-University of Salzburg, Salzburg, Austria

*Corresponding author; contact: Matthias.aulbach@plus.ac.at

For peer review only

Word count: 4159 words

Keywords: randomized controlled trial; approach-avoidance training; eating behavior; intervention; ecological momentary assessment; m-health

ABSTRACT

Introduction: Unhealthy eating behavior is a major contributor to obesity and related diseases and may be driven by automatic approach tendencies towards tasty but unhealthy foods. Approach-Avoidance interventions (AAI) have been proposed as a remedy to retrain approach biases and help people to eat in line with their dietary goals. Mobile implementations of AAI might represent a useful, low threshold intervention but its effectiveness has not been established yet.

Methods and analysis: Participants with the goal of changing their eating behavior are randomized to intervention or control groups, each completing six sessions of a smartphone-based AAI, in which they push (i.e., avoid) or pull (i.e., approach) personalized food images. Intervention group participants always avoid individualized sets of foods that they want to eat less often and approach foods that they want to eat more often. In the control group, response directions and images are paired equally often. To evaluate contextual and dynamic intervention effects, ecological momentary assessment (EMA) is measured throughout, with questions about food intake, hunger, stress, emotions, eating intentions, food craving, and impulsivity twice a day. Additional EMA pre- and post-intervention phases are measured before and after the intervention phase (4 days each) with a one-day follow-up EMA four weeks after the intervention.

Multilevel models will examine the temporal covariance between approach bias and self-reported variables as well as short- and long-term intervention effects on approach bias, food intake, and craving.

Ethics and dissemination: The study was approved by the Ethics Committee of the University of Salzburg. Results will be published in peer reviewed scientific journals and presented at scientific conferences.

Trial registration: This study was registered at the German Clinical Trials Register DRKS, registration number DRKS00030780, and on the Open Science Framework https://osf.io/4k3q9/?view_only=4db6431fd5ee4148a97f3be7f799ea4a.

Strengths and limitations of this study

- The study is a randomized controlled trial testing an m-health intervention to assist participants in implementing their dietary intentions by re-training their automatic approach bias towards to-be-reduced foods with an Approach-Avoidance intervention (AAI) against a closely matched active control task
- It includes ecological momentary assessment before, during, and after the trial in both control and intervention groups, which allows examining both short- and long-term intervention effects
- It measures a range of potentially relevant phenomena like food craving, hunger, emotions, stress, and day-level impulsivity
- Control group data allows examining variability in approach bias and its covariation with data obtained through EMA
- Measures of food intake are restricted to single-item daily self-reports which are prone to underreporting and experimenter demand

For peer review only

Humans make several decisions about whether, what, and how much to eat per day [1]. All these decisions have an influence on human health, as eating too many of some foods can lead to obesity and related diseases [2]. It is therefore of paramount importance to understand which factors contribute to eating decisions and how we can intervene on them. Traditional psychological models have postulated that people reflect on behavioral options, form intentions, and then translate these into behavior. However, decades of research have shown that intentions are often not successfully enacted, a phenomenon termed the "intention behavior gap" [3].

Impulsive processes may be a factor in hindering the implementation of intentions. These happen automatically, that is, quickly, often outside of conscious awareness, are difficult to control, and might bypass intention formation [4–7] and are typically assessed with computer tasks based on the measurement of reaction times (RT) [8,9]. Under certain conditions, such indirect measures of food preference can increase prediction accuracy of actual behavior, above and beyond questionnaire data [6,10]. One example of such an RT task is the Approach Avoidance Task (AAT). In the AAT, participants usually use a joystick [or in more recent studies, a touch-screen; ,11,12] to perform movements towards or away from different stimulus categories. These categories, such as foods and non-food objects, are compared on how fast they are approached and avoided and this RT difference indicates the strength of an approach-avoidance bias towards some category over another [13]. Food approach-avoidance biases seem relevant to real-world eating behavior, as they are higher in people who strongly crave foods [14] and relate to increased food consumption in impulsive individuals and in people who are prone to external or emotional eating [15,16].

Earlier studies have already shown that approach bias [17], food choice [18] and subsequent food intake [19] can be reduced through Approach-Avoidance Interventions (AAI) that predominantly pair one food category with avoidance and the other with approach movements. The mixed evidence in this domain [20–24], however, might have different reasons. Firstly, in some studies participants approach and avoid stimuli based on their category (e.g., food vs objects) and in others, based on an irrelevant feature of the stimulus such as the frame color or orientation. While such task differences affect reliability of bias measures [25], they may also affect participants' awareness about the contingencies and expectations about training effects, potentially important features influencing the effectiveness of AAIs [26]. Secondly, the personal relevance of the trained stimuli may differ between interventions. While some interventions specifically try to retrain approach biases to chocolates in individuals reporting high trait-level chocolate craving or consumption [17], other interventions train responses to a pre-selected set of healthy and unhealthy foods without taking into account if unhealthy foods are actually consumed across participants or if healthy foods fit to individual needs of the participants (for example in terms of taste, food intolerances and so on).Thirdly, most studies only deliver a single session of AAI (with Meule, Richard, et al., 2019 being an exception) and more sessions might lead to larger effects that would be easier to detect [28]. Finally, the effectiveness of AAIs might depend on the time of intervention delivery. If approach biases vary over time, it is easy to see that interventions might be most fruitful in moments when the bias is strong [as hypothesized for social anxiety by 29], or just before a tempting eating situation.

This possible fluctuation of biases over time has thus far been mostly ignored. In most studies to date, approach bias has, at least implicitly, been treated as a relatively stable, trait-like phenomenon. Approach bias is typically measured at a single timepoint and then correlated with other phenomena like trait food craving [12], weight status [30], or eating disorder diagnosis [31]. One recent study demonstrated that approach bias was independent of experimentally induced satiety, indicating stability of the bias across situations. However, participants' desire to eat specific

For peer review only

foods did explain variance in approach bias, implying that bias might vary across time within individuals depending on their current consumption desires [32]. This is in line with the finding that approach bias for chocolate was positively correlated with current chocolate craving [25,33,34], craving being an experience of intense desire for a specific food which is temporally variable by definition [35]. Other studies using a mobile version of the AAT indicated that test-retest reliability across eight measurement occasions was low while split-half reliability was high, again indicating temporal fluctuations in approach biases [36]. This is in line with findings obtained from other indirect measures that showed modest stability over time [37]. Such within-subject fluctuations in biases are probably not due to random variation or measurement error as every-day biases have been shown to decrease with after-meal-satiety in normal-weight individuals and bias size changes based on peoples current affective states [38–42]. In combination, these results raise questions about the trait- or state-like nature of approach biases.

Smartphone-based AATs and AAIs are therefore interesting for a range of research questions that cannot be answered with stationary computer-based tasks. Firstly, smartphones allow relatively easy delivery of AAI to participants during their daily routines. This is helpful not only for participants to perform the intervention task repeatedly but also to bring the intervention temporally closer to "high-risk" situations in everyday life. Assuming a rather fast decay of intervention effects, this should enhance effectiveness compared to conducting a training intervention on one's personal computer [43] or in a laboratory session [44,45]. Another advantage of repeated intervention through smartphones is the possibility to measure immediate and delayed intervention effects on fluctuating phenomena like food craving in participants' real-world environment. Secondly, bias can be more easily measured at any time of the day, and especially at moments when it may be relevant for food consumption. This allows us to examine the temporal and situational variability of approach bias, shedding light on its trait- vs. state-like components. Combining it with repeated delivery of eating-related questions throughout the day (i.e., ecological momentary assessment, EMA) also allows for correlating fluctuations in approach bias with other temporally variable phenomena like food craving, affect and intake.

Several studies have provided interventions using computer tasks through the internet [43,44,46–48] and have generally reported good compliance rates and effects on dietary intake. Smartphone-based interventions using similar RT tasks are much rarer and have reported mixed results on key outcomes [27,28,49,50]. One of the two studies delivering smartphone-based AAI requiring tilt movements of the smartphone as responses found positive effects on food choice and approach bias towards unhealthy foods [49]. The other study reported neither day-level nor longer-term effects of AAI using swipe movements compared to an EMA-only intervention and a sham training group [27]. It is important to note that that study did not find any approach bias in participants to begin with and that the sample size was small. It is therefore unclear to what degree its results can be taken as evidence against the effectiveness of mobile AAI.

One recent AAT variant does not require swiping movements on the touchscreen, but instead requires participants to physically move the phone towards or away from themselves while viewing food stimuli [36,51–53]. For measurement purposes, this task has been shown to be a valid tool to study food approach biases outside the laboratory and to provide relevant information beyond self-report measures [51]. In addition to RTs, it also yields data on the forcefulness of the movements which might contain relevant information not captured by RTs [53].

The study presented here sets out to (1) test its effectiveness as an intervention tool when programmed to deliver AAI, that is, to pair foods a specific participant wants to eat more often with approach and foods a specific participant wants to eat less often with avoidance responses.

For peer review only - http://bmjopen.bmj.com/site/about/guidelines.xhtml

Specifically, we will study to what degree the intervention can support participants in their goal of changing their eating behavior and (2) to further examine the reliability and validity of approach bias scores obtained through a phone-delivered AAT. Combining the AAT with the repeated measurement of related phenomena through EMA allows us to disentangle short- and long-term intervention effects outlined in aim (1) and to closely study aim (2).

METHODS AND ANALYSIS

Study Overview

The study uses a two-arm, double blind randomized controlled trial conducted with German speaking participants and is coordinated at the University of Salzburg, Austria. It compares an active Approach-Avoidance Intervention (AAI) to a sham-training (a measurement Approach-Avoidance Task, AAT) in its impact on eating behavior, food liking, food craving, and food approach bias.

Participants

Participants will be recruited via university emailing lists, social networks, university events, and word of mouth. Participants are required to be between 18 and 60 years of age, and not to be pregnant or report a diagnosis for an eating disorder. Importantly, participants are required to have an intention to change their eating behavior.

We calculated power using the data of a previous, unpublished study from our group with a medium effect size (g = .56); a multilevel model was fitted examining the pre- to post-training difference in approach-avoidance bias between individuals undergoing actual training and sham training. Between 50 and 200 participants were sampled randomly with replacement in steps of 10, with 10 samplings per sample size. The significance of the Time × Group effect was tallied per sample size. The resulting power curve indicated 80% of random samples were significant at sample sizes of 120 and above (60 per group). We opted to err on the safer side and chose to recruit a sample of n = 150 valid subjects, after applying exclusion criteria (75 per group). With an estimated recruitment rate of three participants per week and allowing for recruitment difficulties slowing down the process, we expect data collection to last from November 2022 to roughly January 2024. However, data collection continues until 150 participants are reached.

Materials

Baseline questionnaires. In a web-based questionnaire, participants give informed consent and then indicate their age, gender, nationality, state of employment, highest achieved formal education level, dietary restrictions (vegan, vegetarian, pescetarian, omnivorous, other), height, weight, and possible food allergies or intolerances. Participants identifying as female or diverse are also asked about their menstrual cycle. Further, to assess exclusion criteria, they are asked if they are currently suffering from an eating disorder. This is followed by the stimulus selection (see below for details). Participants then complete the German versions of the following questionnaires: subscales restrained eating and external eating from the Dutch Eating Behavior Questionnaire (DEBQ; van Strien et al., 1986); the Salzburg Emotional Eating Scale (SEES; Meule et al., 2018); the Salzburg Stress Eating Scale (SEES; Meule et al., 2018b); Perceived Self-Regulatory Success in Dieting (PSRS; Meule et al., 2012); the short version of the UPPS Impulsivity Scale [58].

Stimuli. At the beginning of the study, participants rate a pre-selected set of 90 food and drink images from the food.pics [59] and CROCUFID [60] databases, and from freely available online resources on two scales: "In the last three weeks, on how many days have you eaten/drunk this food/drink?" (recent intake) and "In the next three weeks, on how many days would you like to

For peer review only

eat/drink this food/drink?" (intended intake). We select the six foods with the most negative difference between past and intended consumption as "increase foods" (eaten less often than intended). Then, among the foods that were eaten at least on six days in the past three weeks, we select six with the most positive difference between past and intended future consumption (eaten more often than intended = "decrease-foods"). A randomly selected four of these six images are then used in the intervention phase in both groups while the other two were left untrained to test whether the intervention would generalize and show an effect on untrained foods. A random selection of 8 out of a set of 12 images of non-food items serves as control stimuli. Figure 1 displays the selection of stimuli.

Setup call. Within a few days of filling out the online questionnaire, a member of the study team contacts participants to schedule a setup call via phone or a video conferencing tool. In this call, the member of the research team explains the procedure of the study. They further help participants install the necessary apps onto their smartphone (m-path; KU Leuven, 2022 for EMA and the AAT app [1]) and then confirm the correct selection of approach and avoidance foods as determined by the rating task. After the call, participants receive a manual for the study via email which summarizes the study procedures and the use of the smartphone applications and includes participants' individual three-digit identification code as well as contact information of the study team.

Approach-Avoidance Task (AAT). We use the smartphone-based AAT as introduced by Zech and colleagues [53]. In this version of the task, participants see stimuli on their horizontally held phone screen and approach/avoidance movements are performed by physically moving the phone towards/away from themselves (see Figure 2 and two short introductory videos here: https://osf.io/4k3q9/?view_only=4db6431fd5ee4148a97f3be7f799ea4a). Each trial starts with a fixation dot in the middle of a white screen, which is followed by either one of the food or object stimuli after a 1500 ms delay. While correct approach or avoidance responses make the picture disappear and trigger the start of a new trial, incorrect responses are followed by a 2000 ms display of a black error-cross. If a participant does not respond for 2000 ms, a clock icon is displayed indicating timeout.

The active and sham AAT trainings feature 4 out of 6 approach-foods, 4 out of 6 avoid-foods, as well as 8 out of 12 control object stimuli. The training sessions consist of four training blocks of 16 trials each, and each training block is preceded by 4 practice trials, yielding a training session of 64 training trials and 16 practice trials, or 80 trials total. The pre-, post- and follow-up bias assessment AATs similarly consist of four blocks preceded each by four practice trials but feature all selected images (6 increase-foods, 6 decrease-foods and 12 objects). All 24 images are presented one time per block, yielding 96 test trials and 16 practice trials, or 112 trials total. In all AATs, the instructions of the blocks alternate such that participants are instructed to approach foods while avoiding objects in the first block (approach-food blocks) and avoid foods while approaching objects in the second block (avoid-food blocks). This order is the same for all sessions and all participants. Crucially, in the active training AATs, only approach-foods are shown in the approach-food blocks, while only avoid-foods are shown in the avoid-food blocks; sham training instead features both approach- and avoid-foods during both approach-food and avoid-food blocks.

[1] The app can be downloaded on Android devices from this address: https://play.google.com/store/apps/details?id=com.eatlabsbg.eatapp

Table 1: overview of EMA questions (own translations from German). All items are answered on a slider from 0 to 100 unless indicated differently in parentheses. Midday prompts remain the same across the entire study duration. Evening prompts differ depending on the study phase as indicated:
* These items are asked alongside a food image and are repeated for each image (six “increase” and six “decrease” images).
[+] This item is only asked after the first and the last AAT session (day 5 and 15).

**Item**	**Midday (all study phases)**	**Evening Days 1-4 and 17-20**	**Evening Days 5-16**	**Evening - follow-up**
When was the last time you ate something?	X			
What type of meal was it? (breakfast, lunch, dinner, snack)	X			
How hungry are you right now?	X	X	X	
Do you feel like you have everything under control?	X			
Do you feel like you are on top of things?	X			
How optimistic do you feel right now?	X			X
How happy do you feel right now?	X			X
How lonely do you feel right now?	X			X
How depressed do you feel right now?	X			X
How angry do you feel right now?	X			X
How mad do you feel right now?	X			X
How tense do you feel right now?	X			X
How anxious do you feel right now?	X			X
How much do you want to stick to your dietary goals *for the rest of the day*?	X			
How much do you want to stick to your dietary goals *tomorrow*?		X		X
How strong has your craving for this food been *today*? *		X		X
How much of this food have you eaten *today*? *		X		X
Have you said things today without thinking?	X	X	X	X

For peer review only

Have you spent more money today than you wanted to?	X	X	X	X
Have you felt impatient today?	X	X	X	X
Have you made a spontaneous decision today?	X	X	X	X
How strong has your craving for this food been *since the midday questionnaire*? *			X	
How much of this food have you eaten *since the midday questionnaire*? *			X	
How much do you expect that this task will help you reach your dietary goals? [+]	X[+]			
Throughout the whole study, how often did you push this food away from yourself? *				X
Please indicate the day your last period started.				X

For peer review only

Ecological Momentary Assessment (EMA). Participants follow the EMA schedule for a total of 20 days. During the whole period, participants receive two prompts per day (delivered through the smartphone application m-path [61], one just before the time a participant usually eats lunch and the other in the evening (exact time agreed-upon with the participant to represent an end-of-day signal). Table 1 shows the questions that participants answer on those prompts and Figure 3 displays the temporal sequence of the study. EMA prompts on days 1-3 of the study only contain the listed questions. On day 4 (the day before the start of the intervention) and day 17 (the day after the end of the intervention), participants receive an instruction to open the AAT application and complete a measurement AAT. On every second day during the intervention phase (days 5 through 16), participants receive an instruction to open the AAT application and complete a training AAT after completing the midday prompt. 30 minutes after completing the midday prompt, participants receive a notification asking whether they conducted the training. On replying "yes", they receive positive feedback; on replying "no", they are asked to now open the AAT app to conduct the task. In addition, on day 6 and day 16 (the first and the last day including an AAT/AAI session), participants further indicate their expectancy of how much the task will help them reach their dietary goals. Four weeks after the end of the initial 20-day EMA period, participants receive one additional EMA questionnaire and a measurement AAT in the evening. After performing this final AAT, participants indicate how often they believed they pushed or pulled each of their "decrease-foods" and "increase-foods".

Procedure. The procedure for study participation is as follows: after interested participants contact the study team, they receive an individual participant code and a weblink to the baseline questionnaire. At this point, an R-script [2] randomizes participants to either the intervention or control group with the condition unknown to the study team. After a setup call with a member of the research team within a few days of filling out the questionnaires, participants start receiving EMA prompts and AAT as described above. Figure 3 shows the timeline of the whole study. Throughout the study period, participants can contact study personnel who also monitor compliance to the EMA and AAT schedule and contact participants in case of low compliance.

Outcomes

Main outcomes. This study uses three main outcomes: First, participants' self-report intake of "increase" and "decrease" foods according to the EMA schedule outlined above on a slider from 0 (labelled "nothing") to 100 ("very much"). Second, participants self-report craving for those same foods in the same manner.

Third, we measure approach biases for all selected foods based on the RT and force in the AAT. The RT will be defined as the time from picture to movement onset. Force will be defined as the peak acceleration in the correct direction during a trial, standardized within participant by dividing every individual's measurement of force by the participant-specific standard deviation. Separately for approach and avoidance trials as well as for sessions, the RT and the force will be averaged across the four AAT blocks for each specific food stimuli. For objects, we will also average across the different stimuli. The average approach or avoid response for objects on a session will be subtracted from stimulus-specific food approach or avoidance response on that session to achieve food-specific *single-difference* approach and avoidance scores according to these formulas:

Stimulus-specific approach = [food-specific approach]-[average object approach]

---

[2] The function *sample* randomly outputs the number "1" or "2" which correspond to the conditions.

For peer review only - http://bmjopen.bmj.com/site/about/guidelines.xhtml

Stimulus-specific avoidance = [food-specific avoidance]-[average object avoidance]

Double-difference scores will be used to achieve a full bias score per food stimulus and session: ([food-specific avoidance]-[food-specific approach])-([average object avoidance]-[average object approach]).

Secondary outcomes. Dietary intentions are measured according to the outlined EMA schedule. Intention-behavior gaps (IBGs) are calculated as the difference between intentions and actual food intake for all applicable time periods.

Further assessments. Further, we assess day-level impulsivity with the four item Momentary Impulsivity Scale [62]. Perceived contingency between foods and required reactions is reported with a single item per food image at follow-up. Belief in the effectiveness of the intervention is assessed after the first and last session of AAT/AAI.

Data analysis plan

Data analysis will serve to investigate a series of research questions relating to different aspects of the study. Further detail on the data analysis is available in the pre-registration at https://osf.io/4k3q9/?view_only=4db6431fd5ee4148a97f3be7f799ea4a.

Data exclusion. For analyses regarding the effectiveness of the AAI, we will exclude participants who did not conduct any of the AATs during the intervention phase as we regard them as "not treated". For the remaining participants, sensitivity analyses will be performed to test whether the number of completed training sessions affects intervention effectiveness. For analyses of approach bias, we will exclude error trials and trials with reaction times that deviate more than +/-3SD from the individual mean of the participant in that AAT session. If more than 25% of trials must be excluded based on these criteria, the whole AAT session will be excluded from further analyses. This post-hoc session exclusion does not affect whether a participant will be counted as "not treated" or not in the analyses regarding the effectiveness of the AAI.

Overall intervention effectiveness. The first set of research questions relates to the effectiveness of the intervention as compared to the control condition from pre- to post-training. To this end, we use multilevel models to predict intake of trained "increase" and "decrease" foods as a function of timepoint (three days pre- vs post-intervention), condition (intervention vs control), and their interaction. Equivalent models test the intervention effect on approach biases towards - and craving for - the two food categories. To test to what degree the effect of the training intervention generalizes to non-trained foods, we then use data from trained and untrained stimuli and add a variable that indicates whether a food appeared in the training or not (trained vs untrained) and all interaction terms to the model. This will be followed up with tests to determine whether changes in the approach bias are mainly driven by changes in approach- or avoidance RTs. We further test the moderating role of intentions, baseline stimulus craving, and person-level variables obtained from the questionnaires, as well as contingency awareness and expectancy by adding the relevant variable and its interaction terms to the equations. Finally, we examine the mediating effect of craving for changes in intake.

Immediate intervention effectiveness. The second set of research questions concerns the short-term effects of the intervention during the intervention phase (days 5-16). Multilevel models predicting food intake and cravings, respectively, will include the factors group (intervention vs control) and (off-)training day (training day vs no training day) as predictors. In another pair of multilevel models, we use group and the number of days since the beginning of the intervention and

For peer review only - http://bmjopen.bmj.com/site/about/guidelines.xhtml

their interaction as predictors of craving and food intake, respectively. The force applied during the training will be used as a predictor for the change in craving and intake from before the start of the training.

Trait and state components of approach bias. The third set of research questions relates to the state and trait components of approach bias and will be examined within the control group only. This is because only participants in the control group receive measurement AATs throughout the study period. Multilevel models will test whether bias size and negative emotions are related on both a between-subjects and a within-subjects level and to what degree this depends on the strength of the desire for these foods. A separate model tests equivalent research questions for the relation between bias strength and craving and bias strength and intake, respectively. The later analyses testing how bias strength is related to subsequent food intake will be expanded by including trait and day-level impulsivity and day-level intentions of regulating food intake

Ethics, dissemination, and data handling.

The study received ethical approval from the ethics board of the University of Salzburg and is conducted in accordance with the declaration of Helsinki. Results of the trial will be disseminated through a series of articles in appropriate scientific journals and conference presentations.

Data will be handled confidentially and stored in a pseudonymized manner. Neither m-path nor the AAT application collect personal data but work through three-digit identification codes assigned to participants. The identification key linking personal data to the identification codes will be kept in password-protected files separately from the pseudonymized data and will be destroyed one year after termination of the study. Deidentified data will be archived for at least ten years and consent forms as documentation of participation will be archived for 30 years. The deidentified data will be made publicly on the Open Science Framework after the completion of planned publications.

Patient and public involvement

Patients and the public are not involved in study design, data collection, data analysis, or dissemination.

For peer review only

Contributorship statement

MBA: conceptualisation, methodology, writing first draft of the protocol, revising the protocol
HvA: conceptualisation, methodology, analysis plan, reviewing drafts of the protocol
SK: conceptualisation, methodology, analysis plan, reviewing drafts of the protocol
JB: conceptualisation, methodology, supervision, reviewing drafts of the protocol
all authors read and approved the final version of the protocol.

Competing interests

All authors report no competing interests.

Funding

This work was supported by FWF grant number P 34542-B. The funder plays no role in the study design; collection, management, analysis, and interpretation of data; writing of the report; and the decision to submit the report for publication.

References

1 Wansink B, Sobal J. Mindless Eating: The 200 Daily Food Decisions We Overlook. *Environ Behav* 2007;**39**:106–23. doi:10.1177/0013916506295573

2 World Health Organization. Obesity and Overweight. Published Online First: 2018. doi:10.1007/978-3-319-33228-4_447

3 Sheeran P, Webb TL. The Intention–Behavior Gap. *Soc Personal Psychol Compass* 2016;**10**:503–18. doi:10.1111/spc3.12265

4 Hagger MS. Non-conscious processes and dual-process theories in health psychology. *Health Psychol Rev* 2016;**10**:375–80. doi:10.1080/17437199.2016.1244647

5 Hagger MS, Chatzisarantis NLD. An Integrated Behavior Change Model for Physical Activity. *Exerc Sport Sci Rev* 2014;**42**:62–9. doi:10.1249/JES.0000000000000008

6 Hofmann W, Friese M, Wiers RW. Impulsive versus reflective influences on health behavior: a theoretical framework and empirical review. *Health Psychol Rev* 2008;**2**:111–37. doi:10.1080/17437190802617668

7 Sheeran P, Bosch JA, Crombez G, *et al.* Implicit processes in health psychology: Diversity and promise. *Health Psychol* 2016;**35**:761–6. doi:10.1037/hea0000409

8 Gawronski B, De Houwer J. Implicit measures in social and personality psychology. In: *Handbook of research methods in social and personality psychology*. Cambridge University Press 2014. 283–310.http://hdl.handle.net/1854/LU-2959505 (accessed 29 Apr 2020).

9 Gawronski B, Hahn A. IMPLICIT MEASURES Procedures, Use, and Interpretation. In: Blanton H, LaCroix JM, Webster GD, eds. *Measurement in Social Psychology*. New York, NY: : Yaylor & Francis 2019. 29–55.http://www.bertramgawronski.com/documents/GH2019Measurement.pdf

10 Hagger MS, Trost N, Keech J, *et al.* Predicting sugar consumption: Application of an integrated dual-process, dual-phase model. *Appetite* Published Online First: 2017. doi:10.1016/j.appet.2017.04.032

For peer review only

11 Kahveci S, van Alebeek H, Berking M, *et al.* Touchscreen-based assessment of food approach biases: investigating reliability and item-specific preferences. *Appetite* 2021;:105190. doi:10.1016/j.appet.2021.105190

12 Meule A, Lender A, Richard A, *et al.* Approach–avoidance tendencies towards food: Measurement on a touchscreen and the role of attention and food craving. *Appetite* 2019;**137**:145–51.

13 Rinck M, Becker ES. Approach and avoidance in fear of spiders. *J Behav Ther Exp Psychiatry* 2007;**38**:105–20. doi:10.1016/j.jbtep.2006.10.001

14 Brockmeyer T, Hahn C, Reetz C, *et al.* Approach bias and cue reactivity towards food in people with high versus low levels of food craving. *Appetite* 2015;**95**:197–202. doi:10.1016/j.appet.2015.07.013

15 Kakoschke N, Kemps E, Tiggemann M. Impulsivity moderates the effect of approach bias modification on healthy food consumption. *Appetite* 2017;**117**:117–25. doi:10.1016/j.appet.2017.06.019

16 Kakoschke N, Kemps E, Tiggemann M. Differential effects of approach bias and eating style on unhealthy food consumption in overweight and normal weight women. *Psychol Health* 2017;**32**:1371–85. doi:10.1080/08870446.2017.1327587

17 Dickson H, Kavanagh DJDJ, MacLeod C. The pulling power of chocolate: Effects of approach-avoidance training on approach bias and consumption. *Appetite* 2016;**99**:46–51. doi:10.1016/j.appet.2015.12.026

18 Veling H, Verpaalen IAM, Liu H, *et al.* How can food choice best be trained? Approach-avoidance versus go/no-go training. *Appetite* 2021;:105226. doi:10/gjkp4t

19 Schumacher SE, Kemps E, Tiggemann M. Bias modification training can alter approach bias and chocolate consumption. *Appetite* 2016;**96**:219–24. doi:10.1016/j.appet.2015.09.014

20 Aulbach MB, Knittle K, Haukkala A. Implicit process interventions in eating behaviour: a meta-analysis examining mediators and moderators. *Health Psychol Rev* 2019;**13**:179–208. doi:10.1080/17437199.2019.1571933

21 Becker D, Jostmann NB, Wiers RW, *et al.* Approach avoidance training in the eating domain: Testing the effectiveness across three single session studies. *Appetite* 2015;**85**:58–65. doi:10.1016/j.appet.2014.11.017

22 Becker D, Jostmann NB, Holland RW. *Does approach bias modification really work in the eating domain? A commentary on Kakoschke et al. (2017)*. 2018. doi:10.1016/j.addbeh.2017.02.025

23 Navas JF, Verdejo-García A, Vadillo MA. The evidential value of research on cognitive training to change food-related biases and unhealthy eating behavior: A systematic review and p-curve analysis. *Obes Rev* 2021;**22**:e13338. doi:10.1111/obr.13338

24 Yang Y, Shields GS, Wu Q, *et al.* Cognitive training on eating behaviour and weight loss: A meta-analysis and systematic review. *Obes Rev* 2019;**0**. doi:10.1111/obr.12916

For peer review only

25 Lender A, Meule A, Rinck M, *et al.* Measurement of food-related approach–avoidance biases: Larger biases when food stimuli are task relevant. *Appetite* 2018;**125**:42–7. doi:10.1016/j.appet.2018.01.032

26 Van Dessel P, De Houwer J, Gast A. Approach–Avoidance Training Effects Are Moderated by Awareness of Stimulus–Action Contingencies. *Pers Soc Psychol Bull* 2016;**42**:81–93. doi:10.1177/0146167215615335

27 Meule A, Richard A, Dinic R, *et al.* Effects of a Smartphone-Based Approach-Avoidance Intervention on Chocolate Craving and Consumption: Randomized Controlled Trial. *JMIR MHealth UHealth* 2019;**7**:e12298. doi:10.2196/12298

28 Aulbach MB, Knittle K, van Beurden SB, *et al.* App-based food Go/No-Go training: User engagement and dietary intake in an opportunistic observational study. *Appetite* 2021;**165**:105315. doi:10/gmvjtq

29 Boettcher J, Leek L, Matson L, *et al.* Internet-Based Attention Bias Modification for Social Anxiety: A Randomised Controlled Comparison of Training towards Negative and Training Towards Positive Cues. *PLOS ONE* 2013;**8**:e71760. doi:10.1371/journal.pone.0071760

30 Kemps E, Tiggemann M. Approach Bias for Food Cues in Obese Individuals. *Psychol Health* 2014;**0446**:1–25. doi:10.1080/08870446.2014.974605

31 Neimeijer RAM, de Jong PJ, Roefs A. Automatic approach/avoidance tendencies towards food and the course of anorexia nervosa. *Appetite* 2015;**91**:28–34. doi:http://dx.doi.org/10.1016/j.appet.2015.03.018

32 Kahveci S, Meule A, Lender A, *et al.* Food approach bias is moderated by the desire to eat specific foods. *Appetite* 2020;**154**:104758. doi:10.1016/j.appet.2020.104758

33 Kemps E, Tiggemann M, Martin R, *et al.* Implicit approach-avoidance associations for craved food cues. *J Exp Psychol Appl* 2013;**19**:30–8. doi:10.1037/a0031626

34 Wittekind CE, Blechert J, Schiebel T, *et al.* Comparison of different response devices to assess behavioral tendencies towards chocolate in the approach-avoidance task. *Appetite* 2021;**165**:105294. doi:10.1016/j.appet.2021.105294

35 Weingarten HP, Elston D. The phenomenology of food cravings. *Appetite* 1990;**15**:231–46.

36 Zech HG, Gable P, van Dijk WW, *et al.* Test-retest reliability of a smartphone-based approach-avoidance task: Effects of retest period, stimulus type, and demographics. *Behav Res Methods* Published Online First: 1 August 2022. doi:10.3758/s13428-022-01920-6

37 Gawronski B, Morrison M, Phills CE, *et al.* Temporal Stability of Implicit and Explicit Measures: A Longitudinal Analysis. *Pers Soc Psychol Bull* 2017;**43**:300–12. doi:10.1177/0146167216684131

38 Krehbiel J, Halbeisen G, Kühn S, *et al.* Too hot to handle: Mood states moderate implicit approach vs. avoidance tendencies toward food cues in patients with obesity and active binge eating disorder. *J Psychiatr Res* 2021;**143**:302–8. doi:10.1016/j.jpsychires.2021.09.031

39 Neimeijer RAM, Roefs A, Ostafin BD, *et al.* Automatic Approach Tendencies toward High and Low Caloric Food in Restrained Eaters: Influence of Task-Relevance and Mood. *Front Psychol* 2017;**8**.https://www.frontiersin.org/articles/10.3389/fpsyg.2017.00525 (accessed 24 Oct 2022).

For peer review only

40 Richard A, Meule A, Blechert J. When and how do explicit measures of food craving predict implicit food evaluation? A moderated mediation model. *Food Qual Prefer* 2018;**66**:141–7. doi:10.1016/J.FOODQUAL.2018.01.018

41 Richard A, Meule A, Blechert J. Implicit evaluation of chocolate and motivational need states interact in predicting chocolate intake in everyday life. *Eat Behav* 2019;**33**:1–6. doi:10.1016/j.eatbeh.2019.01.006

42 Zech HG. Food approach dynamics in daily live: Speed and force of food approach movements fluctuate with hunger, but less so for people with high BMI. 2022.

43 Lawrence NS, O'Sullivan J, Parslow D, *et al.* Training response inhibition to food is associated with weight loss and reduced energy intake. *Appetite* 2015;**95**:17–28. doi:10.1016/j.appet.2015.06.009

44 Stice E, Yokum S, Veling H, *et al.* Pilot test of a novel food response and attention training treatment for obesity: Brain imaging data suggest actions shape valuation. *Behav Res Ther* 2017;**94**:60–70. doi:10.1016/j.brat.2017.04.007

45 Stice E, Yokum S, Gau J, *et al.* Efficacy of a food response and attention training treatment for obesity: A randomized placebo controlled trial. *Behav Res Ther* 2022;**158**:104183. doi:10.1016/j.brat.2022.104183

46 Forman EM, Manasse SM, Dallal DH, *et al.* Computerized neurocognitive training for improving dietary health and facilitating weight loss. *J Behav Med* Published Online First: 2019. doi:10.1007/s10865-019-00024-5

47 Poppelaars A, Scholten H, Granic I, *et al.* When winning is losing: A randomized controlled trial testing a video game to train food-specific inhibitory control. *Appetite* 2018;**129**:143–54. doi:10.1016/j.appet.2018.06.039

48 Veling H, van Koningsbruggen GMGM, Aarts H, *et al.* Targeting impulsive processes of eating behavior via the internet. Effects on body weight. *Appetite* 2014;**78**:102–9. doi:10.1016/j.appet.2014.03.014

49 Kakoschke N, Hawker C, Castine B, *et al.* Smartphone-based cognitive bias modification training improves healthy food choice in obesity: A pilot study. *Eur Eat Disord Rev* 2018;**26**:526–32. doi:10.1002/erv.2622

50 Keeler JL, Chami R, Cardi V, *et al.* App-based food-specific inhibitory control training as an adjunct to treatment as usual in binge-type eating disorders: A feasibility trial. *Appetite* 2022;**168**:105788.

51 Brouwer A-M, van Beers JJ, Sabu P, *et al.* Measuring Implicit Approach–Avoidance Tendencies towards Food Using a Mobile Phone outside the Lab. *Foods* 2021;**10**:1440. doi:10.3390/foods10071440

52 van Beers JJ, Kaneko D, Stuldreher IV, *et al.* An Accessible Tool to Measure Implicit Approach-Avoidance Tendencies Towards Food Outside the Lab. In: *Companion Publication of the 2020 International Conference on Multimodal Interaction*. Virtual Event Netherlands: : ACM 2020. 307–11. doi:10.1145/3395035.3425647

For peer review only

53 Zech HG, Rotteveel M, van Dijk WW, *et al.* A mobile approach-avoidance task. *Behav Res Methods* 2020;**52**:2085–97. doi:10.3758/s13428-020-01379-3

54 van Strien T, Frijters JER, Bergers GPA, *et al.* The Dutch Eating Behavior Questionnaire (DEBQ) for assessment of restrained, emotional, and external eating behavior. *Int J Eat Disord* 1986;**5**:295–315. doi:10.1002/1098-108X(198602)5:2<295::AID-EAT2260050209>3.0.CO;2-T

55 Meule A, Reichenberger J, Blechert J. Development and Preliminary Validation of the Salzburg Emotional Eating Scale. *Front Psychol* 2018;**9**.https://www.frontiersin.org/articles/10.3389/fpsyg.2018.00088 (accessed 18 Oct 2022).

56 Meule A, Reichenberger J, Blechert J. Development and preliminary validation of the Salzburg Stress Eating Scale. *Appetite* 2018;**120**:442–8. doi:10.1016/j.appet.2017.10.003

57 Meule A, Papies EK, Kübler A. Differentiating between successful and unsuccessful dieters. Validity and reliability of the Perceived Self-Regulatory Success in Dieting Scale. *Appetite* 2012;**58**:822–6. doi:10.1016/j.appet.2012.01.028

58 Whiteside SP, Lynam DR, Miller JD, *et al.* Validation of the UPPS impulsive behaviour scale: a four-factor model of impulsivity. *Eur J Personal* 2005;**19**:559–74.

59 Blechert J, Lender A, Polk S, *et al.* Food-Pics_Extended—An Image Database for Experimental Research on Eating and Appetite: Additional Images, Normative Ratings and an Updated Review. *Front Psychol* 2019;**10**:307. doi:10.3389/fpsyg.2019.00307

60 Toet A, Kaneko D, de Kruijf I, *et al.* CROCUFID: A Cross-Cultural Food Image Database for Research on Food Elicited Affective Responses. *Front Psychol* 2019;**10**:58. doi:10.3389/fpsyg.2019.00058

61 KU Leuven. m-Path – Free tool for blended care by KU Leuven. 2022.https://m-path.io/landing/ (accessed 18 Oct 2022).

62 Tomko RL, Solhan MB, Carpenter RW, *et al.* Measuring impulsivity in daily life: the momentary impulsivity scale. *Psychol Assess* 2014;**26**:339.

For peer review only

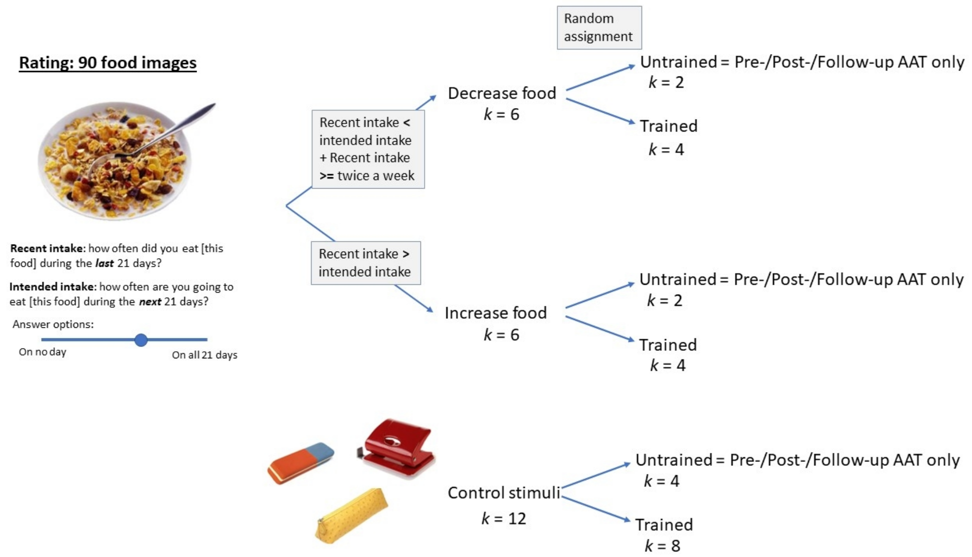

Figure 1: selection of the images presented in the training.

338x190mm (96 x 96 DPI)

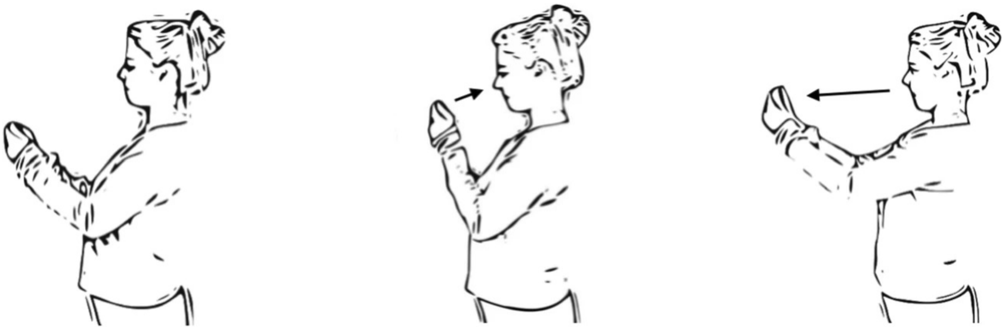

Figure 2 (reproduced from Zech et al., 2020): movements in the mobile Approach-Avoidance task. The image on the left shows the starting position, the image in the middle an approach trial and the image on the right an avoidance trial

274x89mm (120 x 120 DPI)

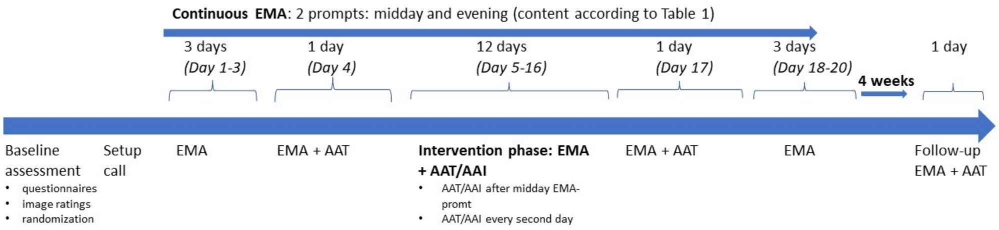

Figure 3: time schedule of the whole study period.

338x110mm (96 x 96 DPI)

Reporting checklist for protocol of a clinical trial.

Based on the SPIRIT guidelines.

Instructions to authors

Complete this checklist by entering the page numbers from your manuscript where readers will find each of the items listed below.

Your article may not currently address all the items on the checklist. Please modify your text to include the missing information. If you are certain that an item does not apply, please write "n/a" and provide a short explanation.

Upload your completed checklist as an extra file when you submit to a journal.

In your methods section, say that you used the SPIRITreporting guidelines, and cite them as:

Chan A-W, Tetzlaff JM, Gøtzsche PC, Altman DG, Mann H, Berlin J, Dickersin K, Hróbjartsson A, Schulz KF, Parulekar WR, Krleža-Jerić K, Laupacis A, Moher D. SPIRIT 2013 Explanation and Elaboration: Guidance for protocols of clinical trials. BMJ. 2013;346:e7586

		Reporting Item	Page Number
**Administrative information**			
Title	#1	Descriptive title identifying the study design, population, interventions, and, if applicable, trial acronym	1
Trial registration	#2a	Trial identifier and registry name. If not yet registered,	2

For peer review only

		name of intended registry	
Trial registration: data set	#2b	All items from the World Health Organization Trial Registration Data Set	n/a
Protocol version	#3	Date and version identifier	n/a
Funding	#4	Sources and types of financial, material, and other support	12
Roles and responsibilities: contributorship	#5a	Names, affiliations, and roles of protocol contributors	12
Roles and responsibilities: sponsor contact information	#5b	Name and contact information for the trial sponsor	1
Roles and responsibilities: sponsor and funder	#5c	Role of study sponsor and funders, if any, in study design; collection, management, analysis, and interpretation of data; writing of the report; and the decision to submit the report for publication, including whether they will have ultimate authority over any of these activities	12
Roles and responsibilities: committees	#5d	Composition, roles, and responsibilities of the coordinating centre, steering committee, endpoint adjudication committee, data management team, and other individuals or groups overseeing the trial, if applicable (see Item 21a for data monitoring committee)	n/a

Introduction

For peer review only

Background and rationale	#6a	Description of research question and justification for undertaking the trial, including summary of relevant studies (published and unpublished) examining benefits and harms for each intervention	3-5
Background and rationale: choice of comparators	#6b	Explanation for choice of comparators	3-5
Objectives	#7	Specific objectives or hypotheses	5
Trial design	#8	Description of trial design including type of trial (eg, parallel group, crossover, factorial, single group), allocation ratio, and framework (eg, superiority, equivalence, non-inferiority, exploratory)	5-7
**Methods: Participants, interventions, and outcomes**			
Study setting	#9	Description of study settings (eg, community clinic, academic hospital) and list of countries where data will be collected. Reference to where list of study sites can be obtained	5-8
Eligibility criteria	#10	Inclusion and exclusion criteria for participants. If applicable, eligibility criteria for study centres and individuals who will perform the interventions (eg,	5

For peer review only

		surgeons, psychotherapists)	
Interventions: description	#11a	Interventions for each group with sufficient detail to allow replication, including how and when they will be administered	6
Interventions: modifications	#11b	Criteria for discontinuing or modifying allocated interventions for a given trial participant (eg, drug dose change in response to harms, participant request, or improving / worsening disease)	n/a
Interventions: adherance	#11c	Strategies to improve adherence to intervention protocols, and any procedures for monitoring adherence (eg, drug tablet return; laboratory tests)	5
Interventions: concomitant care	#11d	Relevant concomitant care and interventions that are permitted or prohibited during the trial	n/a
Outcomes	#12	Primary, secondary, and other outcomes, including the specific measurement variable (eg, systolic blood pressure), analysis metric (eg, change from baseline, final value, time to event), method of aggregation (eg, median, proportion), and time point for each outcome. Explanation of the clinical relevance of chosen efficacy and harm outcomes is strongly recommended	9-10
Participant timeline	#13	Time schedule of enrolment, interventions (including any run-ins and washouts), assessments, and visits for participants. A schematic diagram is highly recommended (see Figure)	9

For peer review only

Sample size	#14	Estimated number of participants needed to achieve study objectives and how it was determined, including clinical and statistical assumptions supporting any sample size calculations	5
Recruitment	#15	Strategies for achieving adequate participant enrolment to reach target sample size	5
**Methods: Assignment of interventions (for controlled trials)**			
Allocation: sequence generation	#16a	Method of generating the allocation sequence (eg, computer-generated random numbers), and list of any factors for stratification. To reduce predictability of a random sequence, details of any planned restriction (eg, blocking) should be provided in a separate document that is unavailable to those who enrol participants or assign interventions	9
Allocation concealment mechanism	#16b	Mechanism of implementing the allocation sequence (eg, central telephone; sequentially numbered, opaque, sealed envelopes), describing any steps to conceal the sequence until interventions are assigned	9
Allocation: implementation	#16c	Who will generate the allocation sequence, who will enrol participants, and who will assign participants to interventions	9

For peer review only

Blinding (masking)	#17a	Who will be blinded after assignment to interventions (eg, trial participants, care providers, outcome assessors, data analysts), and how	9
Blinding (masking): emergency unblinding	#17b	If blinded, circumstances under which unblinding is permissible, and procedure for revealing a participant's allocated intervention during the trial	n/a
**Methods: Data collection, management, and analysis**			
Data collection plan	#18a	Plans for assessment and collection of outcome, baseline, and other trial data, including any related processes to promote data quality (eg, duplicate measurements, training of assessors) and a description of study instruments (eg, questionnaires, laboratory tests) along with their reliability and validity, if known. Reference to where data collection forms can be found, if not in the protocol	6-10
Data collection plan: retention	#18b	Plans to promote participant retention and complete follow-up, including list of any outcome data to be collected for participants who discontinue or deviate from intervention protocols	6-10
Data management	#19	Plans for data entry, coding, security, and storage, including any related processes to promote data quality (eg, double data entry; range checks for data values).	10

For peer review only

		Reference to where details of data management procedures can be found, if not in the protocol	
Statistics: outcomes	#20a	Statistical methods for analysing primary and secondary outcomes. Reference to where other details of the statistical analysis plan can be found, if not in the protocol	9-10
Statistics: additional analyses	#20b	Methods for any additional analyses (eg, subgroup and adjusted analyses)	9-10
Statistics: analysis population and missing data	#20c	Definition of analysis population relating to protocol non-adherence (eg, as randomised analysis), and any statistical methods to handle missing data (eg, multiple imputation)	9-10
**Methods: Monitoring**			
Data monitoring: formal committee	#21a	Composition of data monitoring committee (DMC); summary of its role and reporting structure; statement of whether it is independent from the sponsor and competing interests; and reference to where further details about its charter can be found, if not in the protocol. Alternatively, an explanation of why a DMC is not needed	n/a
Data monitoring: interim analysis	#21b	Description of any interim analyses and stopping guidelines, including who will have access to these interim results and make the final decision to terminate the trial	n/a
Harms	#22	Plans for collecting, assessing, reporting, and managing solicited and spontaneously reported adverse events and other unintended effects of trial interventions or trial	n/a

For peer review only

		conduct	
Auditing	#23	Frequency and procedures for auditing trial conduct, if any, and whether the process will be independent from investigators and the sponsor	n/a
**Ethics and dissemination**			
Research ethics approval	#24	Plans for seeking research ethics committee / institutional review board (REC / IRB) approval	2
Protocol amendments	#25	Plans for communicating important protocol modifications (eg, changes to eligibility criteria, outcomes, analyses) to relevant parties (eg, investigators, REC / IRBs, trial participants, trial registries, journals, regulators)	n/a
Consent or assent	#26a	Who will obtain informed consent or assent from potential trial participants or authorised surrogates, and how (see Item 32)	6
Consent or assent: ancillary studies	#26b	Additional consent provisions for collection and use of participant data and biological specimens in ancillary studies, if applicable	n/a
Confidentiality	#27	How personal information about potential and enrolled participants will be collected, shared, and maintained in order to protect confidentiality before, during, and after the trial	10
Declaration of	#28	Financial and other competing interests for principal	12

For peer review only

interests		investigators for the overall trial and each study site	
Data access	#29	Statement of who will have access to the final trial dataset, and disclosure of contractual agreements that limit such access for investigators	11
Ancillary and post trial care	#30	Provisions, if any, for ancillary and post-trial care, and for compensation to those who suffer harm from trial participation	n/a
Dissemination policy: trial results	#31a	Plans for investigators and sponsor to communicate trial results to participants, healthcare professionals, the public, and other relevant groups (eg, via publication, reporting in results databases, or other data sharing arrangements), including any publication restrictions	11
Dissemination policy: authorship	#31b	Authorship eligibility guidelines and any intended use of professional writers	n/a
Dissemination policy: reproducible research	#31c	Plans, if any, for granting public access to the full protocol, participant-level dataset, and statistical code	11
**Appendices**			
Informed consent materials	#32	Model consent form and other related documentation given to participants and authorised surrogates	n/a
Biological specimens	#33	Plans for collection, laboratory evaluation, and storage of biological specimens for genetic or molecular analysis in the current trial and for future use in ancillary studies, if applicable	n/a

For peer review only

The SPIRIT Explanation and Elaboration paper is distributed under the terms of the Creative Commons Attribution License CC-BY-NC. This checklist was completed on 23. November 2022 using https://www.goodreports.org/, a tool made by the EQUATOR Network in collaboration with Penelope.ai

For peer review only

BMJ Open

Study protocol for a Randomized-Controlled Trial to Test the Effectiveness of a Mobile Approach Avoidance Intervention and to Measure Approach Biases in an EMA context

Journal:	*BMJ Open*
Manuscript ID	bmjopen-2022-070443.R1
Article Type:	Protocol
Date Submitted by the Author:	03-Mar-2023

Complete List of Authors:	Aulbach, Matthias; Paris Lodron Universitat Salzburg, Department of Psychology<br>van Alebeek, Hannah; Centre for Cognitive Neuroscience, Department of Psychology<br>Kahveci, Sercan; Centre for Cognitive Neuroscience, Department of Psychology<br>Blechert, Jens; Centre for Cognitive Neuroscience, Department of Psychology

**Primary Subject Heading**:	Nutrition and metabolism
Secondary Subject Heading:	Public health
Keywords:	Information technology < BIOTECHNOLOGY & BIOINFORMATICS, World Wide Web technology < BIOTECHNOLOGY & BIOINFORMATICS, NUTRITION & DIETETICS, PREVENTIVE MEDICINE, PUBLIC HEALTH

SCHOLARONE™ Manuscripts

Study protocol for a Randomized-Controlled Trial to Test the Effectiveness of a Mobile Approach Avoidance Intervention and to Measure Approach Biases in an EMA context

Matthias Burkard Aulbach[1]*

Hannah van Alebeek[1]

Sercan Kahveci[1]

Jens Blechert[1]

[1]Department of Psychology, Centre for Cognitive Neuroscience, Paris-Lodron-University of Salzburg, Salzburg, Austria

*Corresponding author; contact: Matthias.aulbach@plus.ac.at

For peer review only

Word count: 4433 words

Keywords: randomized controlled trial; approach-avoidance training; eating behavior; intervention; ecological momentary assessment; m-health

ABSTRACT

Introduction: Unhealthy eating behavior is a major contributor to obesity and related diseases and is associated with a behavioral bias to approach rather than avoid desired foods, as measured with reaction time tasks. Approach-Avoidance interventions (AAI) have been proposed as a way to modify food evaluations and help people to eat in accordance with their dietary goals. Mobile implementations of AAI might be easily accessible, low threshold interventions, but their effectiveness has not been established yet.

Methods and analysis: Participants who aim to change their eating behavior are randomized to intervention or control groups. They complete six sessions of a smartphone-based AAI, in which they push (i.e., avoid) or pull (i.e., approach) personalized food images. Intervention group participants always avoid foods that they personally want to eat less often and approach foods that they personally want to eat more often. In the control group, images are paired equally often with both response directions. To evaluate contextual and dynamic intervention effects, ecological momentary assessment (EMA) is measured throughout, with questions about food intake, hunger, stress, emotions, eating intentions, food craving, and impulsivity twice a day. Additional EMA pre- and post-intervention measures are administered before and after the intervention phase (4 days each) with a one-day follow-up EMA four weeks after the intervention. Multilevel models will examine the temporal covariance between approach bias and self-reported variables as well as short- and long-term intervention effects on approach bias, food intake, and craving.

Ethics and dissemination: The study was approved by the Ethics Committee of the University of Salzburg. Results will be published in peer reviewed scientific journals and presented at scientific conferences.

Trial registration: This study was registered at the German Clinical Trials Register DRKS, registration number DRKS00030780, and on the Open Science Framework https://osf.io/yn7kt.

Strengths and limitations of this study

- The study is a randomized controlled trial testing an m-health intervention to assist participants in implementing their dietary intentions through repeated use of a mobile Approach-Avoidance Intervention (AAI) compared with a closely matched active control task
- It includes ecological momentary before, during, and after the trial in both control and intervention groups, which allows examining both short- and long-term intervention effects
- It measures a range of potentially relevant phenomena like food craving, hunger, emotions, stress, and day-level impulsivity
- Control group data allows examining variability in approach bias and its covariation with data obtained through EMA
- Measures of food intake are restricted to single-item daily self-reports which are prone to underreporting and experimenter demand

For peer review only

Humans make several decisions per day about whether, what, and how much to eat [1]. All these decisions have an influence on human health, as overeating can lead to obesity and related diseases [2]. It is therefore important to understand which factors contribute to eating decisions and how we can intervene on them. Traditional psychological models have postulated that people reflect on behavioral options, form intentions, and then translate these into behavior. However, decades of research have shown that intentions are often not successfully enacted, a phenomenon termed the “intention behavior gap” [3].

To investigate why people sometimes fail to transfer their intentions into behavior, researchers have devised a range of indirect measures, typically assessed with computer tasks based on the measurement of reaction times (RT) [4,5], as opposed to direct measures such as self-reported evaluations of stimuli. Under certain conditions, such indirect measures of food preference can increase prediction accuracy of actual behavior, above and beyond questionnaire data [6,7] but see [8] for a critical discussion. One example of such an RT task is the Approach Avoidance Task (AAT). In the AAT, participants usually use a joystick [or in more recent studies, a touch-screen; ,9,10] to perform movements towards or away from different stimulus categories. These categories, such as foods and non-food objects, are compared on how fast they are approached and avoided, and this RT difference is termed the “approach bias” [11]. Herein, we adopt an operational definition, such that we define approach bias as the relative speed with which one can approach the target stimuli (e.g., foods) in the AAT, remaining agnostic to what might be the underlying mental construct [8,12,13]. Food approach biases seem relevant to real-world eating behavior, as they have been found to be higher in people who strongly crave foods [14], and they relate to increased food consumption in impulsive individuals and in people who are prone to external or emotional eating [15,16] (but see [17] for contradictory findings from the alcohol domain and a wider discussion in [18,19]).

So far, it has mostly been ignored that approach biases may fluctuate over time. In most studies to date, approach bias has, at least implicitly, been treated as a relatively stable, trait-like phenomenon, in line with its conceptualization as a (stable) mental construct. Approach bias is typically measured at a single timepoint and then correlated with other phenomena like trait food craving [10], weight status [20], or eating disorder diagnosis [21]. One recent study demonstrated that approach bias was independent of experimentally induced satiety, indicating stability of the bias across situations. However, participants’ desire to eat specific foods did explain variance in approach bias, implying that bias might vary across time within individuals depending on their current consumption desires [22]. This is in line with the finding that approach bias for chocolate was positively correlated with current chocolate craving [23–25], craving being an experience of intense desire for a specific food which is temporally variable by definition [26]. Other studies using a mobile version of the AAT indicated that test-retest reliability across eight measurement occasions was low while split-half reliability was high, again indicating temporal fluctuations in approach biases [27]. This is in line with findings obtained from other indirect measures that showed modest stability over time [28]. Such within-subject fluctuations in biases are probably not only due to random variation, as approach-avoidance biases have been shown to decrease with after-meal-satiety in normal-weight individuals, and they have been shown to change based on individuals’ current affective states [29–33]. In combination, these results raise questions about the temporal and situational stability of approach biases.

Associations between behavioral approach bias and intake-related variables have led to the development of Approach-Avoidance Interventions (AAI). Traditionally, it has been assumed that a stable mental construct thought to underlie the behavioral approach bias, can be changed by

For peer review only

repeatedly pairing unhealthy foods with avoidance and healthy foods (or neutral objects) with approach. This should then affect food intake. How exactly AAIs might work is however a matter of current debate. Based on the idea that the behavioral approach bias operationalizes learned associations between appetitive stimuli and approach, some authors argue that the repeated pairing of appetitive stimuli and avoidance weakens or reverses this association through the formation of new associations between stimuli, movement direction, and evaluative properties inherent to approach and avoidance [23]. Others however argue that stimulus value is updated due to a conflict between evaluation and within-task behavior which then influences intake decisions [34–36], or that altered behavior is due to changed food evaluations, which are caused by cognitive inferences based on task requirements [37,38]. Independent of what might be the exact working mechanism, earlier studies have shown that the behavioral approach bias [39], food choice [40] and subsequent food intake [41] can be reduced by AAIs. The evidence in this domain is mixed, however [42–46], and might have several different reasons.

Firstly, in some studies participants approach and avoid stimuli based on their category (e.g., food vs objects) and in others, based on an irrelevant feature of the stimulus such as the frame color or orientation. Such task differences may affect participants' awareness of the contingencies between stimulus and required response, as well as expectations about training effects. As this awareness could increase the effectiveness of the intervention, especially when AAIs change behavior through cognitive inferences as noted above [47], this study uses a relevant-feature AAT/AAI and closely tracks participants' contingency awareness. Secondly, the personal relevance of the trained stimuli may differ between interventions, and this may influence effectiveness [48]. Some interventions specifically try to retrain approach biases to chocolate in individuals reporting high trait-level chocolate craving or consumption [39], while other interventions train responses to a pre-selected set of healthy and unhealthy foods without taking into account if participants actually consume the unhealthy foods or if healthy foods fit to individual needs of the participants (for example in terms of taste, food intolerances). Thirdly, most studies only deliver a single session of AAI (with [49,50] being exceptions), while more sessions might lead to larger effects that would be easier to detect [51]. Finally, the effectiveness of AAIs might depend on when the intervention is delivered. It is easy to see that interventions might be most fruitful in or just before moments when the risk for unhealthy intake is high.

Smartphone-based AATs and AAIs are interesting for a range of research questions that cannot be answered with stationary computer-based AATs and AAIs. Firstly, smartphones allow easy delivery of AAI to participants during their daily routines. This helps participants to perform the intervention repeatedly, and to bring the intervention temporally and spatially closer to "high-risk" situations in everyday life. Assuming a rather fast decay of intervention effects, the closer proximity offered by the smartphone should enhance its effectiveness compared to conducting it on one's personal computer [52] or in a laboratory session [53,54]. Another advantage of repeated intervention through smartphones is the possibility to measure immediate and delayed intervention effects on fluctuating phenomena like food craving. Lastly, smartphones allow to measure bias more easily at any time of the day, and especially at moments when it may be relevant for food consumption. This allows us to examine the temporal and situational variability of approach bias. Combining it with repeated delivery of eating-related questions throughout the day (i.e., ecological momentary assessment, EMA) also allows for correlating fluctuations in approach bias with other temporally variable phenomena like food craving, affect and intake.

Several studies have delivered interventions using computer tasks through the internet [52,53,55–57] and have generally reported good compliance rates and effects on dietary intake.

For peer review only

Smartphone-based interventions using similar RT tasks are much rarer and have reported mixed results on key outcomes [49–51,58]. One of the two studies delivering smartphone-based AAI required participants to tilt the phone to respond, and found positive effects on food choice and approach bias towards unhealthy foods [50]. The other study found neither day-level nor longer-term effects of AAI using swipe movements, as compared to an EMA-only intervention and a sham training group [49]. It is important to note that that study did not find any approach bias in participants to begin with which suggests that the swipe movements did not clearly represent approach and avoidance and that the sample size was small. It is therefore unclear to what degree its results can be taken as evidence against the effectiveness of mobile AAI.

One recent AAT variant does not require swiping movements on the touchscreen, but instead requires participants to physically move the phone towards or away from themselves while viewing food stimuli [27,59–61]. This task has been shown to be a valid tool to measure food approach biases outside the laboratory and to provide relevant information beyond self-report measures [59]. In addition to RTs, it also yields data on the force of the movements, which might contain relevant information not captured by RTs [61].

The study presented here sets out to test its effectiveness as an intervention tool for AAI; that is, when it is programmed to pair the foods that a specific participant wants to eat more often with approach, and to pair the foods that a specific participant wants to eat less often with avoidance responses. Specifically, we will study to what degree the intervention can support participants in their goal of changing their eating behavior. We further examine the reliability and validity of approach bias scores obtained through a phone-delivered AAT. Combining the AAI/AAT with the repeated measurement of related phenomena through EMA allows us to disentangle short- and long-term intervention effects as well as to investigate whether approach bias covaries with intake-related variables over time.

METHODS AND ANALYSIS

Study Overview

The study uses a two-arm, double-blind randomized controlled trial conducted with German-speaking participants, and is coordinated at the University of Salzburg, Austria. It compares an active Approach-Avoidance Intervention (AAI) to a sham-training (a measurement Approach-Avoidance Task, AAT) in its impact on eating behavior, food liking, food craving, and food approach bias.

Participants

Participants will be recruited via university e-mailing lists, social networks, university events, and word of mouth. Participants must be between 18 and 60 years of age, and must not be pregnant or report a diagnosis for an eating disorder. Importantly, participants must have an intention to change their eating behavior (which they indicate upon sign-up), without further specification regarding increased or decreased intake of certain foods or food categories.

To determine the required sample size, we performed a power analysis using pre-existing data from a (so far unpublished) study, wherein we attempted to change participants' approach-avoidance bias and analysed its change from pre- to post-training. We opted to use this pre-existing data as the structure of the study is similar to the current study and the correlations and noise therein would be more likely to reflect the findings we will observe than would fully simulated data.

First, group-level differences between the pre-to-post effects of sham and active training were removed such that a time-by-group effect size of 0 was achieved. A new effect size was then

For peer review only

applied by increasing the post-treatment group mean of the active training participants by a multiple of 16 between 64 and 244, giving effect sizes around g = .5. After this, participants were randomly sampled with replacement such that sample sizes between 80 and 180 in multiples of 10 were achieved. Each combination of sample size and effect size was re-sampled and tested 200 times. After sampling a set of participants, a multilevel analysis was performed where approach-avoidance bias scores were predicted with fixed predictors of treatment group (sham or active) and time (pre-training or post-training), as well as random effects of time grouped by participant and time grouped by stimulus. The p-value of the group by time interaction was recorded. The proportion of p-values below .05 was computed to determine the power at each combination of sample size and effect size.

Based on this power analysis, we determined that a medium effect size (g = .50) and a power of .80 would require a sample size of about valid 150 participants. Based on the effect size observed in that other study of g = 0.56, 150 participants result in a power of about .88. A table with all power analysis outcomes is depicted in Supplementary Table 1. With an estimated recruitment rate of three participants per week and allowing for recruitment difficulties slowing down the process, we expect data collection to last from November 2022 to roughly January 2024. Data collection continues until 150 participants are reached.

Materials and procedure

Baseline questionnaires. In a web-based questionnaire (see supplementary file 1), participants give informed consent (consent form see supplementary file 2) and then indicate their age, gender, nationality, state of employment, highest achieved formal education level, dietary restrictions (vegan, vegetarian, pescetarian, omnivorous, other), height, weight, and possible food allergies or intolerances. Participants identifying as female or diverse are also asked about their menstrual cycle. Further, to assess exclusion criteria, they are asked if they are currently suffering from an eating disorder. This is followed by the stimulus selection (see below for details). Participants then complete the German versions of the following questionnaires: subscales restrained eating and external eating from the Dutch Eating Behavior Questionnaire [62]; the Salzburg Emotional Eating Scale [63]; the Salzburg Stress Eating Scale [64]; Perceived Self-Regulatory Success in Dieting [65]; the short version of the UPPS Impulsivity Scale [66].

Stimuli. We pre-selected 90 food and drink pictures from the food.pics [67] and CROCUFID [68] databases, and from freely available online resources based on typical availability in Austria and Germany as the main recruitment sites. At the beginning of the study, participants rated these 90 images on two scales: “In the last three weeks, on how many days have you eaten/drunk this food/drink?” (recent intake) and “In the next three weeks, on how many days would you like to eat/drink this food/drink?” (intended intake). We select the six foods with the most negative difference between past and intended consumption as “increase foods” (eaten less often than intended). Then, among the foods that were eaten at least on six days in the past three weeks, we select six with the most positive difference between past and intended future consumption (eaten more often than intended = “decrease-foods”). A randomly selected four of these six images are then used in the intervention phase in both groups while the other two were left untrained to test whether the intervention would be specific to the foods used in the task. A random selection of 8 out of a set of 12 images of office items serves as control stimuli. Figure 1 displays the selection of stimuli. The food stimuli were not categorized as “healthy” or “unhealthy”, giving participants full flexibility for choosing “increase-foods” and “decrease-foods”.

Setup call. Within a few days of filling out the online questionnaire, a member of the study team contacts participants to schedule a setup call via phone or a video conferencing tool. In this

For peer review only

call, the member of the research team explains the procedure of the study. They further help participants install the necessary apps onto their smartphone (m-path; KU Leuven, 2022 for EMA and the AAT app [1]) and then confirm the correct selection of approach and avoidance foods as determined by the rating task. After the call, participants receive a manual for the study via e-mail, which summarizes the study procedures and the use of the smartphone applications and includes participants' individual three-digit identification code as well as contact information of the study team.

Approach-Avoidance Task (AAT). We use the smartphone-based AAT as introduced by Zech and colleagues [61]. In this version of the task, participants see stimuli on their horizontally held phone screen and they perform approach or avoidance movements by physically moving the phone towards/away from themselves (see Figure 2 and two short introductory videos here: https://osf.io/4k3q9/?view_only=4db6431fd5ee4148a97f3be7f799ea4a). Each trial starts with a fixation dot in the middle of a white screen, which is followed by either one of the food or object stimuli after a 1500 ms delay. While correct approach or avoidance responses make the picture disappear and trigger the start of a new trial, incorrect responses are followed by a 2000 ms display of a black error-cross. If a participant does not respond for 2000 ms, a clock icon is displayed indicating timeout.

The active and sham AAT trainings feature 4 out of 6 approach-foods, 4 out of 6 avoid-foods, as well as 8 out of 12 control object stimuli. The training sessions consist of four training blocks of 16 trials each, and each training block is preceded by 4 practice trials, yielding a training session of 64 training trials and 16 practice trials, or 80 trials total. The pre-, post- and follow-up bias assessment AATs similarly consist of four blocks preceded each by four practice trials but feature all selected images (6 "increase-foods", 6 "decrease-foods" and 12 objects). All 24 images are presented one time per block, yielding 96 test trials and 16 practice trials, or 112 trials total. In all AATs, the instructions of the blocks alternate such that participants are instructed to approach foods while avoiding objects in the first block (approach-food blocks) and avoid foods while approaching objects in the second block (avoid-food blocks). This order is the same for all sessions and all participants. Crucially, in the active training AATs, only approach-foods are shown in the approach-food blocks, while only avoid-foods are shown in the avoid-food blocks; sham training instead features both approach- and avoid-foods during both approach-food and avoid-food blocks. Completing one session of the AAT/AAI takes about five minutes.

only

[1] The app can be downloaded on Android devices from this address: https://play.google.com/store/apps/details?id=com.eatlabsbg.eatapp

For peer review only - http://bmjopen.bmj.com/site/about/guidelines.xhtml

Table 1: overview of EMA questions (own translations from German). All items are answered on a slider from 0 to 100 unless indicated differently in parentheses. Midday prompts remain the same across the entire study duration. Evening prompts differ depending on the study phase as indicated:

* These items are asked alongside a food image and are repeated for each image (six “increase-food” and six “decrease-food” images).

[+] This item is only asked after the first and the last AAT session (day 5 and 15).

**Item**	**Midday (all study phases)**	**Evening Days 1-4 and 17-20**	**Evening Days 5-16**	**Evening - follow-up**
When was the last time you ate something?	X			
What type of meal was it? (breakfast, lunch, dinner, snack)	X			
How hungry are you right now?	X	X	X	
Do you feel like you have everything under control?	X			
Do you feel like you are on top of things?	X			
How optimistic do you feel right now?	X			X
How happy do you feel right now?	X			X
How lonely do you feel right now?	X			X
How depressed do you feel right now?	X			X
How angry do you feel right now?	X			X
How mad do you feel right now?	X			X
How tense do you feel right now?	X			X
How anxious do you feel right now?	X			X
How much do you want to stick to your dietary goals *for the rest of the day*?	X			
How much do you want to stick to your dietary goals *tomorrow*?		X		X
How strong has your craving for this food been *today*? *		X		X
How much of this food have you eaten *today*? *		X		X
Have you said things today without thinking?	X	X	X	X

For peer review only

Have you spent more money today than you wanted to?	X	X	X	X
Have you felt impatient today?	X	X	X	X
Have you made a spontaneous decision today?	X	X	X	X
How strong has your craving for this food been *since the midday questionnaire*? *			X	
How much of this food have you eaten *since the midday questionnaire*? *			X	
How much do you expect that this task will help you reach your dietary goals? +	X+			
Throughout the whole study, how often did you push this food away from yourself? *				X
Please indicate the day your last period started.				X

For peer review only

Ecological Momentary Assessment (EMA). Participants follow the EMA schedule for a total of 20 days. During the whole period, participants receive two prompts per day (delivered through the smartphone application m-path [69]), one just before the time a participant usually eats lunch and the other in the evening (prompted at an individualized time agreed-upon with the participant to represent an end-of-day signal). Table 1 shows the questions that participants answer on those prompts and Figure 3 displays the temporal sequence of the study. EMA prompts on days 1-3 of the study only contain the listed questions. On day 4 (the day before the start of the intervention) and day 17 (the day after the end of the intervention), participants receive an instruction to open the AAT application and complete a measurement AAT. On every second day during the intervention phase (days 5 through 16), participants receive an instruction to open the AAT application and complete a training AAT after completing the midday prompt. 30 minutes after completing the midday prompt, participants receive a notification asking whether they conducted the training. On replying “yes”, they receive positive feedback; on replying “no”, they are asked to now open the AAT app to conduct the task. The number of sessions was chosen based on earlier, similar studies [50,52], balancing participant burden, compliance, and intervention intensity.

In addition, on day 6 and day 16 (the first and the last day including an AAT/AAI session), participants further indicate their expectancy of how much the task will help them reach their dietary goals. Four weeks after the end of the initial 20-day EMA period, participants receive one additional EMA questionnaire and a measurement AAT in the evening. After performing this final AAT, participants indicate how often they believed they pushed or pulled each of their “decrease-foods” and “increase-foods”.

Procedure. The procedure for study participation is as follows: after interested participants contact the study team, they receive an individual participant code and a weblink to the baseline questionnaire. At this point, an R-script [2] randomizes participants to either the intervention or control group with the condition unknown to the study team. After a setup call with a member of the research team within a few days of filling out the questionnaires, participants start receiving EMA prompts and AAT as described above. Figure 3 shows the timeline of the whole study. Throughout the study period, participants can contact study personnel who also monitor compliance to the EMA and AAT schedule and contact participants in case of low compliance: participants receive an e-mail if they miss more than one of the first three AAT/AAI sessions.

Outcomes

Main outcomes. This study uses three main outcome measures. The first outcome measure is participants’ self-reported intake of “increase” and “decrease” foods according to the EMA schedule outlined above, on a slider from 0 (labelled “nothing”) to 100 (“very much”). The second outcome measure is participants’ self-reported craving for those same foods in the same manner.

The third outcome measure is the approach bias for all selected foods based on the RT and force in the AAT. The RT is defined as the time from picture onset to movement onset. Force is defined as the peak acceleration in the correct direction during a trial, standardized within participant by dividing every individual’s measurement of force by the participant-specific standard deviation. Separately for approach and avoidance trials as well as for sessions, the RT and the force will be averaged across the four AAT blocks for each specific food stimulus. For objects, we will also average across the different stimuli. The average approach or avoid response for objects on a

[2] The function *sample* randomly outputs the number “1” or “2” which correspond to the conditions.

For peer review only - http://bmjopen.bmj.com/site/about/guidelines.xhtml

session will be subtracted from stimulus-specific food approach or avoidance response on that session to achieve food-specific *single-difference* approach and avoidance scores according to these formulas:

Stimulus-specific approach = [food-specific approach]-[average object approach]

Stimulus-specific avoidance = [food-specific avoidance]-[average object avoidance]

Double-difference scores will be used as a full bias score per food stimulus and session, according to the formula: ([food-specific avoidance]-[food-specific approach])-([average object avoidance]-[average object approach]).

Secondary outcomes. Dietary intentions are measured according to the outlined EMA schedule.

Data analysis plan

Data analysis will serve to investigate a series of research questions relating to different aspects of the study. In this section we will provide a brief description. Full details on the data analysis, including the exact multilevel analysis formulas, are available in the pre-registration at https://osf.io/yn7kt .

Data exclusion. For analyses regarding the effectiveness of the AAI, we exclude participants who did not conduct any of the AATs during the intervention phase, as we regard them as "not treated". For the remaining participants, sensitivity analyses are performed to test whether the number of completed training sessions affects intervention effectiveness. For analyses of approach bias, we exclude error trials and trials with RTs that deviate more than +/-3SD from the individual mean of the participant in that AAT session. If more than 25% of trials must be excluded based on these criteria, the whole AAT session is excluded from further analysis. This post-hoc session exclusion does not affect whether a participant is counted as "not treated" or not in the analyses regarding the effectiveness of the AAI.

Overall intervention effectiveness. The first set of research questions relates to the effectiveness of the intervention as compared to the control condition from pre- to post-training. To this end, we use multilevel models to predict intake of trained "increase" and "decrease" foods as a function of timepoint (three days pre- vs post-intervention), condition (intervention vs control), and their interaction. Equivalent models test the intervention effect on approach biases towards - and craving for - the two food categories. To test to what degree the effect of the training intervention is specific to trained foods, we then use data from trained and untrained stimuli and add a variable that indicates whether a food appeared in the training or not (trained vs untrained) and all interaction terms to the model. This is followed up with tests to determine whether changes in the approach bias are mainly driven by changes in approach- or avoidance RTs. We further test the moderating role of intentions, baseline stimulus craving, and person-level variables obtained from the questionnaires, as well as contingency awareness and expectancy by adding the relevant variable and its interaction terms to the equations. Finally, we examine the mediating effect of craving for changes in intake.

Immediate intervention effectiveness. The second set of research questions concerns the short-term effects of the intervention during the intervention phase (days 5-16). Multilevel models predicting food intake and cravings, respectively, include the factors group (intervention vs control) and (off-)training day (training day vs no training day) as predictors. In another pair of multilevel models, we use group and the number of days since the beginning of the intervention and their

For peer review only

interaction as predictors of craving and food intake, respectively. The force applied during the training is used as a predictor for the change in craving and intake from before the start of the training.

Trait and state components of approach bias. The third set of research questions relates to the state and trait components of approach bias and is examined within the control group only. This is because only participants in the control group receive measurement AATs throughout the study period. Multilevel models test whether bias size and negative emotions are related on both a between-subjects and a within-subjects level and to what degree this depends on the strength of the desire for these foods. A separate model tests equivalent research questions for the relation between bias strength and craving, as well as bias strength and intake, respectively. The latter analyses testing how bias strength is related to subsequent food intake are expanded by including trait and day-level impulsivity and day-level intentions of regulating food intake.

Patient and public involvement

Patients and the public are not involved in study design, data collection, data analysis, or dissemination.

Ethics, dissemination, and data handling.

The study has received ethical approval from the ethics board of the University of Salzburg and is conducted in accordance with the declaration of Helsinki. Results of the trial will be disseminated through a series of articles in appropriate scientific journals and conference presentations.

Data is handled confidentially and stored in a pseudonymized manner. Neither m-path nor the AAT application collect personal data but work through three-digit identification codes assigned to participants. The identification key linking personal data to the identification codes will be kept in password-protected files separately from the pseudonymized data and will be destroyed one year after termination of the study. Deidentified data will be archived for at least ten years and consent forms as documentation of participation will be archived for 30 years. The deidentified data will be made public on the Open Science Framework after the completion of planned publications.

For peer review only

Contributorship statement

MBA: conceptualisation, methodology, writing first draft of the protocol, revising the protocol. HvA: conceptualisation, methodology, analysis plan, reviewing drafts of the protocol. SK: conceptualisation, methodology, analysis plan, reviewing drafts of the protocol. JB: conceptualisation, methodology, supervision, reviewing drafts of the protocol. All authors read and approved the final version of the protocol.

Competing interests

All authors report no competing interests.

Funding

This work was supported by the Austrian Science Fund FWF grant number P 34542-B. Sercan Kahveci and Hannah van Alebeek were supported by the Doctoral College "Imaging the Mind" (FWF; W1233-B). Hannah van Alebeek was additionally supported by the project: Mapping neural mechanisms of appetitive behaviour (FWF; KLI762-B). The funder plays no role in the study design; collection, management, analysis, and interpretation of data; writing of the report; and the decision to submit the report for publication.

References

1 Wansink B, Sobal J. Mindless Eating: The 200 Daily Food Decisions We Overlook. *Environ Behav* 2007;**39**:106–23. doi:10.1177/0013916506295573

2 World Health Organization. Obesity and Overweight. Published Online First: 2018. doi:10.1007/978-3-319-33228-4_447

3 Sheeran P, Webb TL. The Intention–Behavior Gap. *Soc Personal Psychol Compass* 2016;**10**:503–18. doi:10.1111/spc3.12265

4 Gawronski B, De Houwer J. Implicit measures in social and personality psychology. In: *Handbook of research methods in social and personality psychology*. Cambridge University Press 2014. 283–310.http://hdl.handle.net/1854/LU-2959505 (accessed 29 Apr 2020).

5 Gawronski B, Hahn A. IMPLICIT MEASURES Procedures, Use, and Interpretation. In: Blanton H, LaCroix JM, Webster GD, eds. *Measurement in Social Psychology*. New York, NY: : Yaylor & Francis 2019. 29–55.http://www.bertramgawronski.com/documents/GH2019Measurement.pdf

6 Hagger MS, Trost N, Keech J, *et al.* Predicting sugar consumption: Application of an integrated dual-process, dual-phase model. *Appetite* Published Online First: 2017. doi:10.1016/j.appet.2017.04.032

7 Hofmann W, Friese M, Wiers RW. Impulsive versus reflective influences on health behavior: a theoretical framework and empirical review. *Health Psychol Rev* 2008;**2**:111–37. doi:10.1080/17437190802617668

8 Van Dessel P, Cummins J, Hughes S, *et al.* Reflecting on 25 Years of Research Using Implicit Measures: Recommendations for Their Future Use. *Soc Cogn* 2020;**38**:s223–42. doi:10.1521/soco.2020.38.supp.s223

For peer review only

9 Kahveci S, van Alebeek H, Berking M, *et al.* Touchscreen-based assessment of food approach biases: investigating reliability and item-specific preferences. *Appetite* 2021;:105190. doi:10.1016/j.appet.2021.105190

10 Meule A, Lender A, Richard A, *et al.* Approach–avoidance tendencies towards food: Measurement on a touchscreen and the role of attention and food craving. *Appetite* 2019;**137**:145–51.

11 Rinck M, Becker ES. Approach and avoidance in fear of spiders. *J Behav Ther Exp Psychiatry* 2007;**38**:105–20. doi:10.1016/j.jbtep.2006.10.001

12 Schimmack U. The Implicit Association Test: A Method in Search of a Construct. *Perspect Psychol Sci* 2019;:1745691619863798. doi:10.1177/1745691619863798

13 De Houwer J. Implicit Bias Is Behavior: A Functional-Cognitive Perspective on Implicit Bias. *Perspect Psychol Sci* 2019;:1745691619855638. doi:10.1177/1745691619855638

14 Brockmeyer T, Hahn C, Reetz C, *et al.* Approach bias and cue reactivity towards food in people with high versus low levels of food craving. *Appetite* 2015;**95**:197–202. doi:10.1016/j.appet.2015.07.013

15 Kakoschke N, Kemps E, Tiggemann M. Impulsivity moderates the effect of approach bias modification on healthy food consumption. *Appetite* 2017;**117**:117–25. doi:10.1016/j.appet.2017.06.019

16 Kakoschke N, Kemps E, Tiggemann M. Differential effects of approach bias and eating style on unhealthy food consumption in overweight and normal weight women. *Psychol Health* 2017;**32**:1371–85. doi:10.1080/08870446.2017.1327587

17 Spruyt A, De Houwer J, Tibboel H, *et al.* On the predictive validity of automatically activated approach/avoidance tendencies in abstaining alcohol-dependent patients. *Drug Alcohol Depend* 2013;**127**:81–6. doi:10.1016/j.drugalcdep.2012.06.019

18 Friese M, Hofmann W, Schmitt M. When and why do implicit measures predict behaviour? Empirical evidence for the moderating role of opportunity, motivation, and process reliance. *Eur Rev Soc Psychol* 2008;**19**:285–338. doi:10.1080/10463280802556958

19 Kakoschke N, Albertella L, Lee RSC, *et al.* Assessment of Automatically Activated Approach–Avoidance Biases Across Appetitive Substances. *Curr Addict Rep* 2019;:1–10. doi:10.1007/s40429-019-00254-2

20 Kemps E, Tiggemann M. Approach Bias for Food Cues in Obese Individuals. *Psychol Health* 2014;**0446**:1–25. doi:10.1080/08870446.2014.974605

21 Neimeijer RAM, de Jong PJ, Roefs A. Automatic approach/avoidance tendencies towards food and the course of anorexia nervosa. *Appetite* 2015;**91**:28–34. doi:http://dx.doi.org/10.1016/j.appet.2015.03.018

22 Kahveci S, Meule A, Lender A, *et al.* Food approach bias is moderated by the desire to eat specific foods. *Appetite* 2020;**154**:104758. doi:10.1016/j.appet.2020.104758

23 Kemps E, Tiggemann M, Martin R, *et al.* Implicit approach-avoidance associations for craved food cues. *J Exp Psychol Appl* 2013;**19**:30–8. doi:10.1037/a0031626

For peer review only

24 Lender A, Meule A, Rinck M, *et al.* Measurement of food-related approach–avoidance biases: Larger biases when food stimuli are task relevant. *Appetite* 2018;**125**:42–7. doi:10.1016/j.appet.2018.01.032

25 Wittekind CE, Blechert J, Schiebel T, *et al.* Comparison of different response devices to assess behavioral tendencies towards chocolate in the approach-avoidance task. *Appetite* 2021;**165**:105294. doi:10.1016/j.appet.2021.105294

26 Weingarten HP, Elston D. The phenomenology of food cravings. *Appetite* 1990;**15**:231–46.

27 Zech HG, Gable P, van Dijk WW, *et al.* Test-retest reliability of a smartphone-based approach-avoidance task: Effects of retest period, stimulus type, and demographics. *Behav Res Methods* Published Online First: 1 August 2022. doi:10.3758/s13428-022-01920-6

28 Gawronski B, Morrison M, Phills CE, *et al.* Temporal Stability of Implicit and Explicit Measures: A Longitudinal Analysis. *Pers Soc Psychol Bull* 2017;**43**:300–12. doi:10.1177/0146167216684131

29 Krehbiel J, Halbeisen G, Kühn S, *et al.* Too hot to handle: Mood states moderate implicit approach vs. avoidance tendencies toward food cues in patients with obesity and active binge eating disorder. *J Psychiatr Res* 2021;**143**:302–8. doi:10.1016/j.jpsychires.2021.09.031

30 Neimeijer RAM, Roefs A, Ostafin BD, *et al.* Automatic Approach Tendencies toward High and Low Caloric Food in Restrained Eaters: Influence of Task-Relevance and Mood. *Front Psychol* 2017;**8**.https://www.frontiersin.org/articles/10.3389/fpsyg.2017.00525 (accessed 24 Oct 2022).

31 Richard A, Meule A, Blechert J. When and how do explicit measures of food craving predict implicit food evaluation? A moderated mediation model. *Food Qual Prefer* 2018;**66**:141–7. doi:10.1016/J.FOODQUAL.2018.01.018

32 Richard A, Meule A, Blechert J. Implicit evaluation of chocolate and motivational need states interact in predicting chocolate intake in everyday life. *Eat Behav* 2019;**33**:1–6. doi:10.1016/j.eatbeh.2019.01.006

33 Zech HG. Food approach dynamics in daily live: Speed and force of food approach movements fluctuate with hunger, but less so for people with high BMI. 2022.

34 Veling H, Holland RW, van Knippenberg A. When approach motivation and behavioral inhibition collide: Behavior regulation through stimulus devaluation. *J Exp Soc Psychol* 2008;**44**:1013–9. doi:10.1016/j.jesp.2008.03.004

35 Veling H, Becker D, Liu H, *et al.* How go/no-go training changes behavior: A value-based decision-making perspective. *Curr Opin Behav Sci* 2022;**47**:101206. doi:10.1016/j.cobeha.2022.101206

36 Houben K, Aulbach M. Is there a difference between stopping and avoiding? A review of the mechanisms underlying Go/No-Go and Approach-Avoidance training for food choice. *Curr Opin Behav Sci* 2023;**49**.

37 Van Dessel P, Gawronski B, Smith CT, *et al.* Mechanisms underlying approach-avoidance instruction effects on implicit evaluation: Results of a preregistered adversarial collaboration. *J Exp Soc Psychol* 2017;**69**:23–32. doi:10.1016/J.JESP.2016.10.004

For peer review only

38 Van Dessel P, Hughes S, De Houwer J. How Do Actions Influence Attitudes? An Inferential Account of the Impact of Action Performance on Stimulus Evaluation. *Personal Soc Psychol Rev* 2019;**23**:267–84. doi:10/gf5sps

39 Dickson H, Kavanagh DJDJ, MacLeod C. The pulling power of chocolate: Effects of approach-avoidance training on approach bias and consumption. *Appetite* 2016;**99**:46–51. doi:10.1016/j.appet.2015.12.026

40 Veling H, Verpaalen IAM, Liu H, *et al.* How can food choice best be trained? Approach-avoidance versus go/no-go training. *Appetite* 2021;:105226. doi:10/gjkp4t

41 Schumacher SE, Kemps E, Tiggemann M. Bias modification training can alter approach bias and chocolate consumption. *Appetite* 2016;**96**:219–24. doi:10.1016/j.appet.2015.09.014

42 Aulbach MB, Knittle K, Haukkala A. Implicit process interventions in eating behaviour: a meta-analysis examining mediators and moderators. *Health Psychol Rev* 2019;**13**:179–208. doi:10.1080/17437199.2019.1571933

43 Becker D, Jostmann NB, Wiers RW, *et al.* Approach avoidance training in the eating domain: Testing the effectiveness across three single session studies. *Appetite* 2015;**85**:58–65. doi:10.1016/j.appet.2014.11.017

44 Becker D, Jostmann NB, Holland RW. *Does approach bias modification really work in the eating domain? A commentary on Kakoschke et al. (2017)*. 2018. doi:10.1016/j.addbeh.2017.02.025

45 Navas JF, Verdejo-García A, Vadillo MA. The evidential value of research on cognitive training to change food-related biases and unhealthy eating behavior: A systematic review and p-curve analysis. *Obes Rev* 2021;**22**:e13338. doi:10.1111/obr.13338

46 Yang Y, Shields GS, Wu Q, *et al.* Cognitive training on eating behaviour and weight loss: A meta-analysis and systematic review. *Obes Rev* 2019;**0**. doi:10.1111/obr.12916

47 Van Dessel P, De Houwer J, Gast A. Approach–Avoidance Training Effects Are Moderated by Awareness of Stimulus–Action Contingencies. *Pers Soc Psychol Bull* 2016;**42**:81–93. doi:10.1177/0146167215615335

48 Wiers RW, Van Dessel P, Köpetz C. ABC Training: A New Theory-Based Form of Cognitive-Bias Modification to Foster Automatization of Alternative Choices in the Treatment of Addiction and Related Disorders. *Curr Dir Psychol Sci* 2020;**29**:499–505. doi:10/ghfdf3

49 Meule A, Richard A, Dinic R, *et al.* Effects of a Smartphone-Based Approach-Avoidance Intervention on Chocolate Craving and Consumption: Randomized Controlled Trial. *JMIR MHealth UHealth* 2019;**7**:e12298. doi:10.2196/12298

50 Kakoschke N, Hawker C, Castine B, *et al.* Smartphone-based cognitive bias modification training improves healthy food choice in obesity: A pilot study. *Eur Eat Disord Rev* 2018;**26**:526–32. doi:10.1002/erv.2622

51 Aulbach MB, Knittle K, van Beurden SB, *et al.* App-based food Go/No-Go training: User engagement and dietary intake in an opportunistic observational study. *Appetite* 2021;**165**:105315. doi:10/gmvjtq

For peer review only

52 Lawrence NS, O'Sullivan J, Parslow D, *et al.* Training response inhibition to food is associated with weight loss and reduced energy intake. *Appetite* 2015;**95**:17–28. doi:10.1016/j.appet.2015.06.009

53 Stice E, Yokum S, Veling H, *et al.* Pilot test of a novel food response and attention training treatment for obesity: Brain imaging data suggest actions shape valuation. *Behav Res Ther* 2017;**94**:60–70. doi:10.1016/j.brat.2017.04.007

54 Stice E, Yokum S, Gau J, *et al.* Efficacy of a food response and attention training treatment for obesity: A randomized placebo controlled trial. *Behav Res Ther* 2022;**158**:104183. doi:10.1016/j.brat.2022.104183

55 Forman EM, Manasse SM, Dallal DH, *et al.* Computerized neurocognitive training for improving dietary health and facilitating weight loss. *J Behav Med* Published Online First: 2019. doi:10.1007/s10865-019-00024-5

56 Poppelaars A, Scholten H, Granic I, *et al.* When winning is losing: A randomized controlled trial testing a video game to train food-specific inhibitory control. *Appetite* 2018;**129**:143–54. doi:10.1016/j.appet.2018.06.039

57 Veling H, van Koningsbruggen GMGM, Aarts H, *et al.* Targeting impulsive processes of eating behavior via the internet. Effects on body weight. *Appetite* 2014;**78**:102–9. doi:10.1016/j.appet.2014.03.014

58 Keeler JL, Chami R, Cardi V, *et al.* App-based food-specific inhibitory control training as an adjunct to treatment as usual in binge-type eating disorders: A feasibility trial. *Appetite* 2022;**168**:105788.

59 Brouwer A-M, van Beers JJ, Sabu P, *et al.* Measuring Implicit Approach–Avoidance Tendencies towards Food Using a Mobile Phone outside the Lab. *Foods* 2021;**10**:1440. doi:10.3390/foods10071440

60 van Beers JJ, Kaneko D, Stuldreher IV, *et al.* An Accessible Tool to Measure Implicit Approach-Avoidance Tendencies Towards Food Outside the Lab. In: *Companion Publication of the 2020 International Conference on Multimodal Interaction*. Virtual Event Netherlands: : ACM 2020. 307–11. doi:10.1145/3395035.3425647

61 Zech HG, Rotteveel M, van Dijk WW, *et al.* A mobile approach-avoidance task. *Behav Res Methods* 2020;**52**:2085–97. doi:10.3758/s13428-020-01379-3

62 van Strien T, Frijters JER, Bergers GPA, *et al.* The Dutch Eating Behavior Questionnaire (DEBQ) for assessment of restrained, emotional, and external eating behavior. *Int J Eat Disord* 1986;**5**:295–315. doi:10.1002/1098-108X(198602)5:2<295::AID-EAT2260050209>3.0.CO;2-T

63 Meule A, Reichenberger J, Blechert J. Development and Preliminary Validation of the Salzburg Emotional Eating Scale. *Front Psychol* 2018;**9**.https://www.frontiersin.org/articles/10.3389/fpsyg.2018.00088 (accessed 18 Oct 2022).

64 Meule A, Reichenberger J, Blechert J. Development and preliminary validation of the Salzburg Stress Eating Scale. *Appetite* 2018;**120**:442–8. doi:10.1016/j.appet.2017.10.003

For peer review only

65 Meule A, Papies EK, Kübler A. Differentiating between successful and unsuccessful dieters. Validity and reliability of the Perceived Self-Regulatory Success in Dieting Scale. *Appetite* 2012;**58**:822–6. doi:10.1016/j.appet.2012.01.028

66 Whiteside SP, Lynam DR, Miller JD, *et al.* Validation of the UPPS impulsive behaviour scale: a four-factor model of impulsivity. *Eur J Personal* 2005;**19**:559–74.

67 Blechert J, Lender A, Polk S, *et al.* Food-Pics_Extended—An Image Database for Experimental Research on Eating and Appetite: Additional Images, Normative Ratings and an Updated Review. *Front Psychol* 2019;**10**:307. doi:10.3389/fpsyg.2019.00307

68 Toet A, Kaneko D, de Kruijf I, *et al.* CROCUFID: A Cross-Cultural Food Image Database for Research on Food Elicited Affective Responses. *Front Psychol* 2019;**10**:58. doi:10.3389/fpsyg.2019.00058

69 KU Leuven. m-Path – Free tool for blended care by KU Leuven. 2022.https://m-path.io/landing/ (accessed 18 Oct 2022).

For peer review only

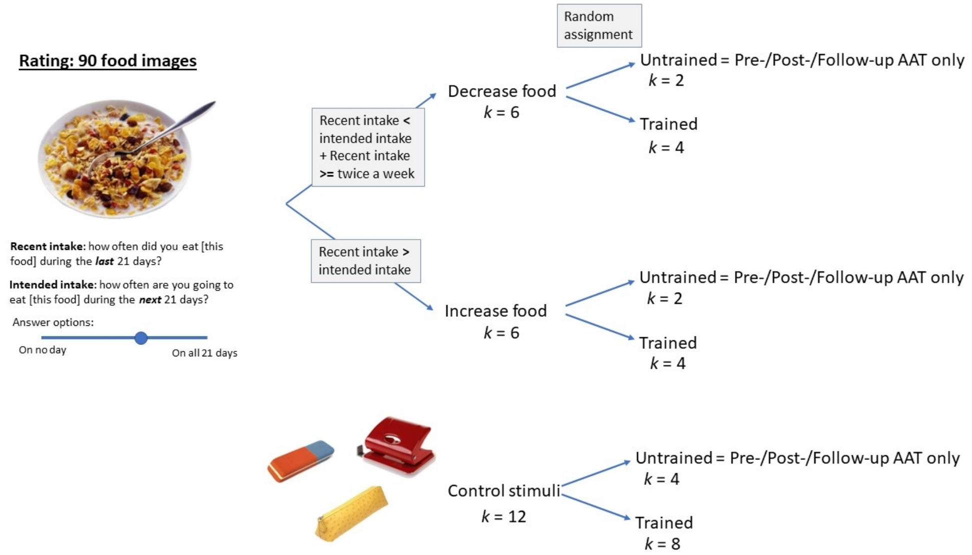

Figure 1: selection of the images presented in the training.

338x190mm (96 x 96 DPI)

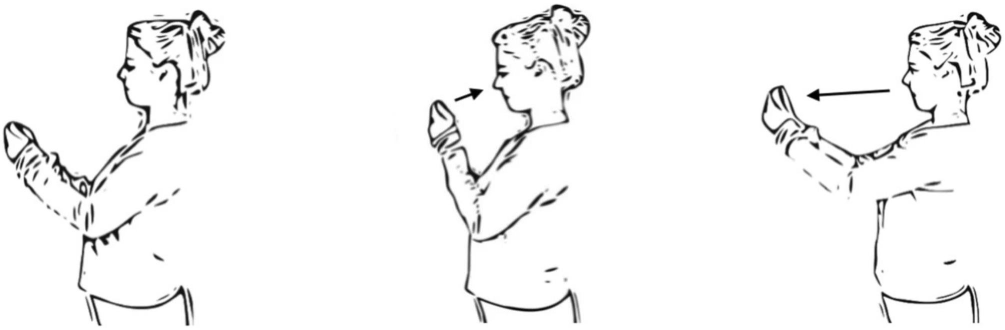

Figure 2 (reproduced from Zech et al., 2020): movements in the mobile Approach-Avoidance task. The image on the left shows the starting position, the image in the middle an approach trial and the image on the right an avoidance trial

274x89mm (120 x 120 DPI)

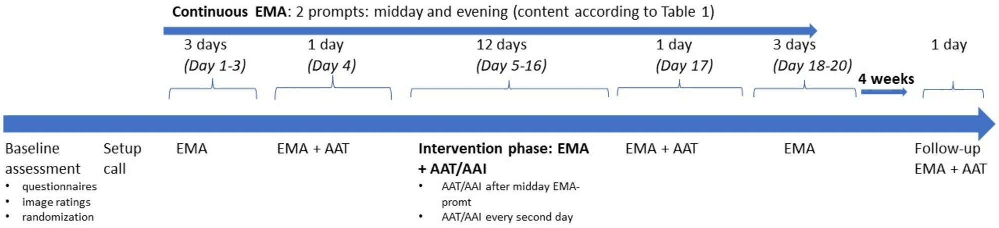

Figure 3: time schedule of the whole study period.

338x110mm (96 x 96 DPI)

Supplementary Table 1: Statistical power for a range of effect sizes and sample sizes.

Sample size	*Effect size (Hedges' g)*										
	.28	*.36*	*.43*	*.5*	*.57*	*.65*	*.72*	*.79*	*.86*	*.93*	*1.01*
80	.19	.28	.38	.52	.64	.72	.82	.90	.94	.98	.98
90	.24	.34	.43	.58	.70	.76	.89	.94	.96	.96	.99
100	.17	.32	.46	.65	.69	.83	.86	.94	.98	.98	1
110	.24	.42	.57	.66	.82	.85	.92	.97	.99	1	1
120	.23	.41	.62	.66	.81	.89	.94	.97	1	1	.99
130	.34	.36	.62	.78	.86	.90	.97	.99	.99	1	.99
140	.32	.49	.56	.77	.88	.97	.97	.99	1	.99	1
150	.36	.52	.63	.78	.88	.94	.97	1	1	1	1
160	.35	.57	.68	.82	.90	.96	.98	.99	.99	1	1
170	.41	.54	.72	.83	.92	.97	.98	.99	1	1	1
180	.37	.54	.70	.90	.92	.98	.99	.99	1	1	1

For peer review only

Teil A: Zahlencode

A1. **Bitte geben Sie hier Ihren Zahlencode aus der Mail ein:**

Teil B: Demografische Variablen

Bitte beantworten Sie zunächst einige Fragen zu Ihrer Person.

Leider keine Teilnahme möglich

Eine der Apps, die für die Studie benötigt wird, funktioniert momentan nur mit Android-Betriebssystemen. Deshalb können Sie leider ohne ein entsprechendes Smartphone nicht an der Studie teilnehmen. Falls Sie an einer anderen psychologischen Studie teilnehmen wollen, finden Sie eine Studienliste unserer Abteilung auf dieser Seite: https://sites.google.com/site/eatingandanxietylab/mitmachen

Sie können diesen Fragebogen jetzt schließen.

Leider keine Teilnahme möglich

Leider erfüllen Sie nicht die Einschlusskriterien für diese Studie. Falls Sie an einer anderen psychologischen Studie teilnehmen wollen, finden Sie eine Studienliste unserer Abteilung auf dieser Seite: https://sites.google.com/site/eatingandanxietylab/mitmachen

Sie können diesen Fragebogen jetzt schließen.

Leider keine Teilnahme möglich

Leider erfüllen Sie nicht die Einschlusskriterien für diese Studie. Falls Sie an einer anderen psychologischen Studie teilnehmen wollen, finden Sie eine Studienliste unserer Abteilung auf dieser Seite: https://sites.google.com/site/eatingandanxietylab/mitmachen

Sie können diesen Fragebogen jetzt schließen.

B1. Um an der Studie teilnehmen zu können, benötigen Sie ein Smartphone mit einem Android-Betriebssystem. Bitte geben Sie daher hier die auf Sie passende Antwort an:

- Ich habe ein Smartphone mit Android-Betriebssystem zur Verfügung (ein eigenes, oder für die Dauer der Studie aus meinem Umfeld organisiert). ☐
- Ich habe kein Smartphone mit Android-Betriebssystem, wäre aber bereit, mir für die Studie eines von der Uni Salzburg auszuleihen. ☐
- Ich habe kein Smartphone mit Android-Betriebssystem zur Verfügung und wäre nicht bereit, mir für die Studie eines von der Uni Salzburg auszuleihen. ☐

B2. Bitte geben Sie Ihr Alter an.

B3. **Bitte geben Sie Ihr Geschlecht an.**

- weiblich ☐
- männlich ☐
- divers ☐

B4. **Sind Sie schwanger?**

- Ja ☐
- Nein ☐

B5. **Bitte wählen Sie alle auf Sie zutreffenden Optionen bezüglich Ihres Menstruationszyklus und/oder das von Ihnen aktuell verwendete Verhütungsmittel aus.**

- Trifft nicht zu ☐
- Natürlicher Menstruationszyklus ☐
- Verwendung der Kupferspirale, Kupferkette, Goldspirale ☐
- Verwendung der Hormonspirale ☐
- Verwendung der Pille ☐
- Verwendung der Pille, die IMMER eingenommen wird ☐
- Dreimonatsspritze ☐
- Hormonimplantat ☐
- Verhütungspflaster ☐
- Hormonersatztherapie ☐
- Menopause ☐

For peer review only - http://bmjopen.bmj.com/site/about/guidelines.xhtml

Sonstiges

Sonstiges

B6. **Bitte geben Sie den Starttag Ihrer letzten Menstruation an.**

B7. **Bitte geben Sie den Starttag Ihrer vorletzten Menstruation an.**

B8. **Bitte geben Sie den Starttag Ihrer drittletzten Menstruation an.**

B9. **Bitte tragen Sie Ihre Nationalität ein.**

B10. Was ist Ihr höchster erreichter Bildungsabschluss?

- Ohne Schulabschluss ☐
- Hauptschulabschluss ☐
- abgeschlossene Lehre ☐
- mittlere Reife ☐
- Abitur / Matura ☐
- abgeschlossenes Bachelorstudium ☐
- abgeschlossenes Masterstudium ☐
- Sonstiges ☐

Sonstiges

B11. Welcher Berufsgruppe gehören Sie an?

- Student/in ☐
- Auszubildende/r ☐
- Schüler/in ☐
- Angestellte/r ☐
- Selbstständige/r ☐
- Sonstiges ☐

Sonstiges

B12. Wie ernähren Sie sich?

- Vegan (Ernährung ohne von Tieren stammenden Nahrungsmittel) ☐
- Vegetarisch (fleisch- und fischfreie Ernährung) ☐
- Pescetarisch (fleisch- aber nicht fischfreie Ernährung) ☐
- Omnivor (kein Nahrungsmittelverzicht) ☐
- Sonstiges ☐

Sonstiges

B13. **Bitte geben Sie Ihre aktuelle Körpergröße an.**

Angabe in Zentimetern.

B14. **Bitte geben Sie Ihr aktuelles Gewicht an.**

Angabe in Kilogramm.

B15. **Haben Sie eine Nahrungsmittelunverträglichkeit?**

Ja

Nein

B16. **Welche Nahrungsmittelunverträglichkeit haben Sie?**

B17. **Haben Sie aktuell (d.h. innerhalb der letzten 12 Wochen) eine diagnostizierte Essstörung?**

Ja

Nein

Teil C: Konsumziele 1/9

Auf den nächsten 9 Seiten bekommen Sie Bilder von insgesamt 90 Nahrungsmitteln angezeigt und die Aufforderung, jeweils Ihren vergangenen und zukünftigen Konsum der abgebildeten Nahrungsmittel anzugeben. Die Anpassung der Studie auf Ihre Ernährungsziele erfolgt aufgrund Ihrer Antworten in diesem Teil des Fragebogens. Für eine möglichst gute Individualisierung ist es daher wichtig, dass Sie die Fragen möglichst genau beantworten.

C1. **Erdnussflips**

An wie vielen Tagen haben Sie in den letzten drei Wochen Erdnussflips gegessen?|an keinem Tag|an allen 21 Tagen

An wie vielen Tagen wollen Sie in den nächsten drei Wochen Erdnussflips essen?|an keinem Tag|an allen 21 Tagen

C2. **gemischtes Müsli**

An wie vielen Tagen haben Sie in den letzten drei Wochen gemischtes Müsli gegessen?|an keinem Tag|an allen 21 Tagen

An wie vielen Tagen wollen Sie in den nächsten drei Wochen gemischtes Müsli essen?|an keinem Tag|an allen 21 Tagen

C3. **Nussmischung**

An wie vielen Tagen haben Sie in den letzten drei Wochen Studentenfutter gegessen?|an keinem Tag|an allen 21 Tagen

An wie vielen Tagen wollen Sie in den nächsten drei Wochen Studentenfutter essen?|an keinem Tag|an allen 21 Tagen

C4. **Fisch**

An wie vielen Tagen haben Sie in den letzten drei Wochen Fisch gegessen?|an keinem Tag|an allen 21 Tagen

An wie vielen Tagen wollen Sie in den nächsten drei Wochen Fisch essen?|an keinem Tag|an allen 21 Tagen

C5. **salziger Snackmix**

An wie vielen Tagen haben Sie in den letzten drei Wochen salzigen Snackmix gegessen?|an keinem Tag|an allen 21 Tagen

An wie vielen Tagen wollen Sie in den nächsten drei Wochen salzigen Snackmix essen?|an keinem Tag|an allen 21 Tagen

C6. **Pizza**

An wie vielen Tagen haben Sie in den letzten drei Wochen Pizza gegessen?|an keinem Tag|an allen 21 Tagen

An wie vielen Tagen wollen Sie in den nächsten drei Wochen Pizza essen?|an keinem Tag|an allen 21 Tagen

C7. Honig

An wie vielen Tagen haben Sie in den letzten drei Wochen Honig gegessen?|an keinem Tag|an allen 21 Tagen

An wie vielen Tagen wollen Sie in den nächsten drei Wochen Honig essen?|an keinem Tag|an allen 21 Tagen

C8. Chicken Nuggets

An wie vielen Tagen haben Sie in den letzten drei Wochen Chicken Nuggets gegessen?|an keinem Tag|an allen 21 Tagen

An wie vielen Tagen wollen Sie in den nächsten drei Wochen Chicken Nuggets essen?|an keinem Tag|an allen 21 Tagen

C9. Tofu

An wie vielen Tagen haben Sie in den letzten drei Wochen Tofu gegessen?|an keinem Tag|an allen 21 Tagen

An wie vielen Tagen wollen Sie in den nächsten drei Wochen Tofu essen?|an keinem Tag|an allen 21 Tagen

C10. Paprika

An wie vielen Tagen haben Sie in den letzten drei Wochen Paprika gegessen?|an keinem Tag|an allen 21 Tagen

An wie vielen Tagen wollen Sie in den nächsten drei Wochen Paprika essen?|an keinem Tag|an allen 21 Tagen

Teil D: Konsumziele 2/9

D1. **Tee**

An wie vielen Tagen haben Sie in den letzten drei Wochen Tee getrunken?|an keinem Tag|an allen 21 Tagen

An wie vielen Tagen wollen Sie in den nächsten drei Wochen Tee trinken?|an keinem Tag|an allen 21 Tagen

D2. **Fischstäbchen**

An wie vielen Tagen haben Sie in den letzten drei Wochen Fischstäbchen gegessen?|an keinem Tag|an allen 21 Tagen

An wie vielen Tagen wollen Sie in den nächsten drei Wochen Fischstäbchen essen?|an keinem Tag|an allen 21 Tagen

D3. **Pilze**

An wie vielen Tagen haben Sie in den letzten drei Wochen Pilze gegessen?|an keinem Tag|an allen 21 Tagen

An wie vielen Tagen wollen Sie in den nächsten drei Wochen Pilze essen?|an keinem Tag|an allen 21 Tagen

D4. **salziges Gebäck**

An wie vielen Tagen haben Sie in den letzten drei Wochen salziges Gebäck gegessen?|an keinem Tag|an allen 21 Tagen

An wie vielen Tagen wollen Sie in den nächsten drei Wochen salziges Gebäck essen?|an keinem Tag|an allen 21 Tagen

D5. Gurke

An wie vielen Tagen haben Sie in den letzten drei Wochen Gurke gegessen?|an keinem Tag|an allen 21 Tagen

An wie vielen Tagen wollen Sie in den nächsten drei Wochen Gurke essen?|an keinem Tag|an allen 21 Tagen

D6. Quinoa

An wie vielen Tagen haben Sie in den letzten drei Wochen Quinoa gegessen?|an keinem Tag|an allen 21 Tagen

An wie vielen Tagen wollen Sie in den nächsten drei Wochen Quinoa essen?|an keinem Tag|an allen 21 Tagen

D7. Kartoffelbrei

An wie vielen Tagen haben Sie in den letzten drei Wochen Kartoffelbrei gegessen?|an keinem Tag|an allen 21 Tagen

An wie vielen Tagen wollen Sie in den nächsten drei Wochen Kartoffelbrei essen?|an keinem Tag|an allen 21 Tagen

D8. Schokonüsse

An wie vielen Tagen haben Sie in den letzten drei Wochen Schokonüsse gegessen?|an keinem Tag|an allen 21 Tagen

An wie vielen Tagen wollen Sie in den nächsten drei Wochen Schokonüsse essen?|an keinem Tag|an allen 21 Tagen

D9. Hummus

An wie vielen Tagen haben Sie in den letzten drei Wochen Hummus gegessen?|an keinem Tag|an allen 21 Tagen

An wie vielen Tagen wollen Sie in den nächsten drei Wochen Hummus essen?|an keinem Tag|an allen 21 Tagen

D10. Kiwi

An wie vielen Tagen haben Sie in den letzten drei Wochen Kiwi gegessen?|an keinem Tag|an allen 21 Tagen

An wie vielen Tagen wollen Sie in den nächsten drei Wochen Kiwi essen?|an keinem Tag|an allen 21 Tagen

Teil E: Konsumziele 3/9

E1. Lasagne

An wie vielen Tagen haben Sie in den letzten drei Wochen Lasagne gegessen?|an keinem Tag|an allen 21 Tagen

An wie vielen Tagen wollen Sie in den nächsten drei Wochen Lasagne essen?|an keinem Tag|an allen 21 Tagen

E2. Nüsse im Teigmantel

An wie vielen Tagen haben Sie in den letzten drei Wochen Nüsse im Teigmantel gegessen?|an keinem Tag|an allen 21 Tagen

An wie vielen Tagen wollen Sie in den nächsten drei Wochen Nüsse im Teigmantel essen?|an keinem Tag|an allen 21 Tagen

E3. Zucchini

An wie vielen Tagen haben Sie in den letzten drei Wochen Zucchini gegessen?|an keinem Tag|an allen 21 Tagen

An wie vielen Tagen wollen Sie in den nächsten drei Wochen Zucchini essen?|an keinem Tag|an allen 21 Tagen

For peer review only - http://bmjopen.bmj.com/site/about/guidelines.xhtml

E4. Wassereis

An wie vielen Tagen haben Sie in den letzten drei Wochen Wassereis gegessen?|an keinem Tag|an allen 21 Tagen

An wie vielen Tagen wollen Sie in den nächsten drei Wochen Wassereis essen?|an keinem Tag|an allen 21 Tagen

E5. Linsen

An wie vielen Tagen haben Sie in den letzten drei Wochen Linsen gegessen?|an keinem Tag|an allen 21 Tagen

An wie vielen Tagen wollen Sie in den nächsten drei Wochen Linsen essen?|an keinem Tag|an allen 21 Tagen

E6. Avocado

An wie vielen Tagen haben Sie in den letzten drei Wochen Avocado gegessen?|an keinem Tag|an allen 21 Tagen

An wie vielen Tagen wollen Sie in den nächsten drei Wochen Avocado essen?|an keinem Tag|an allen 21 Tagen

E7. Möhren

An wie vielen Tagen haben Sie in den letzten drei Wochen Möhren gegessen?|an keinem Tag|an allen 21 Tagen

An wie vielen Tagen wollen Sie in den nächsten drei Wochen Möhren essen?|an keinem Tag|an allen 21 Tagen

E8. Cappuccino

An wie vielen Tagen haben Sie in den letzten drei Wochen Cappuccino getrunken?|an keinem Tag|an allen 21 Tagen

An wie vielen Tagen wollen Sie in den nächsten drei Wochen Cappuccino trinken?|an keinem Tag|an allen 21 Tagen

E9. Salzcracker

An wie vielen Tagen haben Sie in den letzten drei Wochen Cracker gegessen?|an keinem Tag|an allen 21 Tagen

An wie vielen Tagen wollen Sie in den nächsten drei Wochen Cracker essen?|an keinem Tag|an allen 21 Tagen

E10. Milch

An wie vielen Tagen haben Sie in den letzten drei Wochen Milch getrunken?|an keinem Tag|an allen 21 Tagen

An wie vielen Tagen wollen Sie in den nächsten drei Wochen Milch trinken?|an keinem Tag|an allen 21 Tagen

Teil F: Konsumziele 4/9

F1. Karamellriegel

An wie vielen Tagen haben Sie in den letzten drei Wochen Karamellriegel gegessen?|an keinem Tag|an allen 21 Tagen

An wie vielen Tagen wollen Sie in den nächsten drei Wochen Karamellriegel essen?|an keinem Tag|an allen 21 Tagen

F2. Marmelade

An wie vielen Tagen haben Sie in den letzten drei Wochen Marmelade gegessen?|an keinem Tag|an allen 21 Tagen

An wie vielen Tagen wollen Sie in den nächsten drei Wochen Marmelade essen?|an keinem Tag|an allen 21 Tagen

For peer review only - http://bmjopen.bmj.com/site/about/guidelines.xhtml

F3. Frühlingsrollen

An wie vielen Tagen haben Sie in den letzten drei Wochen Frühlingsrollen gegessen?|an keinem Tag|an allen 21 Tagen

An wie vielen Tagen wollen Sie in den nächsten drei Wochen Frühlingsrollen essen?|an keinem Tag|an allen 21 Tagen

F4. Butter

An wie vielen Tagen haben Sie in den letzten drei Wochen Butter gegessen?|an keinem Tag|an allen 21 Tagen

An wie vielen Tagen wollen Sie in den nächsten drei Wochen Butter essen?|an keinem Tag|an allen 21 Tagen

F5. Mandarine/ Orange

An wie vielen Tagen haben Sie in den letzten drei Wochen Mandarine/ Orange gegessen?|an keinem Tag|an allen 21 Tagen

An wie vielen Tagen wollen Sie in den nächsten drei Wochen Mandarine/ Orange essen?|an keinem Tag|an allen 21 Tagen

F6. Müsliriegel

An wie vielen Tagen haben Sie in den letzten drei Wochen Müsliriegel gegessen?|an keinem Tag|an allen 21 Tagen

An wie vielen Tagen wollen Sie in den nächsten drei Wochen Müsliriegel essen?|an keinem Tag|an allen 21 Tagen

F7. Weingummis

An wie vielen Tagen haben Sie in den letzten drei Wochen Weingummis gegessen?|an keinem Tag|an allen 21 Tagen

An wie vielen Tagen wollen Sie in den nächsten drei Wochen Weingummis essen?|an keinem Tag|an allen 21 Tagen

F8. Schnitzel

An wie vielen Tagen haben Sie in den letzten drei Wochen Schnitzel gegessen?|an keinem Tag|an allen 21 Tagen

An wie vielen Tagen wollen Sie in den nächsten drei Wochen Schnitzel essen?|an keinem Tag|an allen 21 Tagen

F9. Chips

An wie vielen Tagen haben Sie in den letzten drei Wochen Chips gegessen?|an keinem Tag|an allen 21 Tagen

An wie vielen Tagen wollen Sie in den nächsten drei Wochen Chips essen?|an keinem Tag|an allen 21 Tagen

F10. Gemüsesuppe

An wie vielen Tagen haben Sie in den letzten drei Wochen Gemüsesuppe gegessen?|an keinem Tag|an allen 21 Tagen

An wie vielen Tagen wollen Sie in den nächsten drei Wochen Gemüsesuppe essen?|an keinem Tag|an allen 21 Tagen

Teil G: Konsumziele 5/9

G1. Ei

An wie vielen Tagen haben Sie in den letzten drei Wochen Eier (in jeglicher Zubereitungsform) gegessen?|an keinem Tag|an allen 21 Tagen

An wie vielen Tagen wollen Sie in den nächsten drei Wochen Eier (in jeglicher Zubereitungsform) essen?|an keinem Tag|an allen 21 Tagen

For peer review only - http://bmjopen.bmj.com/site/about/guidelines.xhtml

G2. Tortellini

An wie vielen Tagen haben Sie in den letzten drei Wochen Tortellini gegessen?|an keinem Tag|an allen 21 Tagen

An wie vielen Tagen wollen Sie in den nächsten drei Wochen Tortellini essen?|an keinem Tag|an allen 21 Tagen

G3. Bratwurst

An wie vielen Tagen haben Sie in den letzten drei Wochen Bratwurst gegessen?|an keinem Tag|an allen 21 Tagen

An wie vielen Tagen wollen Sie in den nächsten drei Wochen Bratwurst essen?|an keinem Tag|an allen 21 Tagen

G4. Schokoladencornflakes

An wie vielen Tagen haben Sie in den letzten drei Wochen Schokoladencornflakes gegessen?|an keinem Tag|an allen 21 Tagen

An wie vielen Tagen wollen Sie in den nächsten drei Wochen Schokoladencornflakes essen?|an keinem Tag|an allen 21 Tagen

G5. Hähnchenfleisch

An wie vielen Tagen haben Sie in den letzten drei Wochen Hähnchenfleisch gegessen?|an keinem Tag|an allen 21 Tagen

An wie vielen Tagen wollen Sie in den nächsten drei Wochen Hähnchenfleisch essen?|an keinem Tag|an allen 21 Tagen

G6. Softdrinks

An wie vielen Tagen haben Sie in den letzten drei Wochen Softdrinks getrunken?|an keinem Tag|an allen 21 Tagen

An wie vielen Tagen wollen Sie in den nächsten drei Wochen Softdrinks trinken?|an keinem Tag|an allen 21 Tagen

G7. Popcorn

An wie vielen Tagen haben Sie in den letzten drei Wochen Popcorn gegessen?|an keinem Tag|an allen 21 Tagen

An wie vielen Tagen wollen Sie in den nächsten drei Wochen Popcorn essen?|an keinem Tag|an allen 21 Tagen

G8. Pfannkuchen/ Palatschinken

An wie vielen Tagen haben Sie in den letzten drei Wochen Pfannkuchen/ Palatschinken gegessen?|an keinem Tag|an allen 21 Tagen

An wie vielen Tagen wollen Sie in den nächsten drei Wochen Pfannkuchen/ Palatschinken essen?|an keinem Tag|an allen 21 Tagen

G9. Banane

An wie vielen Tagen haben Sie in den letzten drei Wochen Banane gegessen?|an keinem Tag|an allen 21 Tagen

An wie vielen Tagen wollen Sie in den nächsten drei Wochen Banane essen?|an keinem Tag|an allen 21 Tagen

G10. Käse

An wie vielen Tagen haben Sie in den letzten drei Wochen Käse gegessen?|an keinem Tag|an allen 21 Tagen

An wie vielen Tagen wollen Sie in den nächsten drei Wochen Käse essen?|an keinem Tag|an allen 21 Tagen

Teil H: Konsumziele 6/9

H1. Knödel

An wie vielen Tagen haben Sie in den letzten drei Wochen Knödel gegessen?|an keinem Tag|an allen 21 Tagen

An wie vielen Tagen wollen Sie in den nächsten drei Wochen Knödel essen?|an keinem Tag|an allen 21 Tagen

H2. Pralinen

An wie vielen Tagen haben Sie in den letzten drei Wochen Pralinen gegessen?|an keinem Tag|an allen 21 Tagen

An wie vielen Tagen wollen Sie in den nächsten drei Wochen Pralinen essen?|an keinem Tag|an allen 21 Tagen

H3. Burger

An wie vielen Tagen haben Sie in den letzten drei Wochen Burger gegessen?|an keinem Tag|an allen 21 Tagen

An wie vielen Tagen wollen Sie in den nächsten drei Wochen Burger essen?|an keinem Tag|an allen 21 Tagen

H4. Weintrauben

An wie vielen Tagen haben Sie in den letzten drei Wochen Weintrauben gegessen?|an keinem Tag|an allen 21 Tagen

An wie vielen Tagen wollen Sie in den nächsten drei Wochen Weintrauben essen?|an keinem Tag|an allen 21 Tagen

H5. Gnocchi

An wie vielen Tagen haben Sie in den letzten drei Wochen Gnocchi gegessen?|an keinem Tag|an allen 21 Tagen

An wie vielen Tagen wollen Sie in den nächsten drei Wochen Gnocchi essen?|an keinem Tag|an allen 21 Tagen

H6. Pommes

An wie vielen Tagen haben Sie in den letzten drei Wochen Pommes gegessen?|an keinem Tag|an allen 21 Tagen

An wie vielen Tagen wollen Sie in den nächsten drei Wochen Pommes essen?|an keinem Tag|an allen 21 Tagen

H7. Waffeln

An wie vielen Tagen haben Sie in den letzten drei Wochen Waffeln gegessen?|an keinem Tag|an allen 21 Tagen

An wie vielen Tagen wollen Sie in den nächsten drei Wochen Waffeln essen?|an keinem Tag|an allen 21 Tagen

H8. Käsespätzle

An wie vielen Tagen haben Sie in den letzten drei Wochen Käsespätzle gegessen?|an keinem Tag|an allen 21 Tagen

An wie vielen Tagen wollen Sie in den nächsten drei Wochen Käsespätzle essen?|an keinem Tag|an allen 21 Tagen

H9. Speiseeis

An wie vielen Tagen haben Sie in den letzten drei Wochen Speiseeis gegessen?|an keinem Tag|an allen 21 Tagen

An wie vielen Tagen wollen Sie in den nächsten drei Wochen Speiseeis essen?lan keinem Taglan allen 21 Tagen

H10. Sushi

An wie vielen Tagen haben Sie in den letzten drei Wochen Sushi gegessen?lan keinem Taglan allen 21 Tagen

An wie vielen Tagen wollen Sie in den nächsten drei Wochen Sushi essen?lan keinem Taglan allen 21 Tagen

Teil I: Konsumziele 7/9

I1. Äpfel

An wie vielen Tagen haben Sie in den letzten drei Wochen Äpfel gegessen?lan keinem Taglan allen 21 Tagen

An wie vielen Tagen wollen Sie in den nächsten drei Wochen Äpfel essen?lan keinem Taglan allen 21 Tagen

I2. Salat

An wie vielen Tagen haben Sie in den letzten drei Wochen Salat gegessen?lan keinem Taglan allen 21 Tagen

An wie vielen Tagen wollen Sie in den nächsten drei Wochen Salat essen?lan keinem Taglan allen 21 Tagen

I3. Fruchtsaft

An wie vielen Tagen haben Sie in den letzten drei Wochen Fruchtsaft getrunken?lan keinem Taglan allen 21 Tagen

An wie vielen Tagen wollen Sie in den nächsten drei Wochen Fruchtsaft trinken?lan keinem Taglan allen 21 Tagen

I4. Kartoffeln

An wie vielen Tagen haben Sie in den letzten drei Wochen Kartoffeln gegessen?|an keinem Tag|an allen 21 Tagen

An wie vielen Tagen wollen Sie in den nächsten drei Wochen Kartoffeln essen?|an keinem Tag|an allen 21 Tagen

I5. Leberkäse

An wie vielen Tagen haben Sie in den letzten drei Wochen Leberkäse gegessen?|an keinem Tag|an allen 21 Tagen

An wie vielen Tagen wollen Sie in den nächsten drei Wochen Leberkäse essen?|an keinem Tag|an allen 21 Tagen

I6. Nachos

An wie vielen Tagen haben Sie in den letzten drei Wochen Nachos gegessen?|an keinem Tag|an allen 21 Tagen

An wie vielen Tagen wollen Sie in den nächsten drei Wochen Nachos essen?|an keinem Tag|an allen 21 Tagen

I7. Vollmilchschokolade

An wie vielen Tagen haben Sie in den letzten drei Wochen Vollmilchschokolade gegessen?|an keinem Tag|an allen 21 Tagen

An wie vielen Tagen wollen Sie in den nächsten drei Wochen Vollmilchschokolade essen?|an keinem Tag|an allen 21 Tagen

I8. Nudeln

An wie vielen Tagen haben Sie in den letzten drei Wochen Nudeln gegessen?|an keinem Tag|an allen 21 Tagen

An wie vielen Tagen wollen Sie in den nächsten drei Wochen Nudeln essen?lan keinem Taglan allen 21 Tagen

I9. Nougatcreme

An wie vielen Tagen haben Sie in den letzten drei Wochen Nougatcreme gegessen?lan keinem Taglan allen 21 Tagen

An wie vielen Tagen wollen Sie in den nächsten drei Wochen Nougatcreme essen?lan keinem Taglan allen 21 Tagen

I10. Wraps

An wie vielen Tagen haben Sie in den letzten drei Wochen Wraps gegessen?lan keinem Taglan allen 21 Tagen

An wie vielen Tagen wollen Sie in den nächsten drei Wochen Wraps essen?lan keinem Taglan allen 21 Tagen

Teil J: Konsumziele 8/9

J1. Kekse

An wie vielen Tagen haben Sie in den letzten drei Wochen Kekse gegessen?lan keinem Taglan allen 21 Tagen

An wie vielen Tagen wollen Sie in den nächsten drei Wochen Kekse essen?lan keinem Taglan allen 21 Tagen

J2. Beeren

An wie vielen Tagen haben Sie in den letzten drei Wochen Beeren gegessen?lan keinem Taglan allen 21 Tagen

An wie vielen Tagen wollen Sie in den nächsten drei Wochen Beeren essen?lan keinem Taglan allen 21 Tagen

J3. Ofenbaguette

An wie vielen Tagen haben Sie in den letzten drei Wochen Ofenbaguette gegessen?|an keinem Tag|an allen 21 Tagen

An wie vielen Tagen wollen Sie in den nächsten drei Wochen Ofenbaguette essen?|an keinem Tag|an allen 21 Tagen

J4. Muffin

An wie vielen Tagen haben Sie in den letzten drei Wochen Muffins gegessen?|an keinem Tag|an allen 21 Tagen

An wie vielen Tagen wollen Sie in den nächsten drei Wochen Muffins essen?|an keinem Tag|an allen 21 Tagen

J5. süßes Gebäck

An wie vielen Tagen haben Sie in den letzten drei Wochen süßes Gebäck gegessen?|an keinem Tag|an allen 21 Tagen

An wie vielen Tagen wollen Sie in den nächsten drei Wochen süßes Gebäck essen?|an keinem Tag|an allen 21 Tagen

J6. Wurst

An wie vielen Tagen haben Sie in den letzten drei Wochen Wurst gegessen?|an keinem Tag|an allen 21 Tagen

An wie vielen Tagen wollen Sie in den nächsten drei Wochen Wurst essen?|an keinem Tag|an allen 21 Tagen

J7. Kuchen

An wie vielen Tagen haben Sie in den letzten drei Wochen Kuchen gegessen?|an keinem Tag|an allen 21 Tagen

An wie vielen Tagen wollen Sie in den nächsten drei Wochen Kuchen essen?|an keinem Tag|an allen 21 Tagen

J8. Kichererbsen

An wie vielen Tagen haben Sie in den letzten drei Wochen Kichererbsen gegessen?|an keinem Tag|an allen 21 Tagen

An wie vielen Tagen wollen Sie in den nächsten drei Wochen Kichererbsen essen?|an keinem Tag|an allen 21 Tagen

J9. Bitterschokolade

An wie vielen Tagen haben Sie in den letzten drei Wochen Bitterschokolade gegessen?|an keinem Tag|an allen 21 Tagen

An wie vielen Tagen wollen Sie in den nächsten drei Wochen Bitterschokolade essen?|an keinem Tag|an allen 21 Tagen

J10. Bratnudeln

An wie vielen Tagen haben Sie in den letzten drei Wochen Bratnudeln gegessen?|an keinem Tag|an allen 21 Tagen

An wie vielen Tagen wollen Sie in den nächsten drei Wochen Bratnudeln essen?|an keinem Tag|an allen 21 Tagen

Teil K: Konsumziele 9/9

K1. Baguette

An wie vielen Tagen haben Sie in den letzten drei Wochen Baguette gegessen?|an keinem Tag|an allen 21 Tagen

An wie vielen Tagen wollen Sie in den nächsten drei Wochen Baguette essen?|an keinem Tag|an allen 21 Tagen

K2. Döner

An wie vielen Tagen haben Sie in den letzten drei Wochen Döner gegessen?|an keinem Tag|an allen 21 Tagen

An wie vielen Tagen wollen Sie in den nächsten drei Wochen Döner essen?|an keinem Tag|an allen 21 Tagen

K3. Toast

An wie vielen Tagen haben Sie in den letzten drei Wochen Toast gegessen?|an keinem Tag|an allen 21 Tagen

An wie vielen Tagen wollen Sie in den nächsten drei Wochen Toast essen?|an keinem Tag|an allen 21 Tagen

K4. Rindfleisch

An wie vielen Tagen haben Sie in den letzten drei Wochen Rindfleisch gegessen?|an keinem Tag|an allen 21 Tagen

An wie vielen Tagen wollen Sie in den nächsten drei Wochen Rindfleisch essen?|an keinem Tag|an allen 21 Tagen

K5. Joghurt

An wie vielen Tagen haben Sie in den letzten drei Wochen Joghurt gegessen?|an keinem Tag|an allen 21 Tagen

An wie vielen Tagen wollen Sie in den nächsten drei Wochen Joghurt essen?|an keinem Tag|an allen 21 Tagen

K6. Schokoladenwaffeln

An wie vielen Tagen haben Sie in den letzten drei Wochen Schokoladenwaffeln gegessen?|an keinem Tag|an allen 21 Tagen

An wie vielen Tagen wollen Sie in den nächsten drei Wochen Schokoladenwaffeln essen?|an keinem Tag|an allen 21 Tagen

K7. Fleischpflanzerl/ Fleischfrikadellen

An wie vielen Tagen haben Sie in den letzten drei Wochen Fleischpflanzerl/ Fleischfrikadellen gegessen?|an keinem Tag|an allen 21 Tagen

An wie vielen Tagen wollen Sie in den nächsten drei Wochen Fleischpflanzerl/ Fleischfrikadellen essen?|an keinem Tag|an allen 21 Tagen

K8. Gemüselaibchen/ Gemüsefrikadellen

An wie vielen Tagen haben Sie in den letzten drei Wochen Gemüselaibchen/ Gemüsefrikadellen gegessen?|an keinem Tag|an allen 21 Tagen

An wie vielen Tagen wollen Sie in den nächsten drei Wochen Gemüselaibchen/ Gemüsefrikadellen essen?|an keinem Tag|an allen 21 Tagen

K9. Vollkornbrot

An wie vielen Tagen haben Sie in den letzten drei Wochen Vollkornbrot gegessen?|an keinem Tag|an allen 21 Tagen

An wie vielen Tagen wollen Sie in den nächsten drei Wochen Vollkornbrot essen?|an keinem Tag|an allen 21 Tagen

K10. Schokoladenriegel

An wie vielen Tagen haben Sie in den letzten drei Wochen Schokoladenriegel gegessen?|an keinem Tag|an allen 21 Tagen

An wie vielen Tagen wollen Sie in den nächsten drei Wochen Schokoladenriegel essen?|an keinem Tag|an allen 21 Tagen

For peer review only - http://bmjopen.bmj.com/site/about/guidelines.xhtml

Vielen Dank für Ihre Teilnahme an der ersten Befragung. Wir werden die erhobenen Daten zu Ihren Konsumzielen nun auswerten und uns bei Ihnen melden, um Sie über die weitere Studienteilnahme zu informieren und einen Termin für die Besprechung der Hauptstudienphase zu vereinbaren.

Sie können dieses Fenster nun schließen.

Fachbereich Psychologie
Centre for Cognitive Neuroscience,
Fachbereich für Psychologie

Univ.-Prof. Dr. Jens Blechert
jens.blechert@sbg.ac.at
Tel.: +43 / (0) 662 /8044 - 5163
Fax.: +43 / (0) 662 / 8044 – 5126

Hellbrunnerstraße 34
A-5020 Salzburg – Austria

TeilnehmerInneninformation und Einwilligungserklärung

zur Teilnahme an der Studie:

‚Kognitiv-Affektive Mechanismen von Essregulationstrainings ‘

Interne Bezeichnung: Cognitive-affective mechanisms of food biases trainings (AAI)

For peer review only

Liebe/r Interessent/in,

wir laden Sie ein an der oben genannten Studie teilzunehmen.

Im Folgenden finden Sie einige Informationen, die für Sie von Bedeutung sind, wenn Sie sich für eine Teilnahme interessieren.

Bitte unterschreiben Sie diese Einverständniserklärung nur

- wenn Sie Art und Ablauf der Studie verstanden haben
- wenn Sie bereit sind, einer Teilnahme zuzustimmen und
- wenn Sie sich über Ihre Rechte als Teilnehmer im Klaren sind.

Allgemeine Informationen und Ziele der Studie

Die Ernährungspsychologie beschäftigt sich mit Grundlagen und Behandlungsmöglichkeiten von ungesunden Essensentscheidungen sowie mit Ess- und Gewichtsstörungen. Da das Verlangen nach schmackhaften Nahrungsmitteln das Essverhalten im Alltag beeinflusst, ist es wichtig, dass wir ein besseres Verständnis für diese Zusammenhänge gewinnen, auch um Abhilfe verschaffen zu können. In dieser Studie geht es speziell darum, wie automatische Annäherungstendenzen zu Essensreizen optimal erfasst werden können, was die zugrundeliegenden Mechanismen sind und wie das Annäherungsverhalten reduziert werden kann.

Ablauf der Studie

Bei Ihrer Studienteilnahme werden wir Sie bitten, verschiedene Fragebögen zum psychischen Befinden auszufüllen. Des Weiteren wird Ihre Teilnahme nachfolgende Prozeduren beinhalten:

For peer review only - http://bmjopen.bmj.com/site/about/guidelines.xhtml

- *Betrachten von Bildern von Nahrungsmitteln*: Sie werden verschiedene Nahrungsmittel am Bildschirm betrachten und bewerten.
- *Reaktionszeitmessung via Smartphone*: Sie werden gebeten, mit schnellen Bewegungen des Smartphones auf Bilder von Nahrungsmitteln zu reagieren.
- *Fragebögen via Smartphone*: Zweimal pro Tag werden Sie gebeten über das Smartphone verschiedene Fragen zu Ihrem momentanen Empfinden und Ihrem vergangenen Essverhalten zu beantworten.
- *Reaktionszeittraining in einer von zwei Gruppen.* Ergänzend zu den oben beschriebenen Studienteilen werden Sie zudem per Zufallsauswahl *einer von zwei Studiengruppen zugeteilt*. Aus wissenschaftlichen Gründen ist eine zufällige Gruppenzuteilung notwendig, Sie können sich also nicht eine der Gruppen aussuchen. Beide Gruppen beinhalten die Bearbeitung von Reaktionszeitaufgaben am Smartphone über mehrere Termine hinweg, jedoch unterscheidet sich die Art der Reaktionszeitaufgabe geringfügig. Am Ende der Studie erklären wir Ihnen ausführlich die Hintergründe der Trainings.

Freiwilligkeit der Teilnahme

Ihre Teilnahme an dieser Erhebung ist freiwillig. Sie können diese jederzeit, ohne Angabe von Gründen, beenden. Die Ablehnung Ihrer Teilnahme oder ein vorzeitiges Beenden kann mündlich oder schriftlich (siehe Kontaktdaten am Ende des Dokuments) erfolgen, hat keine nachteiligen Folgen und Sie werden anteilig kompensiert. Sie können Ihre Einwilligung zur Speicherung der Daten bis zum Ende der Datenerhebung widerrufen, ohne dass Ihnen daraus Nachteile entstehen.

Ausschlusskriterien

Sie können an unserer Studie teilnehmen, wenn Sie zwischen 18 und 60 Jahren alt sind und ausreichende Deutschkenntnisse besitzen, um die Instruktionen während der Studie zu verstehen. Nicht teilnehmen können außerdem Personen, die schwanger sind, in ihrer Entscheidungsfähigkeit eingeschränkt sind oder wenn eine diagnostizierte Essstörung vorliegt.

Pflichten bei einer Teilnahme

Als Studienteilnehmer/-innen werden Sie gebeten, den Anweisungen der Studienleitung so weit wie möglich zu folgen und die Fragebögen aufrichtig zu beantworten. Sollten sich während der Studie Probleme und Schwierigkeiten ergeben, informieren Sie bitte die Studienleitung darüber.

Rechte bei einer Teilnahme

Ihre Teilnahme erfolgt freiwillig und Sie können sich jederzeit, auch ohne Angabe von Gründen, aus der Studie zurückziehen, und/ oder eine Löschung der erfassten Daten verlangen, ohne dass Ihnen daraus Nachteile irgendwelcher Art entstehen.

Nutzen bei einer Teilnahme

Ihre Teilnahme an dieser Studie dient der klinisch-psychologischen Wissenschaft. Neue Erkenntnisse sollen zum besseren Verständnis der Ursachen von Überessen und starkem Verlangen nach schmackhaftem Essen beitragen. Außerdem profitieren Sie durch die Teilnahme an dem Reaktionszeittraining bezüglich Ihres

For peer review only - http://bmjopen.bmj.com/site/about/guidelines.xhtml

selbstgesteckten Ernährungsziels. Nach Studienende erhalten Sie auf Wunsch ausführliche schriftliche Informationen über Sinn und Zweck der Studie sowie eine individualisierte Rückmeldung und Erklärung ihrer Ergebnisse. Des Weiteren erhalten Sie eine Aufwandsentschädigung.

Aufwandsentschädigung

Während wir davon ausgehen, dass Ihr Essverhalten vom Reaktionszeittraining profitieren wird, entsteht durch die Beantwortung der Fragebögen jedoch zusätzlicher zeitlicher Aufwand, für den wir sie finanziell kompensieren. Abhängig von der Vollständigkeit ihr Daten erhalten Sie sechs bis acht VP-Stunden beziehungsweise zwischen 40 und 60 Euro.

Mögliche Risiken und Unannehmlichkeiten

Die oben beschriebenen Prozeduren sind nicht gesundheitsschädlich und entsprechen wissenschaftlichen Standards. Sie können vorrübergehend negative physische und emotionale Empfindungen hervorrufen. Diese Empfindungen sowie spätere diesbezügliche Erinnerungen sind jedoch erfahrungsgemäß vorübergehender Natur. Sollten Sie unerwarteter Weise unter anhaltenden Belastungen aufgrund Ihrer Studienteilnahme leiden, so melden Sie sich umgehend bei einer der unten aufgeführten Kontaktpersonen.

Vertraulichkeit und Schutz der Daten

Bei den Daten ist zu unterscheiden zwischen personenbezogenen Daten, mit denen Sie direkt identifizierbar sind (z.B.: Name, Telefonnummer, Adresse), und pseudonymisierten (verschlüsselten) Daten, bei denen alle Informationen, die direkten Rückschluss auf Ihre Identität zulassen, durch einen Teilnahmecode ersetzt werden. Der **‚Schlüssel‘** (Abgleich von Pseudonym mit personenbezogenen Daten) ist nur der Studienleitung zugänglich, wird nach dem Stand der Technik in passwort-geschützten Dokumenten geschützt und getrennt von den verschlüsselten Datensätzen aufbewahrt. Sämtliche Personen, die Zugang zu personenbezogenen Daten erhalten, unterliegen im Umgang mit den Daten dem österreichischen und europäischen Datenschutzrecht und sind dem Datengeheimnis verpflichtet.

Im Rahmen dieser Studie erfolgt die Verarbeitung von folgenden Daten:

- *Personenbezogenen Daten*: Name, E-mail, Telefonnummer. Diese Daten erleichtern uns die Studienadministration und die Sicherstellung einer hohen Datenqualität.
- *Pseudonymisierte Daten*: Alle Eingaben, die Sie über die App ‚m-Path‘ machen, werden dort unter einem selbstgewählten Teilnahmecode („Pseudonym“) auf Servern der Universität Leuven (Belgien) gespeichert. Ihre Daten sind also innerhalb der App nicht Ihrer Person zu zuordnen. Auch die Daten in der App ‚Picture Game‘ werden nur über einen pseudonymisierten Teilnahmecode gespeichert und über einen Account der Studienleitung von Google firebase übertragen. Für beide Apps gilt: Eine Zuordnung zu Ihrer Person ist nur mit zusätzlichen Daten möglich, die ausschließlich Mitarbeitenden der PLUS vorliegen (‚Schlüssel‘, siehe oben).

For peer review only - http://bmjopen.bmj.com/site/about/guidelines.xhtml

Ihnen steht bezüglich Ihrer bei uns gespeicherten pseudonymisierten Daten grundsätzlich das Recht auf Auskunft, Richtigstellung, Löschung und Einschränkung zu. Sie können Ihre Einwilligung auch jederzeit widerrufen. Ein Widerruf hat zur Folge, dass wir Ihre Daten ab diesem Zeitpunkt zu den oben genannten Zwecken nicht mehr verarbeiten. Für einen Widerruf wenden Sie sich bitte an die unten angeführte Studienleitung. Wenn Sie glauben, dass wir gegen datenschutzrechtliche Vorschriften verstoßen, können Sie sich bei dem Datenschutzbeauftragten der Universität Salzburg (datenschutz@sbg.ac.at) oder bei einer Datenschutzbehörde beschweren. Weitere Informationen finden Sie unter www.uni-salzburg.at/impressum.

Wir weisen darauf hin, dass wissenschaftliche Publikationen geplant sind und dabei Studienergebnisse in ausschließlich anonymer Weise veröffentlicht werden.

Die personenbezogenen Daten und der zugehörige Schlüssel wird **1 Jahr nach Studienende gelöscht**. Ebenso werden alle Daten in der App zu dem Zeitpunkt gelöscht. Damit erlöschen auch ihre Möglichkeiten der Einsicht/Berichtigung/Export/Löschung dieser Daten, da wir Sie ihren Daten dann nicht mehr zuordnen können (da der ‚Schlüssel' gelöscht wurde). Wir behalten einzig ihren Namen und diese Einverständniserklärung als Dokumentation ihrer Studienteilname für **30 Jahre**.

Welche Kontaktpersonen stehen zur Verfügung?

Bei anfälligen Fragen, die während oder nach Abschluss der Studie auftreten, können Sie sich unter gesundheitspsychologie07@plus.ac.at oder der +43 677 616 767 07 melden und jederzeit an die Studienleitung wenden:

- Hannah van Alebeek, MSc., Fachbereich Psychologie, Universität Salzburg, Hellbrunnerstrasse 34, 5020 Salzburg, hannah.vanalebeek@plus.ac.at

Wir freuen uns auf die Zusammenarbeit!

Diese Studie wurde von der Ethikkommission der Universität Salzburg evaluiert.
Kontaktperson: Mag.[a] Clara Gröblacher, Kapitelgasse 4, A-5020 Salzburg, Tel: +43-662-8044 2391, clara.groeblacher@sbg.ac.at

Einverständniserklärung

Ich habe die oben beschriebenen Informationen vollumfänglich gelesen und verstanden.

Meine Teilnahme erfolgt freiwillig und ich weiß, dass ich mich jederzeit, auch ohne Angabe von Gründen, von der Studie zurückziehen kann, ohne dass mir daraus Nachteile irgendwelcher Art entstehen.

Ich bin bereit, an dieser Studie teilzunehmen und bin mit der Erhebung und Verwendung persönlicher Daten nach Maßgabe der TeilnehmerInneninformation einverstanden.

Ort/Datum ______________________________

Name TeilnehmerIn ______________________________

Unterschrift TeilnehmerIn ______________________________

Unterschrift Studienverantwortliche Person ______________________________

For peer review only - http://bmjopen.bmj.com/site/about/guidelines.xhtml

– Dieses Dokument bleibt bei der Versuchsleitung –

Einverständniserklärung

Ich habe die oben beschriebenen Informationen vollumfänglich gelesen und verstanden. Außerdem nehme ich zur Kenntnis, dass ich für Wege zum/vom Untersuchungsort, nicht wege-/unfallversichert bin.

Meine Teilnahme erfolgt freiwillig und ich weiß, dass ich mich jederzeit, auch ohne Angabe von Gründen, von der Studie zurückziehen kann, ohne dass mir daraus Nachteile irgendwelcher Art entstehen.

Ich bin bereit, an dieser Studie teilzunehmen und bin mit der Erhebung und Verwendung persönlicher Daten nach Maßgabe der TeilnehmerInneninformation einverstanden. Eine Kopie dieser Studieninformation und Einverständniserklärung wurde mir ausgehändigt.

Ort/Datum ______________________________

Name TeilnehmerIn ______________________________

Unterschrift TeilnehmerIn ______________________________

Unterschrift
Studienverantwortliche Person ______________________________

For peer review only

For peer review only - http://bmjopen.bmj.com/site/about/guidelines.xhtml

Reporting checklist for protocol of a clinical trial.

Based on the SPIRIT guidelines.

Instructions to authors

Complete this checklist by entering the page numbers from your manuscript where readers will find each of the items listed below.

Your article may not currently address all the items on the checklist. Please modify your text to include the missing information. If you are certain that an item does not apply, please write "n/a" and provide a short explanation.

Upload your completed checklist as an extra file when you submit to a journal.

In your methods section, say that you used the SPIRITreporting guidelines, and cite them as:

Chan A-W, Tetzlaff JM, Gøtzsche PC, Altman DG, Mann H, Berlin J, Dickersin K, Hróbjartsson A, Schulz KF, Parulekar WR, Krleža-Jerić K, Laupacis A, Moher D. SPIRIT 2013 Explanation and Elaboration: Guidance for protocols of clinical trials. BMJ. 2013;346:e7586

		Reporting Item	Page Number
**Administrative information**			
Title	#1	Descriptive title identifying the study design, population, interventions, and, if applicable, trial acronym	1
Trial registration	#2a	Trial identifier and registry name. If not yet registered,	2

For peer review only

		name of intended registry	
Trial registration: data set	#2b	All items from the World Health Organization Trial Registration Data Set	n/a
Protocol version	#3	Date and version identifier	n/a
Funding	#4	Sources and types of financial, material, and other support	12
Roles and responsibilities: contributorship	#5a	Names, affiliations, and roles of protocol contributors	12
Roles and responsibilities: sponsor contact information	#5b	Name and contact information for the trial sponsor	1
Roles and responsibilities: sponsor and funder	#5c	Role of study sponsor and funders, if any, in study design; collection, management, analysis, and interpretation of data; writing of the report; and the decision to submit the report for publication, including whether they will have ultimate authority over any of these activities	12
Roles and responsibilities: committees	#5d	Composition, roles, and responsibilities of the coordinating centre, steering committee, endpoint adjudication committee, data management team, and other individuals or groups overseeing the trial, if applicable (see Item 21a for data monitoring committee)	n/a

Introduction

For peer review only

Background and rationale	#6a	Description of research question and justification for undertaking the trial, including summary of relevant studies (published and unpublished) examining benefits and harms for each intervention	3-5
Background and rationale: choice of comparators	#6b	Explanation for choice of comparators	3-5
Objectives	#7	Specific objectives or hypotheses	5
Trial design	#8	Description of trial design including type of trial (eg, parallel group, crossover, factorial, single group), allocation ratio, and framework (eg, superiority, equivalence, non-inferiority, exploratory)	5-7
**Methods: Participants, interventions, and outcomes**			
Study setting	#9	Description of study settings (eg, community clinic, academic hospital) and list of countries where data will be collected. Reference to where list of study sites can be obtained	5-8
Eligibility criteria	#10	Inclusion and exclusion criteria for participants. If applicable, eligibility criteria for study centres and individuals who will perform the interventions (eg,	5

For peer review only

		surgeons, psychotherapists)	
Interventions: description	#11a	Interventions for each group with sufficient detail to allow replication, including how and when they will be administered	6
Interventions: modifications	#11b	Criteria for discontinuing or modifying allocated interventions for a given trial participant (eg, drug dose change in response to harms, participant request, or improving / worsening disease)	n/a
Interventions: adherance	#11c	Strategies to improve adherence to intervention protocols, and any procedures for monitoring adherence (eg, drug tablet return; laboratory tests)	5
Interventions: concomitant care	#11d	Relevant concomitant care and interventions that are permitted or prohibited during the trial	n/a
Outcomes	#12	Primary, secondary, and other outcomes, including the specific measurement variable (eg, systolic blood pressure), analysis metric (eg, change from baseline, final value, time to event), method of aggregation (eg, median, proportion), and time point for each outcome. Explanation of the clinical relevance of chosen efficacy and harm outcomes is strongly recommended	9-10
Participant timeline	#13	Time schedule of enrolment, interventions (including any run-ins and washouts), assessments, and visits for participants. A schematic diagram is highly recommended (see Figure)	9

For peer review only

Sample size	#14	Estimated number of participants needed to achieve study objectives and how it was determined, including clinical and statistical assumptions supporting any sample size calculations	5
Recruitment	#15	Strategies for achieving adequate participant enrolment to reach target sample size	5
**Methods: Assignment of interventions (for controlled trials)**			
Allocation: sequence generation	#16a	Method of generating the allocation sequence (eg, computer-generated random numbers), and list of any factors for stratification. To reduce predictability of a random sequence, details of any planned restriction (eg, blocking) should be provided in a separate document that is unavailable to those who enrol participants or assign interventions	9
Allocation concealment mechanism	#16b	Mechanism of implementing the allocation sequence (eg, central telephone; sequentially numbered, opaque, sealed envelopes), describing any steps to conceal the sequence until interventions are assigned	9
Allocation: implementation	#16c	Who will generate the allocation sequence, who will enrol participants, and who will assign participants to interventions	9

For peer review only

Blinding (masking)	#17a	Who will be blinded after assignment to interventions (eg, trial participants, care providers, outcome assessors, data analysts), and how	9
Blinding (masking): emergency unblinding	#17b	If blinded, circumstances under which unblinding is permissible, and procedure for revealing a participant's allocated intervention during the trial	n/a
**Methods: Data collection, management, and analysis**			
Data collection plan	#18a	Plans for assessment and collection of outcome, baseline, and other trial data, including any related processes to promote data quality (eg, duplicate measurements, training of assessors) and a description of study instruments (eg, questionnaires, laboratory tests) along with their reliability and validity, if known. Reference to where data collection forms can be found, if not in the protocol	6-10
Data collection plan: retention	#18b	Plans to promote participant retention and complete follow-up, including list of any outcome data to be collected for participants who discontinue or deviate from intervention protocols	6-10
Data management	#19	Plans for data entry, coding, security, and storage, including any related processes to promote data quality (eg, double data entry; range checks for data values).	10

For peer review only

		Reference to where details of data management procedures can be found, if not in the protocol	
Statistics: outcomes	#20a	Statistical methods for analysing primary and secondary outcomes. Reference to where other details of the statistical analysis plan can be found, if not in the protocol	9-10
Statistics: additional analyses	#20b	Methods for any additional analyses (eg, subgroup and adjusted analyses)	9-10
Statistics: analysis population and missing data	#20c	Definition of analysis population relating to protocol non-adherence (eg, as randomised analysis), and any statistical methods to handle missing data (eg, multiple imputation)	9-10
**Methods: Monitoring**			
Data monitoring: formal committee	#21a	Composition of data monitoring committee (DMC); summary of its role and reporting structure; statement of whether it is independent from the sponsor and competing interests; and reference to where further details about its charter can be found, if not in the protocol. Alternatively, an explanation of why a DMC is not needed	n/a
Data monitoring: interim analysis	#21b	Description of any interim analyses and stopping guidelines, including who will have access to these interim results and make the final decision to terminate the trial	n/a
Harms	#22	Plans for collecting, assessing, reporting, and managing solicited and spontaneously reported adverse events and other unintended effects of trial interventions or trial	n/a

For peer review only

		conduct	
Auditing	#23	Frequency and procedures for auditing trial conduct, if any, and whether the process will be independent from investigators and the sponsor	n/a
**Ethics and dissemination**			
Research ethics approval	#24	Plans for seeking research ethics committee / institutional review board (REC / IRB) approval	2
Protocol amendments	#25	Plans for communicating important protocol modifications (eg, changes to eligibility criteria, outcomes, analyses) to relevant parties (eg, investigators, REC / IRBs, trial participants, trial registries, journals, regulators)	n/a
Consent or assent	#26a	Who will obtain informed consent or assent from potential trial participants or authorised surrogates, and how (see Item 32)	6
Consent or assent: ancillary studies	#26b	Additional consent provisions for collection and use of participant data and biological specimens in ancillary studies, if applicable	n/a
Confidentiality	#27	How personal information about potential and enrolled participants will be collected, shared, and maintained in order to protect confidentiality before, during, and after the trial	10
Declaration of	#28	Financial and other competing interests for principal	12

For peer review only

interests		investigators for the overall trial and each study site	
Data access	#29	Statement of who will have access to the final trial dataset, and disclosure of contractual agreements that limit such access for investigators	11
Ancillary and post trial care	#30	Provisions, if any, for ancillary and post-trial care, and for compensation to those who suffer harm from trial participation	n/a
Dissemination policy: trial results	#31a	Plans for investigators and sponsor to communicate trial results to participants, healthcare professionals, the public, and other relevant groups (eg, via publication, reporting in results databases, or other data sharing arrangements), including any publication restrictions	11
Dissemination policy: authorship	#31b	Authorship eligibility guidelines and any intended use of professional writers	n/a
Dissemination policy: reproducible research	#31c	Plans, if any, for granting public access to the full protocol, participant-level dataset, and statistical code	11
**Appendices**			
Informed consent materials	#32	Model consent form and other related documentation given to participants and authorised surrogates	n/a
Biological specimens	#33	Plans for collection, laboratory evaluation, and storage of biological specimens for genetic or molecular analysis in the current trial and for future use in ancillary studies, if applicable	n/a

For peer review only

The SPIRIT Explanation and Elaboration paper is distributed under the terms of the Creative Commons Attribution License CC-BY-NC. This checklist was completed on 23. November 2022 using https://www.goodreports.org/, a tool made by the EQUATOR Network in collaboration with Penelope.ai

For peer review only

BMJ Open

Study protocol for a Randomized-Controlled Trial to Test the Effectiveness of a Mobile Approach Avoidance Intervention and to Measure Approach Biases in an Ecological Momentary Assessment context

Journal:	*BMJ Open*
Manuscript ID	bmjopen-2022-070443.R2
Article Type:	Protocol
Date Submitted by the Author:	22-Mar-2023

Complete List of Authors:	Aulbach, Matthias; Paris Lodron Universitat Salzburg, Department of Psychology<br>van Alebeek, Hannah; Centre for Cognitive Neuroscience, Department of Psychology<br>Kahveci, Sercan; Centre for Cognitive Neuroscience, Department of Psychology<br>Blechert, Jens; Centre for Cognitive Neuroscience, Department of Psychology

**Primary Subject Heading**:	Nutrition and metabolism
Secondary Subject Heading:	Public health
Keywords:	Information technology < BIOTECHNOLOGY & BIOINFORMATICS, World Wide Web technology < BIOTECHNOLOGY & BIOINFORMATICS, NUTRITION & DIETETICS, PREVENTIVE MEDICINE, PUBLIC HEALTH

SCHOLARONE™
Manuscripts

Study protocol for a Randomized-Controlled Trial to Test the Effectiveness of a Mobile Approach Avoidance Intervention and to Measure Approach Biases in an Ecological Momentary Assessment context

Matthias Burkard Aulbach[1]*

Hannah van Alebeek[1]

Sercan Kahveci[1]

Jens Blechert[1]

[1]Department of Psychology, Centre for Cognitive Neuroscience, Paris-Lodron-University of Salzburg, Salzburg, Austria

*Corresponding author; contact: Matthias.aulbach@plus.ac.at

For peer review only

Word count: 4433 words

Keywords: randomized controlled trial; approach-avoidance training; eating behavior; intervention; ecological momentary assessment; m-health

ABSTRACT

Introduction: Unhealthy eating behavior is a major contributor to obesity and related diseases and is associated with a behavioral bias to approach rather than avoid desired foods, as measured with reaction time tasks. Approach-Avoidance interventions (AAI) have been proposed as a way to modify food evaluations and help people to eat in accordance with their dietary goals. Mobile implementations of AAI might be easily accessible, low threshold interventions, but their effectiveness has not been established yet.

Methods and analysis: Participants who aim to change their eating behavior are randomized to intervention or control groups. They complete six sessions of a smartphone-based AAI, in which they push (i.e., avoid) or pull (i.e., approach) personalized food images. Intervention group participants always avoid foods that they personally want to eat less often and approach foods that they personally want to eat more often. In the control group, images are paired equally often with both response directions. To evaluate contextual and dynamic intervention effects, ecological momentary assessment (EMA) is measured throughout, with questions about food intake, hunger, stress, emotions, eating intentions, food craving, and impulsivity twice a day. Additional EMA pre- and post-intervention measures are administered before and after the intervention phase (4 days each) with a one-day follow-up EMA four weeks after the intervention. Multilevel models will examine the temporal covariance between approach bias and self-reported variables as well as short- and long-term intervention effects on approach bias, food intake, and craving.

Ethics and dissemination: The study was approved by the Ethics Committee of the University of Salzburg. Results will be published in peer reviewed scientific journals and presented at scientific conferences.

Trial registration: This study was registered at the German Clinical Trials Register DRKS, registration number DRKS00030780, and on the Open Science Framework https://osf.io/yn7kt.

Strengths and limitations of this study

- The study is a randomized controlled trial testing an m-health intervention to assist participants in implementing their dietary intentions through repeated use of a mobile Approach-Avoidance Intervention (AAI) compared with a closely matched active control task
- It includes ecological momentary before, during, and after the trial in both control and intervention groups, which allows examining both short- and long-term intervention effects
- It measures a range of potentially relevant phenomena like food craving, hunger, emotions, stress, and day-level impulsivity
- Control group data allows examining variability in approach bias and its covariation with data obtained through EMA
- Measures of food intake are restricted to single-item daily self-reports which are prone to underreporting and experimenter demand

For peer review only

Humans make several decisions per day about whether, what, and how much to eat [1]. All these decisions have an influence on human health, as overeating can lead to obesity and related diseases [2]. It is therefore important to understand which factors contribute to eating decisions and how we can intervene on them. Traditional psychological models have postulated that people reflect on behavioral options, form intentions, and then translate these into behavior. However, decades of research have shown that intentions are often not successfully enacted, a phenomenon termed the "intention behavior gap" [3].

To investigate why people sometimes fail to transfer their intentions into behavior, researchers have devised a range of indirect measures, typically assessed with computer tasks based on the measurement of reaction times (RT) [4,5], as opposed to direct measures such as self-reported evaluations of stimuli. Under certain conditions, such indirect measures of food preference can increase prediction accuracy of actual behavior, above and beyond questionnaire data [6,7] but see [8] for a critical discussion. One example of such an RT task is the Approach Avoidance Task (AAT). In the AAT, participants usually use a joystick [or in more recent studies, a touch-screen; ,9,10] to perform movements towards or away from different stimulus categories. These categories, such as foods and non-food objects, are compared on how fast they are approached and avoided, and this RT difference is termed the "approach bias" [11]. Herein, we adopt an operational definition, such that we define approach bias as the relative speed with which one can approach the target stimuli (e.g., foods) in the AAT, remaining agnostic to what might be the underlying mental construct [8,12,13]. Food approach biases seem relevant to real-world eating behavior, as they have been found to be higher in people who strongly crave foods [14], and they relate to increased food consumption in impulsive individuals and in people who are prone to external or emotional eating [15,16] (but see [17] for contradictory findings from the alcohol domain and a wider discussion in [18,19]).

So far, it has mostly been ignored that approach biases may fluctuate over time. In most studies to date, approach bias has, at least implicitly, been treated as a relatively stable, trait-like phenomenon, in line with its conceptualization as a (stable) mental construct. Approach bias is typically measured at a single timepoint and then correlated with other phenomena like trait food craving [10], weight status [20], or eating disorder diagnosis [21]. One recent study demonstrated that approach bias was independent of experimentally induced satiety, indicating stability of the bias across situations. However, participants' desire to eat specific foods did explain variance in approach bias, implying that bias might vary across time within individuals depending on their current consumption desires [22]. This is in line with the finding that approach bias for chocolate was positively correlated with current chocolate craving [23–25], craving being an experience of intense desire for a specific food which is temporally variable by definition [26]. Other studies using a mobile version of the AAT indicated that test-retest reliability across eight measurement occasions was low while split-half reliability was high, again indicating temporal fluctuations in approach biases [27]. This is in line with findings obtained from other indirect measures that showed modest stability over time [28]. Such within-subject fluctuations in biases are probably not only due to random variation, as approach-avoidance biases have been shown to decrease with after-meal-satiety in normal-weight individuals, and they have been shown to change based on individuals' current affective states [29–33]. In combination, these results raise questions about the temporal and situational stability of approach biases.

Associations between behavioral approach bias and intake-related variables have led to the development of Approach-Avoidance Interventions (AAI). Traditionally, it has been assumed that a stable mental construct thought to underlie the behavioral approach bias, can be changed by

For peer review only

repeatedly pairing unhealthy foods with avoidance and healthy foods (or neutral objects) with approach. This should then affect food intake. How exactly AAIs might work is however a matter of current debate. Based on the idea that the behavioral approach bias operationalizes learned associations between appetitive stimuli and approach, some authors argue that the repeated pairing of appetitive stimuli and avoidance weakens or reverses this association through the formation of new associations between stimuli, movement direction, and evaluative properties inherent to approach and avoidance [23]. Others however argue that stimulus value is updated due to a conflict between evaluation and within-task behavior which then influences intake decisions [34–36], or that altered behavior is due to changed food evaluations, which are caused by cognitive inferences based on task requirements [37,38]. Independent of what might be the exact working mechanism, earlier studies have shown that the behavioral approach bias [39], food choice [40] and subsequent food intake [41] can be reduced by AAIs. The evidence in this domain is mixed, however [42–46], and might have several different reasons.

Firstly, in some studies participants approach and avoid stimuli based on their category (e.g., food vs objects) and in others, based on an irrelevant feature of the stimulus such as the frame color or orientation. Such task differences may affect participants' awareness of the contingencies between stimulus and required response, as well as expectations about training effects. As this awareness could increase the effectiveness of the intervention, especially when AAIs change behavior through cognitive inferences as noted above [47], this study uses a relevant-feature AAT/AAI and closely tracks participants' contingency awareness. Secondly, the personal relevance of the trained stimuli may differ between interventions, and this may influence effectiveness [48]. Some interventions specifically try to retrain approach biases to chocolate in individuals reporting high trait-level chocolate craving or consumption [39], while other interventions train responses to a pre-selected set of healthy and unhealthy foods without taking into account if participants actually consume the unhealthy foods or if healthy foods fit to individual needs of the participants (for example in terms of taste, food intolerances). Thirdly, most studies only deliver a single session of AAI (with [49,50] being exceptions), while more sessions might lead to larger effects that would be easier to detect [51]. Finally, the effectiveness of AAIs might depend on when the intervention is delivered. It is easy to see that interventions might be most fruitful in or just before moments when the risk for unhealthy intake is high.

Smartphone-based AATs and AAIs are interesting for a range of research questions that cannot be answered with stationary computer-based AATs and AAIs. Firstly, smartphones allow easy delivery of AAI to participants during their daily routines. This helps participants to perform the intervention repeatedly, and to bring the intervention temporally and spatially closer to "high-risk" situations in everyday life. Assuming a rather fast decay of intervention effects, the closer proximity offered by the smartphone should enhance its effectiveness compared to conducting it on one's personal computer [52] or in a laboratory session [53,54]. Another advantage of repeated intervention through smartphones is the possibility to measure immediate and delayed intervention effects on fluctuating phenomena like food craving. Lastly, smartphones allow to measure bias more easily at any time of the day, and especially at moments when it may be relevant for food consumption. This allows us to examine the temporal and situational variability of approach bias. Combining it with repeated delivery of eating-related questions throughout the day (i.e., ecological momentary assessment, EMA) also allows for correlating fluctuations in approach bias with other temporally variable phenomena like food craving, affect and intake.

Several studies have delivered interventions using computer tasks through the internet [52,53,55–57] and have generally reported good compliance rates and effects on dietary intake.

Smartphone-based interventions using similar RT tasks are much rarer and have reported mixed results on key outcomes [49–51,58]. One of the two studies delivering smartphone-based AAI required participants to tilt the phone to respond, and found positive effects on food choice and approach bias towards unhealthy foods [50]. The other study found neither day-level nor longer-term effects of AAI using swipe movements, as compared to an EMA-only intervention and a sham training group [49]. It is important to note that that study did not find any approach bias in participants to begin with which suggests that the swipe movements did not clearly represent approach and avoidance and that the sample size was small. It is therefore unclear to what degree its results can be taken as evidence against the effectiveness of mobile AAI.

One recent AAT variant does not require swiping movements on the touchscreen, but instead requires participants to physically move the phone towards or away from themselves while viewing food stimuli [27,59–61]. This task has been shown to be a valid tool to measure food approach biases outside the laboratory and to provide relevant information beyond self-report measures [59]. In addition to RTs, it also yields data on the force of the movements, which might contain relevant information not captured by RTs [61].

The study presented here sets out to test its effectiveness as an intervention tool for AAI; that is, when it is programmed to pair the foods that a specific participant wants to eat more often with approach, and to pair the foods that a specific participant wants to eat less often with avoidance responses. Specifically, we will study to what degree the intervention can support participants in their goal of changing their eating behavior. We further examine the reliability and validity of approach bias scores obtained through a phone-delivered AAT. Combining the AAI/AAT with the repeated measurement of related phenomena through EMA allows us to disentangle short- and long-term intervention effects as well as to investigate whether approach bias covaries with intake-related variables over time.

METHODS AND ANALYSIS

Study Overview

The study uses a two-arm, double-blind randomized controlled trial conducted with German-speaking participants, and is coordinated at the University of Salzburg, Austria. It compares an active Approach-Avoidance Intervention (AAI) to a sham-training (a measurement Approach-Avoidance Task, AAT) in its impact on eating behavior, food liking, food craving, and food approach bias.

Participants

Participants will be recruited via university e-mailing lists, social networks, university events, and word of mouth. Participants must be between 18 and 60 years of age, and must not be pregnant or report a diagnosis for an eating disorder. Importantly, participants must have an intention to change their eating behavior (which they indicate upon sign-up), without further specification regarding increased or decreased intake of certain foods or food categories.

To determine the required sample size, we performed a power analysis using pre-existing data from a (so far unpublished) study, wherein we attempted to change participants' approach-avoidance bias and analysed its change from pre- to post-training (see Supplementary file 1 for more information). We opted to use this pre-existing data as the structure of the study is similar to the current study and the correlations and noise therein would be more likely to reflect the findings we will observe than would fully simulated data.

For peer review only

First, group-level differences between the pre-to-post effects of sham and active training were removed such that a time-by-group effect size of 0 was achieved. A new effect size was then applied by increasing the post-treatment group mean of the active training participants by a multiple of 16 between 64 and 244, giving effect sizes around g = .5. After this, participants were randomly sampled with replacement such that sample sizes between 80 and 180 in multiples of 10 were achieved. Each combination of sample size and effect size was re-sampled and tested 200 times. After sampling a set of participants, a multilevel analysis was performed where approach-avoidance bias scores were predicted with fixed predictors of treatment group (sham or active) and time (pre-training or post-training), as well as random effects of time grouped by participant and time grouped by stimulus. The p-value of the group by time interaction was recorded. The proportion of p-values below .05 was computed to determine the power at each combination of sample size and effect size.

Based on this power analysis, we determined that a medium effect size (g = .50) and a power of .80 would require a sample size of about valid 150 participants. Based on the effect size observed in that other study of g = 0.56, 150 participants result in a power of about .88. A table with all power analysis outcomes is depicted in Supplementary file 2. With an estimated recruitment rate of three participants per week and allowing for recruitment difficulties slowing down the process, we expect data collection to last from November 2022 to roughly January 2024. Data collection continues until 150 participants are reached.

Materials and procedure

Baseline questionnaires. In a web-based questionnaire (see Supplementary file 3), participants give informed consent (consent form see Supplementary file 4) and then indicate their age, gender, nationality, state of employment, highest achieved formal education level, dietary restrictions (vegan, vegetarian, pescetarian, omnivorous, other), height, weight, and possible food allergies or intolerances. Participants identifying as female or diverse are also asked about their menstrual cycle. Further, to assess exclusion criteria, they are asked if they are currently suffering from an eating disorder. This is followed by the stimulus selection (see below for details). Participants then complete the German versions of the following questionnaires: subscales restrained eating and external eating from the Dutch Eating Behavior Questionnaire [62]; the Salzburg Emotional Eating Scale [63]; the Salzburg Stress Eating Scale [64]; Perceived Self-Regulatory Success in Dieting [65]; the short version of the UPPS Impulsivity Scale [66].

Stimuli. We pre-selected 90 food and drink pictures from the food.pics [67] and CROCUFID [68] databases, and from freely available online resources based on typical availability in Austria and Germany as the main recruitment sites. At the beginning of the study, participants rated these 90 images on two scales: “In the last three weeks, on how many days have you eaten/drunk this food/drink?” (recent intake) and “In the next three weeks, on how many days would you like to eat/drink this food/drink?” (intended intake). We select the six foods with the most negative difference between past and intended consumption as “increase foods” (eaten less often than intended). Then, among the foods that were eaten at least on six days in the past three weeks, we select six with the most positive difference between past and intended future consumption (eaten more often than intended = “decrease-foods”). A randomly selected four of these six images are then used in the intervention phase in both groups while the other two were left untrained to test whether the intervention would be specific to the foods used in the task. A random selection of 8 out of a set of 12 images of office items serves as control stimuli. Figure 1 displays the selection of stimuli. The food stimuli were not categorized as “healthy” or “unhealthy”, giving participants full flexibility for choosing “increase-foods” and “decrease-foods”.

For peer review only

Setup call. Within a few days of filling out the online questionnaire, a member of the study team contacts participants to schedule a setup call via phone or a video conferencing tool. In this call, the member of the research team explains the procedure of the study. They further help participants install the necessary apps onto their smartphone (m-path; KU Leuven, 2022 for EMA and the AAT app [1]) and then confirm the correct selection of approach and avoidance foods as determined by the rating task. After the call, participants receive a manual for the study via e-mail, which summarizes the study procedures and the use of the smartphone applications and includes participants' individual three-digit identification code as well as contact information of the study team.

Approach-Avoidance Task (AAT). We use the smartphone-based AAT as introduced by Zech and colleagues [61]. In this version of the task, participants see stimuli on their horizontally held phone screen and they perform approach or avoidance movements by physically moving the phone towards/away from themselves (see Figure 2 and two short introductory videos here: https://osf.io/4k3q9/?view_only=4db6431fd5ee4148a97f3be7f799ea4a). Each trial starts with a fixation dot in the middle of a white screen, which is followed by either one of the food or object stimuli after a 1500 ms delay. While correct approach or avoidance responses make the picture disappear and trigger the start of a new trial, incorrect responses are followed by a 2000 ms display of a black error-cross. If a participant does not respond for 2000 ms, a clock icon is displayed indicating timeout.

The active and sham AAT trainings feature 4 out of 6 approach-foods, 4 out of 6 avoid-foods, as well as 8 out of 12 control object stimuli. The training sessions consist of four training blocks of 16 trials each, and each training block is preceded by 4 practice trials, yielding a training session of 64 training trials and 16 practice trials, or 80 trials total. The pre-, post- and follow-up bias assessment AATs similarly consist of four blocks preceded each by four practice trials but feature all selected images (6 "increase-foods", 6 "decrease-foods" and 12 objects). All 24 images are presented one time per block, yielding 96 test trials and 16 practice trials, or 112 trials total. In all AATs, the instructions of the blocks alternate such that participants are instructed to approach foods while avoiding objects in the first block (approach-food blocks) and avoid foods while approaching objects in the second block (avoid-food blocks). This order is the same for all sessions and all participants. Crucially, in the active training AATs, only approach-foods are shown in the approach-food blocks, while only avoid-foods are shown in the avoid-food blocks; sham training instead features both approach- and avoid-foods during both approach-food and avoid-food blocks. Completing one session of the AAT/AAI takes about five minutes.

Ecological Momentary Assessment (EMA). Participants follow the EMA schedule for a total of 20 days. During the whole period, participants receive two prompts per day (delivered through the smartphone application m-path [69]), one just before the time a participant usually eats lunch and the other in the evening (prompted at an individualized time agreed-upon with the participant to represent an end-of-day signal). Table 1 shows the questions that participants answer on those prompts and Figure 3 displays the temporal sequence of the study. EMA prompts on days 1-3 of the study only contain the listed questions. On day 4 (the day before the start of the intervention) and day 17 (the day after the end of the intervention), participants receive an instruction to open the AAT application and complete a measurement AAT. On every second day during the intervention phase (days 5 through 16), participants receive an instruction to open the AAT application and complete a training AAT after completing the midday prompt. 30 minutes after completing the midday prompt, participants receive a notification asking whether they conducted the training. On

[1] The app can be downloaded on Android devices from this address: https://play.google.com/store/apps/details?id=com.eatlabsbg.eatapp

replying “yes”, they receive positive feedback; on replying “no”, they are asked to now open the AAT app to conduct the task. The number of sessions was chosen based on earlier, similar studies [50,52], balancing participant burden, compliance, and intervention intensity.

For peer review only

Table 1: overview of EMA questions (own translations from German). All items are answered on a slider from 0 to 100 unless indicated differently in parentheses. Midday prompts remain the same across the entire study duration. Evening prompts differ depending on the study phase as indicated:
* These items are asked alongside a food image and are repeated for each image (six “increase-food” and six “decrease-food” images).
+ This item is only asked after the first and the last AAT session (day 5 and 15).

**Item**	**Midday (all study phases)**	**Evening Days 1-4 and 17-20**	**Evening Days 5-16**	**Evening - follow-up**
When was the last time you ate something?	X			
What type of meal was it? (breakfast, lunch, dinner, snack)	X			
How hungry are you right now?	X	X	X	
Do you feel like you have everything under control?	X			
Do you feel like you are on top of things?	X			
How optimistic do you feel right now?	X			X
How happy do you feel right now?	X			X
How lonely do you feel right now?	X			X
How depressed do you feel right now?	X			X
How angry do you feel right now?	X			X
How mad do you feel right now?	X			X
How tense do you feel right now?	X			X
How anxious do you feel right now?	X			X
How much do you want to stick to your dietary goals *for the rest of the day*?	X			
How much do you want to stick to your dietary goals *tomorrow*?		X		X
How strong has your craving for this food been *today*? *		X		X
How much of this food have you eaten *today*? *		X		X
Have you said things today without thinking?	X	X	X	X

For peer review only

Have you spent more money today than you wanted to?	X	X	X	X
Have you felt impatient today?	X	X	X	X
Have you made a spontaneous decision today?	X	X	X	X
How strong has your craving for this food been *since the midday questionnaire*? *			X	
How much of this food have you eaten *since the midday questionnaire*? *			X	
How much do you expect that this task will help you reach your dietary goals? +	X+			
Throughout the whole study, how often did you push this food away from yourself? *				X
Please indicate the day your last period started.				X

For peer review only

In addition, on day 6 and day 16 (the first and the last day including an AAT/AAI session), participants further indicate their expectancy of how much the task will help them reach their dietary goals. Four weeks after the end of the initial 20-day EMA period, participants receive one additional EMA questionnaire and a measurement AAT in the evening. After performing this final AAT, participants indicate how often they believed they pushed or pulled each of their “decrease-foods” and “increase-foods”.

Procedure. The procedure for study participation is as follows: after interested participants contact the study team, they receive an individual participant code and a weblink to the baseline questionnaire. At this point, an R-script [2] randomizes participants to either the intervention or control group with the condition unknown to the study team. After a setup call with a member of the research team within a few days of filling out the questionnaires, participants start receiving EMA prompts and AAT as described above. Figure 3 shows the timeline of the whole study. Throughout the study period, participants can contact study personnel who also monitor compliance to the EMA and AAT schedule and contact participants in case of low compliance: participants receive an e-mail if they miss more than one of the first three AAT/AAI sessions.

Outcomes

Main outcomes. This study uses three main outcome measures. The first outcome measure is participants’ self-reported intake of “increase” and “decrease” foods according to the EMA schedule outlined above, on a slider from 0 (labelled “nothing”) to 100 (“very much”). The second outcome measure is participants’ self-reported craving for those same foods in the same manner. Simple, single-item measures reduce participant burden but might negatively affect reliability. To ameliorate this, measures are applied for each food separately. Time trends that might indicate changes in participants’ perceptions of amounts as “much” or “little” will be checked in the control group and, if present, controlled for in analyses.

The third outcome measure is the approach bias for all selected foods based on the RT and force in the AAT. The RT is defined as the time from picture onset to movement onset. Force is defined as the peak acceleration in the correct direction during a trial, standardized within participant by dividing every individual’s measurement of force by the participant-specific standard deviation. Separately for approach and avoidance trials as well as for sessions, the RT and the force will be averaged across the four AAT blocks for each specific food stimulus. For objects, we will also average across the different stimuli. The average approach or avoid response for objects on a session will be subtracted from stimulus-specific food approach or avoidance response on that session to achieve food-specific *single-difference* approach and avoidance scores according to these formulas:

Stimulus-specific approach = [food-specific approach]-[average object approach]

Stimulus-specific avoidance = [food-specific avoidance]-[average object avoidance]

Double-difference scores will be used as a full bias score per food stimulus and session, according to the formula: ([food-specific avoidance]-[food-specific approach])-([average object avoidance]-[average object approach]).

---

[2] The function *sample* randomly outputs the number “1” or “2” which correspond to the conditions.

For peer review only

Secondary outcomes. Dietary intentions are measured according to the outlined EMA schedule.

Data analysis plan

Data analysis will serve to investigate a series of research questions relating to different aspects of the study. In this section we will provide a brief description. Full details on the data analysis, including the exact multilevel analysis formulas, are available in the pre-registration at https://osf.io/yn7kt .

Data exclusion. For analyses regarding the effectiveness of the AAI, we exclude participants who did not conduct any of the AATs during the intervention phase, as we regard them as "not treated". For the remaining participants, sensitivity analyses are performed to test whether the number of completed training sessions affects intervention effectiveness. For analyses of approach bias, we exclude error trials and trials with RTs that deviate more than +/-3SD from the individual mean of the participant in that AAT session. If more than 25% of trials must be excluded based on these criteria, the whole AAT session is excluded from further analysis. This post-hoc session exclusion does not affect whether a participant is counted as "not treated" or not in the analyses regarding the effectiveness of the AAI.

Overall intervention effectiveness. The first set of research questions relates to the effectiveness of the intervention as compared to the control condition from pre- to post-training. To this end, we use multilevel models to predict intake of trained "increase" and "decrease" foods as a function of timepoint (three days pre- vs post-intervention), condition (intervention vs control), and their interaction. Equivalent models test the intervention effect on approach biases towards - and craving for - the two food categories. To test to what degree the effect of the training intervention is specific to trained foods, we then use data from trained and untrained stimuli and add a variable that indicates whether a food appeared in the training or not (trained vs untrained) and all interaction terms to the model. This is followed up with tests to determine whether changes in the approach bias are mainly driven by changes in approach- or avoidance RTs. We further test the moderating role of intentions, baseline stimulus craving, and person-level variables obtained from the questionnaires, as well as contingency awareness and expectancy by adding the relevant variable and its interaction terms to the equations. Finally, we examine the mediating effect of craving for changes in intake.

Immediate intervention effectiveness. The second set of research questions concerns the short-term effects of the intervention during the intervention phase (days 5-16). Multilevel models predicting food intake and cravings, respectively, include the factors group (intervention vs control) and (off-)training day (training day vs no training day) as predictors. In another pair of multilevel models, we use group and the number of days since the beginning of the intervention and their interaction as predictors of craving and food intake, respectively. The force applied during the training is used as a predictor for the change in craving and intake from before the start of the training.

Trait and state components of approach bias. The third set of research questions relates to the state and trait components of approach bias and is examined within the control group only. This is because only participants in the control group receive measurement AATs throughout the study period. Multilevel models test whether bias size and negative emotions are related on both a between-subjects and a within-subjects level and to what degree this depends on the strength of the desire for these foods. A separate model tests equivalent research questions for the relation

For peer review only

between bias strength and craving, as well as bias strength and intake, respectively. The latter analyses testing how bias strength is related to subsequent food intake are expanded by including trait and day-level impulsivity and day-level intentions of regulating food intake.

Patient and public involvement

Patients and the public are not involved in study design, data collection, data analysis, or dissemination.

Ethics, dissemination, and data handling.

The study has received ethical approval from the ethics board of the University of Salzburg and is conducted in accordance with the declaration of Helsinki. Results of the trial will be disseminated through a series of articles in appropriate scientific journals and conference presentations.

Data is handled confidentially and stored in a pseudonymized manner. Neither m-path nor the AAT application collect personal data but work through three-digit identification codes assigned to participants. The identification key linking personal data to the identification codes will be kept in password-protected files separately from the pseudonymized data and will be destroyed one year after termination of the study. Deidentified data will be archived for at least ten years and consent forms as documentation of participation will be archived for 30 years. The deidentified data will be made public on the Open Science Framework after the completion of planned publications.

For peer review only

Contributorship statement

MBA: conceptualisation, methodology, writing first draft of the protocol, revising the protocol. HvA: conceptualisation, methodology, analysis plan, reviewing drafts of the protocol. SK: conceptualisation, methodology, analysis plan, reviewing drafts of the protocol. JB: conceptualisation, methodology, supervision, reviewing drafts of the protocol. All authors read and approved the final version of the protocol.

Competing interests

All authors report no competing interests.

Funding

This work was supported by the Austrian Science Fund FWF grant number P 34542-B. Sercan Kahveci and Hannah van Alebeek were supported by the Doctoral College “Imaging the Mind” (FWF; W1233-B). Hannah van Alebeek was additionally supported by the project: Mapping neural mechanisms of appetitive behaviour (FWF; KLI762-B). The funder plays no role in the study design; collection, management, analysis, and interpretation of data; writing of the report; and the decision to submit the report for publication.

For peer review only

References

1 Wansink B, Sobal J. Mindless Eating: The 200 Daily Food Decisions We Overlook. *Environ Behav* 2007;**39**:106–23. doi:10.1177/0013916506295573

2 World Health Organization. Obesity and Overweight. Published Online First: 2018. doi:10.1007/978-3-319-33228-4_447

3 Sheeran P, Webb TL. The Intention–Behavior Gap. *Soc Personal Psychol Compass* 2016;**10**:503–18. doi:10.1111/spc3.12265

4 Gawronski B, De Houwer J. Implicit measures in social and personality psychology. In: *Handbook of research methods in social and personality psychology*. Cambridge University Press 2014. 283–310.http://hdl.handle.net/1854/LU-2959505 (accessed 29 Apr 2020).

5 Gawronski B, Hahn A. IMPLICIT MEASURES Procedures, Use, and Interpretation. In: Blanton H, LaCroix JM, Webster GD, eds. *Measurement in Social Psychology*. New York, NY: : Yaylor & Francis 2019. 29–55.http://www.bertramgawronski.com/documents/GH2019Measurement.pdf

6 Hagger MS, Trost N, Keech J, *et al.* Predicting sugar consumption: Application of an integrated dual-process, dual-phase model. *Appetite* Published Online First: 2017. doi:10.1016/j.appet.2017.04.032

7 Hofmann W, Friese M, Wiers RW. Impulsive versus reflective influences on health behavior: a theoretical framework and empirical review. *Health Psychol Rev* 2008;**2**:111–37. doi:10.1080/17437190802617668

8 Van Dessel P, Cummins J, Hughes S, *et al.* Reflecting on 25 Years of Research Using Implicit Measures: Recommendations for Their Future Use. *Soc Cogn* 2020;**38**:s223–42. doi:10.1521/soco.2020.38.supp.s223

9 Kahveci S, van Alebeek H, Berking M, *et al.* Touchscreen-based assessment of food approach biases: investigating reliability and item-specific preferences. *Appetite* 2021;:105190. doi:10.1016/j.appet.2021.105190

10 Meule A, Lender A, Richard A, *et al.* Approach–avoidance tendencies towards food: Measurement on a touchscreen and the role of attention and food craving. *Appetite* 2019;**137**:145–51.

11 Rinck M, Becker ES. Approach and avoidance in fear of spiders. *J Behav Ther Exp Psychiatry* 2007;**38**:105–20. doi:10.1016/j.jbtep.2006.10.001

12 Schimmack U. The Implicit Association Test: A Method in Search of a Construct. *Perspect Psychol Sci* 2019;:1745691619863798. doi:10.1177/1745691619863798

13 De Houwer J. Implicit Bias Is Behavior: A Functional-Cognitive Perspective on Implicit Bias. *Perspect Psychol Sci* 2019;:1745691619855638. doi:10.1177/1745691619855638

14 Brockmeyer T, Hahn C, Reetz C, *et al.* Approach bias and cue reactivity towards food in people with high versus low levels of food craving. *Appetite* 2015;**95**:197–202. doi:10.1016/j.appet.2015.07.013

15 Kakoschke N, Kemps E, Tiggemann M. Impulsivity moderates the effect of approach bias modification on healthy food consumption. *Appetite* 2017;**117**:117–25. doi:10.1016/j.appet.2017.06.019

16 Kakoschke N, Kemps E, Tiggemann M. Differential effects of approach bias and eating style on unhealthy food consumption in overweight and normal weight women. *Psychol Health* 2017;**32**:1371–85. doi:10.1080/08870446.2017.1327587

17 Spruyt A, De Houwer J, Tibboel H, *et al.* On the predictive validity of automatically activated approach/avoidance tendencies in abstaining alcohol-dependent patients. *Drug Alcohol Depend* 2013;**127**:81–6. doi:10.1016/j.drugalcdep.2012.06.019

18 Friese M, Hofmann W, Schmitt M. When and why do implicit measures predict behaviour? Empirical evidence for the moderating role of opportunity, motivation, and process reliance. *Eur Rev Soc Psychol* 2008;**19**:285–338. doi:10.1080/10463280802556958

19 Kakoschke N, Albertella L, Lee RSC, *et al.* Assessment of Automatically Activated Approach–Avoidance Biases Across Appetitive Substances. *Curr Addict Rep* 2019;:1–10. doi:10.1007/s40429-019-00254-2

20 Kemps E, Tiggemann M. Approach Bias for Food Cues in Obese Individuals. *Psychol Health* 2014;**0446**:1–25. doi:10.1080/08870446.2014.974605

21 Neimeijer RAM, de Jong PJ, Roefs A. Automatic approach/avoidance tendencies towards food and the course of anorexia nervosa. *Appetite* 2015;**91**:28–34. doi:http://dx.doi.org/10.1016/j.appet.2015.03.018

22 Kahveci S, Meule A, Lender A, *et al.* Food approach bias is moderated by the desire to eat specific foods. *Appetite* 2020;**154**:104758. doi:10.1016/j.appet.2020.104758

23 Kemps E, Tiggemann M, Martin R, *et al.* Implicit approach-avoidance associations for craved food cues. *J Exp Psychol Appl* 2013;**19**:30–8. doi:10.1037/a0031626

For peer review only

24 Lender A, Meule A, Rinck M, *et al.* Measurement of food-related approach–avoidance biases: Larger biases when food stimuli are task relevant. *Appetite* 2018;**125**:42–7. doi:10.1016/j.appet.2018.01.032

25 Wittekind CE, Blechert J, Schiebel T, *et al.* Comparison of different response devices to assess behavioral tendencies towards chocolate in the approach-avoidance task. *Appetite* 2021;**165**:105294. doi:10.1016/j.appet.2021.105294

26 Weingarten HP, Elston D. The phenomenology of food cravings. *Appetite* 1990;**15**:231–46.

27 Zech HG, Gable P, van Dijk WW, *et al.* Test-retest reliability of a smartphone-based approach-avoidance task: Effects of retest period, stimulus type, and demographics. *Behav Res Methods* Published Online First: 1 August 2022. doi:10.3758/s13428-022-01920-6

28 Gawronski B, Morrison M, Phills CE, *et al.* Temporal Stability of Implicit and Explicit Measures: A Longitudinal Analysis. *Pers Soc Psychol Bull* 2017;**43**:300–12. doi:10.1177/0146167216684131

29 Krehbiel J, Halbeisen G, Kühn S, *et al.* Too hot to handle: Mood states moderate implicit approach vs. avoidance tendencies toward food cues in patients with obesity and active binge eating disorder. *J Psychiatr Res* 2021;**143**:302–8. doi:10.1016/j.jpsychires.2021.09.031

30 Neimeijer RAM, Roefs A, Ostafin BD, *et al.* Automatic Approach Tendencies toward High and Low Caloric Food in Restrained Eaters: Influence of Task-Relevance and Mood. *Front Psychol* 2017;**8**.https://www.frontiersin.org/articles/10.3389/fpsyg.2017.00525 (accessed 24 Oct 2022).

31 Richard A, Meule A, Blechert J. When and how do explicit measures of food craving predict implicit food evaluation? A moderated mediation model. *Food Qual Prefer* 2018;**66**:141–7. doi:10.1016/J.FOODQUAL.2018.01.018

32 Richard A, Meule A, Blechert J. Implicit evaluation of chocolate and motivational need states interact in predicting chocolate intake in everyday life. *Eat Behav* 2019;**33**:1–6. doi:10.1016/j.eatbeh.2019.01.006

33 Zech HG. Food approach dynamics in daily live: Speed and force of food approach movements fluctuate with hunger, but less so for people with high BMI. 2022.

34 Veling H, Holland RW, van Knippenberg A. When approach motivation and behavioral inhibition collide: Behavior regulation through stimulus devaluation. *J Exp Soc Psychol* 2008;**44**:1013–9. doi:10.1016/j.jesp.2008.03.004

35 Veling H, Becker D, Liu H, *et al.* How go/no-go training changes behavior: A value-based decision-making perspective. *Curr Opin Behav Sci* 2022;**47**:101206. doi:10.1016/j.cobeha.2022.101206

36 Houben K, Aulbach M. Is there a difference between stopping and avoiding? A review of the mechanisms underlying Go/No-Go and Approach-Avoidance training for food choice. *Curr Opin Behav Sci* 2023;**49**.

37 Van Dessel P, Gawronski B, Smith CT, *et al.* Mechanisms underlying approach-avoidance instruction effects on implicit evaluation: Results of a preregistered adversarial collaboration. *J Exp Soc Psychol* 2017;**69**:23–32. doi:10.1016/J.JESP.2016.10.004

For peer review only

38 Van Dessel P, Hughes S, De Houwer J. How Do Actions Influence Attitudes? An Inferential Account of the Impact of Action Performance on Stimulus Evaluation. *Personal Soc Psychol Rev* 2019;**23**:267–84. doi:10/gf5sps

39 Dickson H, Kavanagh DJDJ, MacLeod C. The pulling power of chocolate: Effects of approach-avoidance training on approach bias and consumption. *Appetite* 2016;**99**:46–51. doi:10.1016/j.appet.2015.12.026

40 Veling H, Verpaalen IAM, Liu H, *et al.* How can food choice best be trained? Approach-avoidance versus go/no-go training. *Appetite* 2021;:105226. doi:10/gjkp4t

41 Schumacher SE, Kemps E, Tiggemann M. Bias modification training can alter approach bias and chocolate consumption. *Appetite* 2016;**96**:219–24. doi:10.1016/j.appet.2015.09.014

42 Aulbach MB, Knittle K, Haukkala A. Implicit process interventions in eating behaviour: a meta-analysis examining mediators and moderators. *Health Psychol Rev* 2019;**13**:179–208. doi:10.1080/17437199.2019.1571933

43 Becker D, Jostmann NB, Wiers RW, *et al.* Approach avoidance training in the eating domain: Testing the effectiveness across three single session studies. *Appetite* 2015;**85**:58–65. doi:10.1016/j.appet.2014.11.017

44 Becker D, Jostmann NB, Holland RW. *Does approach bias modification really work in the eating domain? A commentary on Kakoschke et al. (2017)*. 2018. doi:10.1016/j.addbeh.2017.02.025

45 Navas JF, Verdejo-García A, Vadillo MA. The evidential value of research on cognitive training to change food-related biases and unhealthy eating behavior: A systematic review and p-curve analysis. *Obes Rev* 2021;**22**:e13338. doi:10.1111/obr.13338

46 Yang Y, Shields GS, Wu Q, *et al.* Cognitive training on eating behaviour and weight loss: A meta-analysis and systematic review. *Obes Rev* 2019;**0**. doi:10.1111/obr.12916

47 Van Dessel P, De Houwer J, Gast A. Approach–Avoidance Training Effects Are Moderated by Awareness of Stimulus–Action Contingencies. *Pers Soc Psychol Bull* 2016;**42**:81–93. doi:10.1177/0146167215615335

48 Wiers RW, Van Dessel P, Köpetz C. ABC Training: A New Theory-Based Form of Cognitive-Bias Modification to Foster Automatization of Alternative Choices in the Treatment of Addiction and Related Disorders. *Curr Dir Psychol Sci* 2020;**29**:499–505. doi:10/ghfdf3

49 Meule A, Richard A, Dinic R, *et al.* Effects of a Smartphone-Based Approach-Avoidance Intervention on Chocolate Craving and Consumption: Randomized Controlled Trial. *JMIR MHealth UHealth* 2019;**7**:e12298. doi:10.2196/12298

50 Kakoschke N, Hawker C, Castine B, *et al.* Smartphone-based cognitive bias modification training improves healthy food choice in obesity: A pilot study. *Eur Eat Disord Rev* 2018;**26**:526–32. doi:10.1002/erv.2622

51 Aulbach MB, Knittle K, van Beurden SB, *et al.* App-based food Go/No-Go training: User engagement and dietary intake in an opportunistic observational study. *Appetite* 2021;**165**:105315. doi:10/gmvjtq

For peer review only

52 Lawrence NS, O'Sullivan J, Parslow D, *et al.* Training response inhibition to food is associated with weight loss and reduced energy intake. *Appetite* 2015;**95**:17–28. doi:10.1016/j.appet.2015.06.009

53 Stice E, Yokum S, Veling H, *et al.* Pilot test of a novel food response and attention training treatment for obesity: Brain imaging data suggest actions shape valuation. *Behav Res Ther* 2017;**94**:60–70. doi:10.1016/j.brat.2017.04.007

54 Stice E, Yokum S, Gau J, *et al.* Efficacy of a food response and attention training treatment for obesity: A randomized placebo controlled trial. *Behav Res Ther* 2022;**158**:104183. doi:10.1016/j.brat.2022.104183

55 Forman EM, Manasse SM, Dallal DH, *et al.* Computerized neurocognitive training for improving dietary health and facilitating weight loss. *J Behav Med* Published Online First: 2019. doi:10.1007/s10865-019-00024-5

56 Poppelaars A, Scholten H, Granic I, *et al.* When winning is losing: A randomized controlled trial testing a video game to train food-specific inhibitory control. *Appetite* 2018;**129**:143–54. doi:10.1016/j.appet.2018.06.039

57 Veling H, van Koningsbruggen GMGM, Aarts H, *et al.* Targeting impulsive processes of eating behavior via the internet. Effects on body weight. *Appetite* 2014;**78**:102–9. doi:10.1016/j.appet.2014.03.014

58 Keeler JL, Chami R, Cardi V, *et al.* App-based food-specific inhibitory control training as an adjunct to treatment as usual in binge-type eating disorders: A feasibility trial. *Appetite* 2022;**168**:105788.

59 Brouwer A-M, van Beers JJ, Sabu P, *et al.* Measuring Implicit Approach–Avoidance Tendencies towards Food Using a Mobile Phone outside the Lab. *Foods* 2021;**10**:1440. doi:10.3390/foods10071440

60 van Beers JJ, Kaneko D, Stuldreher IV, *et al.* An Accessible Tool to Measure Implicit Approach-Avoidance Tendencies Towards Food Outside the Lab. In: *Companion Publication of the 2020 International Conference on Multimodal Interaction*. Virtual Event Netherlands: : ACM 2020. 307–11. doi:10.1145/3395035.3425647

61 Zech HG, Rotteveel M, van Dijk WW, *et al.* A mobile approach-avoidance task. *Behav Res Methods* 2020;**52**:2085–97. doi:10.3758/s13428-020-01379-3

62 van Strien T, Frijters JER, Bergers GPA, *et al.* The Dutch Eating Behavior Questionnaire (DEBQ) for assessment of restrained, emotional, and external eating behavior. *Int J Eat Disord* 1986;**5**:295–315. doi:10.1002/1098-108X(198602)5:2<295::AID-EAT2260050209>3.0.CO;2-T

63 Meule A, Reichenberger J, Blechert J. Development and Preliminary Validation of the Salzburg Emotional Eating Scale. *Front Psychol* 2018;**9**.https://www.frontiersin.org/articles/10.3389/fpsyg.2018.00088 (accessed 18 Oct 2022).

64 Meule A, Reichenberger J, Blechert J. Development and preliminary validation of the Salzburg Stress Eating Scale. *Appetite* 2018;**120**:442–8. doi:10.1016/j.appet.2017.10.003

For peer review only

65 Meule A, Papies EK, Kübler A. Differentiating between successful and unsuccessful dieters. Validity and reliability of the Perceived Self-Regulatory Success in Dieting Scale. *Appetite* 2012;**58**:822–6. doi:10.1016/j.appet.2012.01.028

66 Whiteside SP, Lynam DR, Miller JD, *et al.* Validation of the UPPS impulsive behaviour scale: a four-factor model of impulsivity. *Eur J Personal* 2005;**19**:559–74.

67 Blechert J, Lender A, Polk S, *et al.* Food-Pics_Extended—An Image Database for Experimental Research on Eating and Appetite: Additional Images, Normative Ratings and an Updated Review. *Front Psychol* 2019;**10**:307. doi:10.3389/fpsyg.2019.00307

68 Toet A, Kaneko D, de Kruijf I, *et al.* CROCUFID: A Cross-Cultural Food Image Database for Research on Food Elicited Affective Responses. *Front Psychol* 2019;**10**:58. doi:10.3389/fpsyg.2019.00058

69 KU Leuven. m-Path – Free tool for blended care by KU Leuven. 2022.https://m-path.io/landing/ (accessed 18 Oct 2022).

For peer review only

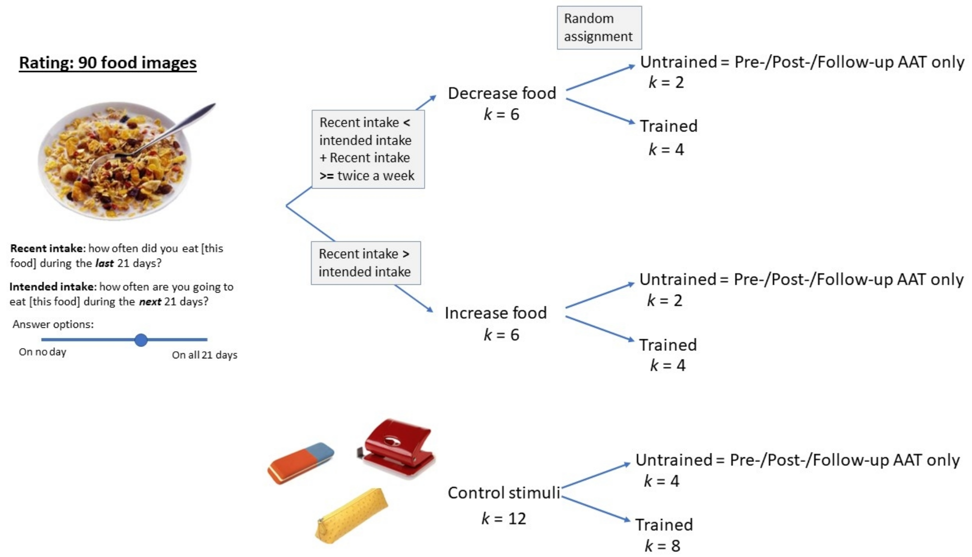

Selection of food personal food stimuli.

338x190mm (96 x 96 DPI)

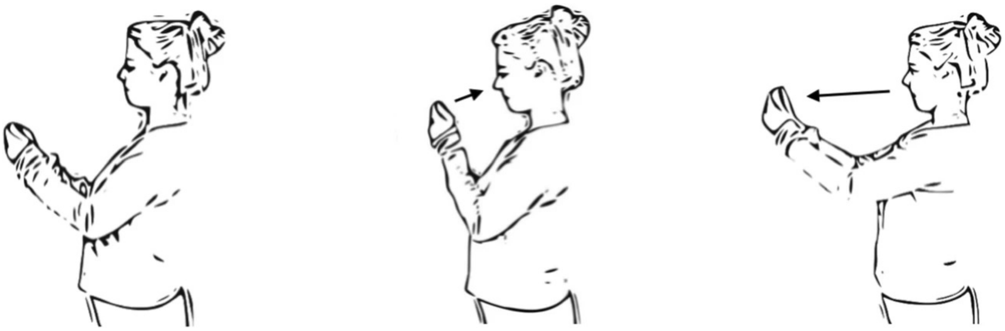

Illustration of the task. On approach trials, participants move the phone closer to themselves (central image), on avoidance trials, they move it away from themselves (Figure reproduced from Zech et al., 2020).

274x89mm (120 x 120 DPI)

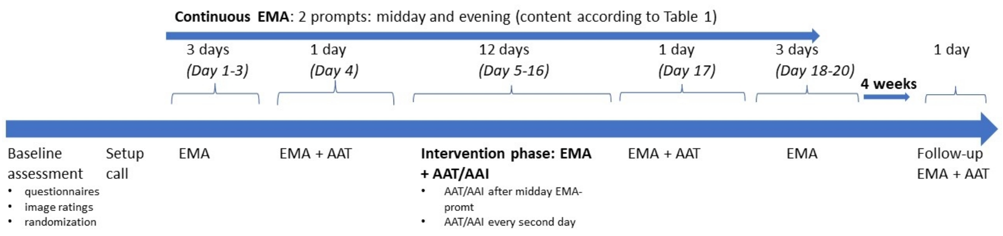

Time schedule of the whole study period.

338x110mm (96 x 96 DPI)

Supplementary material for the manuscript "Study protocol for a Randomized-Controlled Trial to Test the Effectiveness of a Mobile Approach Avoidance Intervention and to Measure Approach Biases in an EMA context"

In this study, 219 valid German-speaking participants performed a single-session online approach-avoidance training where they were trained to avoid chocolate and approach fruit; their approach biases towards chocolate and fruit were measured before and after. Participants performed one of two variants of the AAT, both as a training and as an approach bias measurement. The irrelevant-feature AAT involved moving stimuli towards or away from oneself on the basis of the shape of a frame around them. The dual-feature AAT involved moving stimuli towards or away from oneself on the basis of the combination of stimulus content and frame type, such that one of the frames meant that foods were approached and objects avoided, while the other frame meant that foods were avoided and objects approached. Half of all participants in each training group received active training, and the other half received sham training. The pre- and post-training approach bias measurements each included 112 trials, of which half involved trained stimuli and half untrained stimuli, and in which all stimulus categories were approached and avoided equally often. The training involved 224 trials, of which 64 featured fruit stimuli, 64 featured chocolate stimuli, and 96 featured object stimuli. Control stimuli were always approached and avoided equally often. In the sham trainings, chocolate and fruit stimuli were approached and avoided equally often as well (32 times each). In the active training, chocolate stimuli were approached 56 times and avoided 8 times; vice versa for fruit stimuli. Our power analysis focused on the effect of the dual-feature AAT training on approach biases towards trained fruit stimuli. This training included 52 participants in the active training condition and 55 participants in the sham training condition.

For peer review only

Supplementary Table 1: Statistical power for a range of effect sizes and sample sizes.

Sample size	*Effect size (Hedges' g)*										
	.28	*.36*	*.43*	*.5*	*.57*	*.65*	*.72*	*.79*	*.86*	*.93*	*1.01*
80	.19	.28	.38	.52	.64	.72	.82	.90	.94	.98	.98
90	.24	.34	.43	.58	.70	.76	.89	.94	.96	.96	.99
100	.17	.32	.46	.65	.69	.83	.86	.94	.98	.98	1
110	.24	.42	.57	.66	.82	.85	.92	.97	.99	1	1
120	.23	.41	.62	.66	.81	.89	.94	.97	1	1	.99
130	.34	.36	.62	.78	.86	.90	.97	.99	.99	1	.99
140	.32	.49	.56	.77	.88	.97	.97	.99	1	.99	1
150	.36	.52	.63	.78	.88	.94	.97	1	1	1	1
160	.35	.57	.68	.82	.90	.96	.98	.99	.99	1	1
170	.41	.54	.72	.83	.92	.97	.98	.99	1	1	1
180	.37	.54	.70	.90	.92	.98	.99	.99	1	1	1

For peer review only

Teil A: Zahlencode

A1. Bitte geben Sie hier Ihren Zahlencode aus der Mail ein:

Teil B: Demografische Variablen

Bitte beantworten Sie zunächst einige Fragen zu Ihrer Person.

Leider keine Teilnahme möglich

Eine der Apps, die für die Studie benötigt wird, funktioniert momentan nur mit Android-Betriebssystemen. Deshalb können Sie leider ohne ein entsprechendes Smartphone nicht an der Studie teilnehmen. Falls Sie an einer anderen psychologischen Studie teilnehmen wollen, finden Sie eine Studienliste unserer Abteilung auf dieser Seite: https://sites.google.com/site/eatingandanxietylab/mitmachen

Sie können diesen Fragebogen jetzt schließen.

Leider keine Teilnahme möglich

Leider erfüllen Sie nicht die Einschlusskriterien für diese Studie. Falls Sie an einer anderen psychologischen Studie teilnehmen wollen, finden Sie eine Studienliste unserer Abteilung auf dieser Seite: https://sites.google.com/site/eatingandanxietylab/mitmachen

Sie können diesen Fragebogen jetzt schließen.

Leider keine Teilnahme möglich

Leider erfüllen Sie nicht die Einschlusskriterien für diese Studie. Falls Sie an einer anderen psychologischen Studie teilnehmen wollen, finden Sie eine Studienliste unserer Abteilung auf dieser Seite: https://sites.google.com/site/eatingandanxietylab/mitmachen

Sie können diesen Fragebogen jetzt schließen.

B1. Um an der Studie teilnehmen zu können, benötigen Sie ein Smartphone mit einem Android-Betriebssystem. Bitte geben Sie daher hier die auf Sie passende Antwort an:

- Ich habe ein Smartphone mit Android-Betriebssystem zur Verfügung (ein eigenes, oder für die Dauer der Studie aus meinem Umfeld organisiert). ☐
- Ich habe kein Smartphone mit Android-Betriebssystem, wäre aber bereit, mir für die Studie eines von der Uni Salzburg auszuleihen. ☐
- Ich habe kein Smartphone mit Android-Betriebssystem zur Verfügung und wäre nicht bereit, mir für die Studie eines von der Uni Salzburg auszuleihen. ☐

B2. Bitte geben Sie Ihr Alter an.

B3. **Bitte geben Sie Ihr Geschlecht an.**

- weiblich ☐
- männlich ☐
- divers ☐

B4. **Sind Sie schwanger?**

- Ja ☐
- Nein ☐

B5. **Bitte wählen Sie alle auf Sie zutreffenden Optionen bezüglich Ihres Menstruationszyklus und/oder das von Ihnen aktuell verwendete Verhütungsmittel aus.**

- Trifft nicht zu ☐
- Natürlicher Menstruationszyklus ☐
- Verwendung der Kupferspirale, Kupferkette, Goldspirale ☐
- Verwendung der Hormonspirale ☐
- Verwendung der Pille ☐
- Verwendung der Pille, die IMMER eingenommen wird ☐
- Dreimonatsspritze ☐
- Hormonimplantat ☐
- Verhütungspflaster ☐
- Hormonersatztherapie ☐
- Menopause ☐

Sonstiges

Sonstiges

B6. **Bitte geben Sie den Starttag Ihrer letzten Menstruation an.**

B7. **Bitte geben Sie den Starttag Ihrer vorletzten Menstruation an.**

B8. **Bitte geben Sie den Starttag Ihrer drittletzten Menstruation an.**

B9. **Bitte tragen Sie Ihre Nationalität ein.**

B10. Was ist Ihr höchster erreichter Bildungsabschluss?

- Ohne Schulabschluss ☐
- Hauptschulabschluss ☐
- abgeschlossene Lehre ☐
- mittlere Reife ☐
- Abitur / Matura ☐
- abgeschlossenes Bachelorstudium ☐
- abgeschlossenes Masterstudium ☐
- Sonstiges ☐

Sonstiges

B11. Welcher Berufsgruppe gehören Sie an?

- Student/in ☐
- Auszubildende/r ☐
- Schüler/in ☐
- Angestellte/r ☐
- Selbstständige/r ☐
- Sonstiges ☐

Sonstiges

B12. Wie ernähren Sie sich?

- Vegan (Ernährung ohne von Tieren stammenden Nahrungsmittel) ☐
- Vegetarisch (fleisch- und fischfreie Ernährung) ☐
- Pescetarisch (fleisch- aber nicht fischfreie Ernährung) ☐
- Omnivor (kein Nahrungsmittelverzicht) ☐
- Sonstiges ☐

Sonstiges

B13. **Bitte geben Sie Ihre aktuelle Körpergröße an.**

Angabe in Zentimetern.

B14. **Bitte geben Sie Ihr aktuelles Gewicht an.**

Angabe in Kilogramm.

B15. **Haben Sie eine Nahrungsmittelunverträglichkeit?**

Ja ☐

Nein ☐

B16. **Welche Nahrungsmittelunverträglichkeit haben Sie?**

B17. **Haben Sie aktuell (d.h. innerhalb der letzten 12 Wochen) eine diagnostizierte Essstörung?**

Ja ☐

Nein ☐

Teil C: Konsumziele 1/9

Auf den nächsten 9 Seiten bekommen Sie Bilder von insgesamt 90 Nahrungsmitteln angezeigt und die Aufforderung, jeweils Ihren vergangenen und zukünftigen Konsum der abgebildeten Nahrungsmittel anzugeben. Die Anpassung der Studie auf Ihre Ernährungsziele erfolgt aufgrund Ihrer Antworten in diesem Teil des Fragebogens. Für eine möglichst gute Individualisierung ist es daher wichtig, dass Sie die Fragen möglichst genau beantworten.

C1. **Erdnussflips**

An wie vielen Tagen haben Sie in den letzten drei Wochen Erdnussflips gegessen?|an keinem Tag|an allen 21 Tagen

An wie vielen Tagen wollen Sie in den nächsten drei Wochen Erdnussflips essen?|an keinem Tag|an allen 21 Tagen

C2. **gemischtes Müsli**

An wie vielen Tagen haben Sie in den letzten drei Wochen gemischtes Müsli gegessen?|an keinem Tag|an allen 21 Tagen

An wie vielen Tagen wollen Sie in den nächsten drei Wochen gemischtes Müsli essen?|an keinem Tag|an allen 21 Tagen

C3. **Nussmischung**

An wie vielen Tagen haben Sie in den letzten drei Wochen Studentenfutter gegessen?|an keinem Tag|an allen 21 Tagen

An wie vielen Tagen wollen Sie in den nächsten drei Wochen Studentenfutter essen?|an keinem Tag|an allen 21 Tagen

C4. **Fisch**

An wie vielen Tagen haben Sie in den letzten drei Wochen Fisch gegessen?|an keinem Tag|an allen 21 Tagen

An wie vielen Tagen wollen Sie in den nächsten drei Wochen Fisch essen?|an keinem Tag|an allen 21 Tagen

C5. **salziger Snackmix**

An wie vielen Tagen haben Sie in den letzten drei Wochen salzigen Snackmix gegessen?|an keinem Tag|an allen 21 Tagen

An wie vielen Tagen wollen Sie in den nächsten drei Wochen salzigen Snackmix essen?|an keinem Tag|an allen 21 Tagen

C6. **Pizza**

An wie vielen Tagen haben Sie in den letzten drei Wochen Pizza gegessen?|an keinem Tag|an allen 21 Tagen

An wie vielen Tagen wollen Sie in den nächsten drei Wochen Pizza essen?|an keinem Tag|an allen 21 Tagen

C7. Honig

An wie vielen Tagen haben Sie in den letzten drei Wochen Honig gegessen?|an keinem Tag|an allen 21 Tagen

An wie vielen Tagen wollen Sie in den nächsten drei Wochen Honig essen?|an keinem Tag|an allen 21 Tagen

C8. Chicken Nuggets

An wie vielen Tagen haben Sie in den letzten drei Wochen Chicken Nuggets gegessen?|an keinem Tag|an allen 21 Tagen

An wie vielen Tagen wollen Sie in den nächsten drei Wochen Chicken Nuggets essen?|an keinem Tag|an allen 21 Tagen

C9. Tofu

An wie vielen Tagen haben Sie in den letzten drei Wochen Tofu gegessen?|an keinem Tag|an allen 21 Tagen

An wie vielen Tagen wollen Sie in den nächsten drei Wochen Tofu essen?|an keinem Tag|an allen 21 Tagen

C10. Paprika

An wie vielen Tagen haben Sie in den letzten drei Wochen Paprika gegessen?|an keinem Tag|an allen 21 Tagen

An wie vielen Tagen wollen Sie in den nächsten drei Wochen Paprika essen?|an keinem Tag|an allen 21 Tagen

Teil D: Konsumziele 2/9

D1. Tee

An wie vielen Tagen haben Sie in den letzten drei Wochen Tee getrunken?|an keinem Tag|an allen 21 Tagen

An wie vielen Tagen wollen Sie in den nächsten drei Wochen Tee trinken?|an keinem Tag|an allen 21 Tagen

D2. Fischstäbchen

An wie vielen Tagen haben Sie in den letzten drei Wochen Fischstäbchen gegessen?|an keinem Tag|an allen 21 Tagen

An wie vielen Tagen wollen Sie in den nächsten drei Wochen Fischstäbchen essen?|an keinem Tag|an allen 21 Tagen

D3. Pilze

An wie vielen Tagen haben Sie in den letzten drei Wochen Pilze gegessen?|an keinem Tag|an allen 21 Tagen

An wie vielen Tagen wollen Sie in den nächsten drei Wochen Pilze essen?|an keinem Tag|an allen 21 Tagen

D4. salziges Gebäck

An wie vielen Tagen haben Sie in den letzten drei Wochen salziges Gebäck gegessen?|an keinem Tag|an allen 21 Tagen

An wie vielen Tagen wollen Sie in den nächsten drei Wochen salziges Gebäck essen?|an keinem Tag|an allen 21 Tagen

D5. **Gurke**

An wie vielen Tagen haben Sie in den letzten drei Wochen Gurke gegessen?|an keinem Tag|an allen 21 Tagen

An wie vielen Tagen wollen Sie in den nächsten drei Wochen Gurke essen?|an keinem Tag|an allen 21 Tagen

D6. **Quinoa**

An wie vielen Tagen haben Sie in den letzten drei Wochen Quinoa gegessen?|an keinem Tag|an allen 21 Tagen

An wie vielen Tagen wollen Sie in den nächsten drei Wochen Quinoa essen?|an keinem Tag|an allen 21 Tagen

D7. **Kartoffelbrei**

An wie vielen Tagen haben Sie in den letzten drei Wochen Kartoffelbrei gegessen?|an keinem Tag|an allen 21 Tagen

An wie vielen Tagen wollen Sie in den nächsten drei Wochen Kartoffelbrei essen?|an keinem Tag|an allen 21 Tagen

D8. **Schokonüsse**

An wie vielen Tagen haben Sie in den letzten drei Wochen Schokonüsse gegessen?|an keinem Tag|an allen 21 Tagen

An wie vielen Tagen wollen Sie in den nächsten drei Wochen Schokonüsse essen?|an keinem Tag|an allen 21 Tagen

D9. **Hummus**

An wie vielen Tagen haben Sie in den letzten drei Wochen Hummus gegessen?|an keinem Tag|an allen 21 Tagen

An wie vielen Tagen wollen Sie in den nächsten drei Wochen Hummus essen?|an keinem Tag|an allen 21 Tagen

D10. Kiwi

An wie vielen Tagen haben Sie in den letzten drei Wochen Kiwi gegessen?|an keinem Tag|an allen 21 Tagen

An wie vielen Tagen wollen Sie in den nächsten drei Wochen Kiwi essen?|an keinem Tag|an allen 21 Tagen

Teil E: Konsumziele 3/9

E1. Lasagne

An wie vielen Tagen haben Sie in den letzten drei Wochen Lasagne gegessen?|an keinem Tag|an allen 21 Tagen

An wie vielen Tagen wollen Sie in den nächsten drei Wochen Lasagne essen?|an keinem Tag|an allen 21 Tagen

E2. Nüsse im Teigmantel

An wie vielen Tagen haben Sie in den letzten drei Wochen Nüsse im Teigmantel gegessen?|an keinem Tag|an allen 21 Tagen

An wie vielen Tagen wollen Sie in den nächsten drei Wochen Nüsse im Teigmantel essen?|an keinem Tag|an allen 21 Tagen

E3. Zucchini

An wie vielen Tagen haben Sie in den letzten drei Wochen Zucchini gegessen?|an keinem Tag|an allen 21 Tagen

An wie vielen Tagen wollen Sie in den nächsten drei Wochen Zucchini essen?|an keinem Tag|an allen 21 Tagen

E4. Wassereis

An wie vielen Tagen haben Sie in den letzten drei Wochen Wassereis gegessen?|an keinem Tag|an allen 21 Tagen

An wie vielen Tagen wollen Sie in den nächsten drei Wochen Wassereis essen?|an keinem Tag|an allen 21 Tagen

E5. Linsen

An wie vielen Tagen haben Sie in den letzten drei Wochen Linsen gegessen?|an keinem Tag|an allen 21 Tagen

An wie vielen Tagen wollen Sie in den nächsten drei Wochen Linsen essen?|an keinem Tag|an allen 21 Tagen

E6. Avocado

An wie vielen Tagen haben Sie in den letzten drei Wochen Avocado gegessen?|an keinem Tag|an allen 21 Tagen

An wie vielen Tagen wollen Sie in den nächsten drei Wochen Avocado essen?|an keinem Tag|an allen 21 Tagen

E7. Möhren

An wie vielen Tagen haben Sie in den letzten drei Wochen Möhren gegessen?|an keinem Tag|an allen 21 Tagen

An wie vielen Tagen wollen Sie in den nächsten drei Wochen Möhren essen?|an keinem Tag|an allen 21 Tagen

E8. Cappuccino

An wie vielen Tagen haben Sie in den letzten drei Wochen Cappuccino getrunken?|an keinem Tag|an allen 21 Tagen

An wie vielen Tagen wollen Sie in den nächsten drei Wochen Cappuccino trinken?|an keinem Tag|an allen 21 Tagen

E9. Salzcracker

An wie vielen Tagen haben Sie in den letzten drei Wochen Cracker gegessen?|an keinem Tag|an allen 21 Tagen

An wie vielen Tagen wollen Sie in den nächsten drei Wochen Cracker essen?|an keinem Tag|an allen 21 Tagen

E10. Milch

An wie vielen Tagen haben Sie in den letzten drei Wochen Milch getrunken?|an keinem Tag|an allen 21 Tagen

An wie vielen Tagen wollen Sie in den nächsten drei Wochen Milch trinken?|an keinem Tag|an allen 21 Tagen

Teil F: Konsumziele 4/9

F1. Karamellriegel

An wie vielen Tagen haben Sie in den letzten drei Wochen Karamellriegel gegessen?|an keinem Tag|an allen 21 Tagen

An wie vielen Tagen wollen Sie in den nächsten drei Wochen Karamellriegel essen?|an keinem Tag|an allen 21 Tagen

F2. Marmelade

An wie vielen Tagen haben Sie in den letzten drei Wochen Marmelade gegessen?|an keinem Tag|an allen 21 Tagen

An wie vielen Tagen wollen Sie in den nächsten drei Wochen Marmelade essen?|an keinem Tag|an allen 21 Tagen

F3. **Frühlingsrollen**

An wie vielen Tagen haben Sie in den letzten drei Wochen Frühlingsrollen gegessen?|an keinem Tag|an allen 21 Tagen

An wie vielen Tagen wollen Sie in den nächsten drei Wochen Frühlingsrollen essen?|an keinem Tag|an allen 21 Tagen

F4. **Butter**

An wie vielen Tagen haben Sie in den letzten drei Wochen Butter gegessen?|an keinem Tag|an allen 21 Tagen

An wie vielen Tagen wollen Sie in den nächsten drei Wochen Butter essen?|an keinem Tag|an allen 21 Tagen

F5. **Mandarine/ Orange**

An wie vielen Tagen haben Sie in den letzten drei Wochen Mandarine/ Orange gegessen?|an keinem Tag|an allen 21 Tagen

An wie vielen Tagen wollen Sie in den nächsten drei Wochen Mandarine/ Orange essen?|an keinem Tag|an allen 21 Tagen

F6. **Müsliriegel**

An wie vielen Tagen haben Sie in den letzten drei Wochen Müsliriegel gegessen?|an keinem Tag|an allen 21 Tagen

An wie vielen Tagen wollen Sie in den nächsten drei Wochen Müsliriegel essen?|an keinem Tag|an allen 21 Tagen

F7. **Weingummis**

An wie vielen Tagen haben Sie in den letzten drei Wochen Weingummis gegessen?|an keinem Tag|an allen 21 Tagen

An wie vielen Tagen wollen Sie in den nächsten drei Wochen Weingummis essen?|an keinem Tag|an allen 21 Tagen

F8. Schnitzel

An wie vielen Tagen haben Sie in den letzten drei Wochen Schnitzel gegessen?|an keinem Tag|an allen 21 Tagen

An wie vielen Tagen wollen Sie in den nächsten drei Wochen Schnitzel essen?|an keinem Tag|an allen 21 Tagen

F9. Chips

An wie vielen Tagen haben Sie in den letzten drei Wochen Chips gegessen?|an keinem Tag|an allen 21 Tagen

An wie vielen Tagen wollen Sie in den nächsten drei Wochen Chips essen?|an keinem Tag|an allen 21 Tagen

F10. Gemüsesuppe

An wie vielen Tagen haben Sie in den letzten drei Wochen Gemüsesuppe gegessen?|an keinem Tag|an allen 21 Tagen

An wie vielen Tagen wollen Sie in den nächsten drei Wochen Gemüsesuppe essen?|an keinem Tag|an allen 21 Tagen

Teil G: Konsumziele 5/9

G1. Ei

An wie vielen Tagen haben Sie in den letzten drei Wochen Eier (in jeglicher Zubereitungsform) gegessen?|an keinem Tag|an allen 21 Tagen

An wie vielen Tagen wollen Sie in den nächsten drei Wochen Eier (in jeglicher Zubereitungsform) essen?|an keinem Tag|an allen 21 Tagen

For peer review only - http://bmjopen.bmj.com/site/about/guidelines.xhtml

G2. Tortellini

An wie vielen Tagen haben Sie in den letzten drei Wochen Tortellini gegessen?|an keinem Tag|an allen 21 Tagen

An wie vielen Tagen wollen Sie in den nächsten drei Wochen Tortellini essen?|an keinem Tag|an allen 21 Tagen

G3. Bratwurst

An wie vielen Tagen haben Sie in den letzten drei Wochen Bratwurst gegessen?|an keinem Tag|an allen 21 Tagen

An wie vielen Tagen wollen Sie in den nächsten drei Wochen Bratwurst essen?|an keinem Tag|an allen 21 Tagen

G4. Schokoladencornflakes

An wie vielen Tagen haben Sie in den letzten drei Wochen Schokoladencornflakes gegessen?|an keinem Tag|an allen 21 Tagen

An wie vielen Tagen wollen Sie in den nächsten drei Wochen Schokoladencornflakes essen?|an keinem Tag|an allen 21 Tagen

G5. Hähnchenfleisch

An wie vielen Tagen haben Sie in den letzten drei Wochen Hähnchenfleisch gegessen?|an keinem Tag|an allen 21 Tagen

An wie vielen Tagen wollen Sie in den nächsten drei Wochen Hähnchenfleisch essen?|an keinem Tag|an allen 21 Tagen

G6. Softdrinks

An wie vielen Tagen haben Sie in den letzten drei Wochen Softdrinks getrunken?|an keinem Tag|an allen 21 Tagen

An wie vielen Tagen wollen Sie in den nächsten drei Wochen Softdrinks trinken?|an keinem Tag|an allen 21 Tagen

G7. Popcorn

An wie vielen Tagen haben Sie in den letzten drei Wochen Popcorn gegessen?|an keinem Tag|an allen 21 Tagen

An wie vielen Tagen wollen Sie in den nächsten drei Wochen Popcorn essen?|an keinem Tag|an allen 21 Tagen

G8. Pfannkuchen/ Palatschinken

An wie vielen Tagen haben Sie in den letzten drei Wochen Pfannkuchen/ Palatschinken gegessen?|an keinem Tag|an allen 21 Tagen

An wie vielen Tagen wollen Sie in den nächsten drei Wochen Pfannkuchen/ Palatschinken essen?|an keinem Tag|an allen 21 Tagen

G9. Banane

An wie vielen Tagen haben Sie in den letzten drei Wochen Banane gegessen?|an keinem Tag|an allen 21 Tagen

An wie vielen Tagen wollen Sie in den nächsten drei Wochen Banane essen?|an keinem Tag|an allen 21 Tagen

G10. Käse

An wie vielen Tagen haben Sie in den letzten drei Wochen Käse gegessen?|an keinem Tag|an allen 21 Tagen

An wie vielen Tagen wollen Sie in den nächsten drei Wochen Käse essen?|an keinem Tag|an allen 21 Tagen

Teil H: Konsumziele 6/9

H1. Knödel

An wie vielen Tagen haben Sie in den letzten drei Wochen Knödel gegessen?|an keinem Tag|an allen 21 Tagen

An wie vielen Tagen wollen Sie in den nächsten drei Wochen Knödel essen?|an keinem Tag|an allen 21 Tagen

H2. Pralinen

An wie vielen Tagen haben Sie in den letzten drei Wochen Pralinen gegessen?|an keinem Tag|an allen 21 Tagen

An wie vielen Tagen wollen Sie in den nächsten drei Wochen Pralinen essen?|an keinem Tag|an allen 21 Tagen

H3. Burger

An wie vielen Tagen haben Sie in den letzten drei Wochen Burger gegessen?|an keinem Tag|an allen 21 Tagen

An wie vielen Tagen wollen Sie in den nächsten drei Wochen Burger essen?|an keinem Tag|an allen 21 Tagen

H4. Weintrauben

An wie vielen Tagen haben Sie in den letzten drei Wochen Weintrauben gegessen?|an keinem Tag|an allen 21 Tagen

An wie vielen Tagen wollen Sie in den nächsten drei Wochen Weintrauben essen?|an keinem Tag|an allen 21 Tagen

H5. Gnocchi

An wie vielen Tagen haben Sie in den letzten drei Wochen Gnocchi gegessen?|an keinem Tag|an allen 21 Tagen

An wie vielen Tagen wollen Sie in den nächsten drei Wochen Gnocchi essen?|an keinem Tag|an allen 21 Tagen

H6. Pommes

An wie vielen Tagen haben Sie in den letzten drei Wochen Pommes gegessen?|an keinem Tag|an allen 21 Tagen

An wie vielen Tagen wollen Sie in den nächsten drei Wochen Pommes essen?|an keinem Tag|an allen 21 Tagen

H7. Waffeln

An wie vielen Tagen haben Sie in den letzten drei Wochen Waffeln gegessen?|an keinem Tag|an allen 21 Tagen

An wie vielen Tagen wollen Sie in den nächsten drei Wochen Waffeln essen?|an keinem Tag|an allen 21 Tagen

H8. Käsespätzle

An wie vielen Tagen haben Sie in den letzten drei Wochen Käsespätzle gegessen?|an keinem Tag|an allen 21 Tagen

An wie vielen Tagen wollen Sie in den nächsten drei Wochen Käsespätzle essen?|an keinem Tag|an allen 21 Tagen

H9. Speiseeis

An wie vielen Tagen haben Sie in den letzten drei Wochen Speiseeis gegessen?|an keinem Tag|an allen 21 Tagen

An wie vielen Tagen wollen Sie in den nächsten drei Wochen Speiseeis essen?|an keinem Tag|an allen 21 Tagen

H10. Sushi

An wie vielen Tagen haben Sie in den letzten drei Wochen Sushi gegessen?|an keinem Tag|an allen 21 Tagen

An wie vielen Tagen wollen Sie in den nächsten drei Wochen Sushi essen?|an keinem Tag|an allen 21 Tagen

Teil I: Konsumziele 7/9

I1. Äpfel

An wie vielen Tagen haben Sie in den letzten drei Wochen Äpfel gegessen?|an keinem Tag|an allen 21 Tagen

An wie vielen Tagen wollen Sie in den nächsten drei Wochen Äpfel essen?|an keinem Tag|an allen 21 Tagen

I2. Salat

An wie vielen Tagen haben Sie in den letzten drei Wochen Salat gegessen?|an keinem Tag|an allen 21 Tagen

An wie vielen Tagen wollen Sie in den nächsten drei Wochen Salat essen?|an keinem Tag|an allen 21 Tagen

I3. Fruchtsaft

An wie vielen Tagen haben Sie in den letzten drei Wochen Fruchtsaft getrunken?|an keinem Tag|an allen 21 Tagen

An wie vielen Tagen wollen Sie in den nächsten drei Wochen Fruchtsaft trinken?|an keinem Tag|an allen 21 Tagen

I4. Kartoffeln

An wie vielen Tagen haben Sie in den letzten drei Wochen Kartoffeln gegessen?|an keinem Tag|an allen 21 Tagen

An wie vielen Tagen wollen Sie in den nächsten drei Wochen Kartoffeln essen?|an keinem Tag|an allen 21 Tagen

I5. Leberkäse

An wie vielen Tagen haben Sie in den letzten drei Wochen Leberkäse gegessen?|an keinem Tag|an allen 21 Tagen

An wie vielen Tagen wollen Sie in den nächsten drei Wochen Leberkäse essen?|an keinem Tag|an allen 21 Tagen

I6. Nachos

An wie vielen Tagen haben Sie in den letzten drei Wochen Nachos gegessen?|an keinem Tag|an allen 21 Tagen

An wie vielen Tagen wollen Sie in den nächsten drei Wochen Nachos essen?|an keinem Tag|an allen 21 Tagen

I7. Vollmilchschokolade

An wie vielen Tagen haben Sie in den letzten drei Wochen Vollmilchschokolade gegessen?|an keinem Tag|an allen 21 Tagen

An wie vielen Tagen wollen Sie in den nächsten drei Wochen Vollmilchschokolade essen?|an keinem Tag|an allen 21 Tagen

I8. Nudeln

An wie vielen Tagen haben Sie in den letzten drei Wochen Nudeln gegessen?|an keinem Tag|an allen 21 Tagen

An wie vielen Tagen wollen Sie in den nächsten drei Wochen Nudeln essen?lan keinem Taglan allen 21 Tagen

I9. Nougatcreme

An wie vielen Tagen haben Sie in den letzten drei Wochen Nougatcreme gegessen?lan keinem Taglan allen 21 Tagen

An wie vielen Tagen wollen Sie in den nächsten drei Wochen Nougatcreme essen?lan keinem Taglan allen 21 Tagen

I10. Wraps

An wie vielen Tagen haben Sie in den letzten drei Wochen Wraps gegessen?lan keinem Taglan allen 21 Tagen

An wie vielen Tagen wollen Sie in den nächsten drei Wochen Wraps essen?lan keinem Taglan allen 21 Tagen

Teil J: Konsumziele 8/9

J1. Kekse

An wie vielen Tagen haben Sie in den letzten drei Wochen Kekse gegessen?lan keinem Taglan allen 21 Tagen

An wie vielen Tagen wollen Sie in den nächsten drei Wochen Kekse essen?lan keinem Taglan allen 21 Tagen

J2. Beeren

An wie vielen Tagen haben Sie in den letzten drei Wochen Beeren gegessen?lan keinem Taglan allen 21 Tagen

An wie vielen Tagen wollen Sie in den nächsten drei Wochen Beeren essen?lan keinem Taglan allen 21 Tagen

J3. **Ofenbaguette**

An wie vielen Tagen haben Sie in den letzten drei Wochen Ofenbaguette gegessen?|an keinem Tag|an allen 21 Tagen

An wie vielen Tagen wollen Sie in den nächsten drei Wochen Ofenbaguette essen?|an keinem Tag|an allen 21 Tagen

J4. **Muffin**

An wie vielen Tagen haben Sie in den letzten drei Wochen Muffins gegessen?|an keinem Tag|an allen 21 Tagen

An wie vielen Tagen wollen Sie in den nächsten drei Wochen Muffins essen?|an keinem Tag|an allen 21 Tagen

J5. **süßes Gebäck**

An wie vielen Tagen haben Sie in den letzten drei Wochen süßes Gebäck gegessen?|an keinem Tag|an allen 21 Tagen

An wie vielen Tagen wollen Sie in den nächsten drei Wochen süßes Gebäck essen?|an keinem Tag|an allen 21 Tagen

J6. **Wurst**

An wie vielen Tagen haben Sie in den letzten drei Wochen Wurst gegessen?|an keinem Tag|an allen 21 Tagen

An wie vielen Tagen wollen Sie in den nächsten drei Wochen Wurst essen?|an keinem Tag|an allen 21 Tagen

J7. **Kuchen**

An wie vielen Tagen haben Sie in den letzten drei Wochen Kuchen gegessen?|an keinem Tag|an allen 21 Tagen

An wie vielen Tagen wollen Sie in den nächsten drei Wochen Kuchen essen?lan keinem Taglan allen 21 Tagen

J8. Kichererbsen

An wie vielen Tagen haben Sie in den letzten drei Wochen Kichererbsen gegessen?lan keinem Taglan allen 21 Tagen

An wie vielen Tagen wollen Sie in den nächsten drei Wochen Kichererbsen essen?lan keinem Taglan allen 21 Tagen

J9. Bitterschokolade

An wie vielen Tagen haben Sie in den letzten drei Wochen Bitterschokolade gegessen?lan keinem Taglan allen 21 Tagen

An wie vielen Tagen wollen Sie in den nächsten drei Wochen Bitterschokolade essen?lan keinem Taglan allen 21 Tagen

J10. Bratnudeln

An wie vielen Tagen haben Sie in den letzten drei Wochen Bratnudeln gegessen?lan keinem Taglan allen 21 Tagen

An wie vielen Tagen wollen Sie in den nächsten drei Wochen Bratnudeln essen?lan keinem Taglan allen 21 Tagen

Teil K: Konsumziele 9/9

K1. Baguette

An wie vielen Tagen haben Sie in den letzten drei Wochen Baguette gegessen?lan keinem Taglan allen 21 Tagen

An wie vielen Tagen wollen Sie in den nächsten drei Wochen Baguette essen?lan keinem Taglan allen 21 Tagen

K2. Döner

An wie vielen Tagen haben Sie in den letzten drei Wochen Döner gegessen?|an keinem Tag|an allen 21 Tagen

An wie vielen Tagen wollen Sie in den nächsten drei Wochen Döner essen?|an keinem Tag|an allen 21 Tagen

K3. Toast

An wie vielen Tagen haben Sie in den letzten drei Wochen Toast gegessen?|an keinem Tag|an allen 21 Tagen

An wie vielen Tagen wollen Sie in den nächsten drei Wochen Toast essen?|an keinem Tag|an allen 21 Tagen

K4. Rindfleisch

An wie vielen Tagen haben Sie in den letzten drei Wochen Rindfleisch gegessen?|an keinem Tag|an allen 21 Tagen

An wie vielen Tagen wollen Sie in den nächsten drei Wochen Rindfleisch essen?|an keinem Tag|an allen 21 Tagen

K5. Joghurt

An wie vielen Tagen haben Sie in den letzten drei Wochen Joghurt gegessen?|an keinem Tag|an allen 21 Tagen

An wie vielen Tagen wollen Sie in den nächsten drei Wochen Joghurt essen?|an keinem Tag|an allen 21 Tagen

K6. Schokoladenwaffeln

An wie vielen Tagen haben Sie in den letzten drei Wochen Schokoladenwaffeln gegessen?|an keinem Tag|an allen 21 Tagen

An wie vielen Tagen wollen Sie in den nächsten drei Wochen Schokoladenwaffeln essen?|an keinem Tag|an allen 21 Tagen

K7. Fleischpflanzerl/ Fleischfrikadellen

An wie vielen Tagen haben Sie in den letzten drei Wochen Fleischpflanzerl/ Fleischfrikadellen gegessen?|an keinem Tag|an allen 21 Tagen

An wie vielen Tagen wollen Sie in den nächsten drei Wochen Fleischpflanzerl/ Fleischfrikadellen essen?|an keinem Tag|an allen 21 Tagen

K8. Gemüselaibchen/ Gemüsefrikadellen

An wie vielen Tagen haben Sie in den letzten drei Wochen Gemüselaibchen/ Gemüsefrikadellen gegessen?|an keinem Tag|an allen 21 Tagen

An wie vielen Tagen wollen Sie in den nächsten drei Wochen Gemüselaibchen/ Gemüsefrikadellen essen?|an keinem Tag|an allen 21 Tagen

K9. Vollkornbrot

An wie vielen Tagen haben Sie in den letzten drei Wochen Vollkornbrot gegessen?|an keinem Tag|an allen 21 Tagen

An wie vielen Tagen wollen Sie in den nächsten drei Wochen Vollkornbrot essen?|an keinem Tag|an allen 21 Tagen

K10. Schokoladenriegel

An wie vielen Tagen haben Sie in den letzten drei Wochen Schokoladenriegel gegessen?|an keinem Tag|an allen 21 Tagen

An wie vielen Tagen wollen Sie in den nächsten drei Wochen Schokoladenriegel essen?|an keinem Tag|an allen 21 Tagen

Vielen Dank für Ihre Teilnahme an der ersten Befragung. Wir werden die erhobenen Daten zu Ihren Konsumzielen nun auswerten und uns bei Ihnen melden, um Sie über die weitere Studienteilnahme zu informieren und einen Termin für die Besprechung der Hauptstudienphase zu vereinbaren.

Sie können dieses Fenster nun schließen.

Fachbereich Psychologie
Centre for Cognitive Neuroscience,
Fachbereich für Psychologie

Univ.-Prof. Dr. Jens Blechert
jens.blechert@sbg.ac.at
Tel.: +43 / (0) 662 /8044 - 5163
Fax.: +43 / (0) 662 / 8044 – 5126

Hellbrunnerstraße 34
A-5020 Salzburg – Austria

TeilnehmerInneninformation und Einwilligungserklärung

zur Teilnahme an der Studie:

‚Kognitiv-Affektive Mechanismen von Essregulationstrainings ‘

Interne Bezeichnung: Cognitive-affective mechanisms of food biases trainings (AAI)

For peer review only

Liebe/r Interessent/in,

wir laden Sie ein an der oben genannten Studie teilzunehmen.

Im Folgenden finden Sie einige Informationen, die für Sie von Bedeutung sind, wenn Sie sich für eine Teilnahme interessieren.

Bitte unterschreiben Sie diese Einverständniserklärung nur

- wenn Sie Art und Ablauf der Studie verstanden haben
- wenn Sie bereit sind, einer Teilnahme zuzustimmen und
- wenn Sie sich über Ihre Rechte als Teilnehmer im Klaren sind.

Allgemeine Informationen und Ziele der Studie

Die Ernährungspsychologie beschäftigt sich mit Grundlagen und Behandlungsmöglichkeiten von ungesunden Essensentscheidungen sowie mit Ess- und Gewichtsstörungen. Da das Verlangen nach schmackhaften Nahrungsmitteln das Essverhalten im Alltag beeinflusst, ist es wichtig, dass wir ein besseres Verständnis für diese Zusammenhänge gewinnen, auch um Abhilfe verschaffen zu können. In dieser Studie geht es speziell darum, wie automatische Annäherungstendenzen zu Essensreizen optimal erfasst werden können, was die zugrundeliegenden Mechanismen sind und wie das Annäherungsverhalten reduziert werden kann.

Ablauf der Studie

Bei Ihrer Studienteilnahme werden wir Sie bitten, verschiedene Fragebögen zum psychischen Befinden auszufüllen. Des Weiteren wird Ihre Teilnahme nachfolgende Prozeduren beinhalten:

For peer review only - http://bmjopen.bmj.com/site/about/guidelines.xhtml

- *Betrachten von Bildern von Nahrungsmitteln*: Sie werden verschiedene Nahrungsmittel am Bildschirm betrachten und bewerten.
- *Reaktionszeitmessung via Smartphone*: Sie werden gebeten, mit schnellen Bewegungen des Smartphones auf Bilder von Nahrungsmitteln zu reagieren.
- *Fragebögen via Smartphone*: Zweimal pro Tag werden Sie gebeten über das Smartphone verschiedene Fragen zu Ihrem momentanen Empfinden und Ihrem vergangenen Essverhalten zu beantworten.
- *Reaktionszeittraining in einer von zwei Gruppen.* Ergänzend zu den oben beschriebenen Studienteilen werden Sie zudem per Zufallsauswahl *einer von zwei Studiengruppen zugeteilt*. Aus wissenschaftlichen Gründen ist eine zufällige Gruppenzuteilung notwendig, Sie können sich also nicht eine der Gruppen aussuchen. Beide Gruppen beinhalten die Bearbeitung von Reaktionszeitaufgaben am Smartphone über mehrere Termine hinweg, jedoch unterscheidet sich die Art der Reaktionszeitaufgabe geringfügig. Am Ende der Studie erklären wir Ihnen ausführlich die Hintergründe der Trainings.

Freiwilligkeit der Teilnahme

Ihre Teilnahme an dieser Erhebung ist freiwillig. Sie können diese jederzeit, ohne Angabe von Gründen, beenden. Die Ablehnung Ihrer Teilnahme oder ein vorzeitiges Beenden kann mündlich oder schriftlich (siehe Kontaktdaten am Ende des Dokuments) erfolgen, hat keine nachteiligen Folgen und Sie werden anteilig kompensiert. Sie können Ihre Einwilligung zur Speicherung der Daten bis zum Ende der Datenerhebung widerrufen, ohne dass Ihnen daraus Nachteile entstehen.

Ausschlusskriterien

Sie können an unserer Studie teilnehmen, wenn Sie zwischen 18 und 60 Jahren alt sind und ausreichende Deutschkenntnisse besitzen, um die Instruktionen während der Studie zu verstehen. Nicht teilnehmen können außerdem Personen, die schwanger sind, in ihrer Entscheidungsfähigkeit eingeschränkt sind oder wenn eine diagnostizierte Essstörung vorliegt.

Pflichten bei einer Teilnahme

Als Studienteilnehmer/-innen werden Sie gebeten, den Anweisungen der Studienleitung so weit wie möglich zu folgen und die Fragebögen aufrichtig zu beantworten. Sollten sich während der Studie Probleme und Schwierigkeiten ergeben, informieren Sie bitte die Studienleitung darüber.

Rechte bei einer Teilnahme

Ihre Teilnahme erfolgt freiwillig und Sie können sich jederzeit, auch ohne Angabe von Gründen, aus der Studie zurückziehen, und/ oder eine Löschung der erfassten Daten verlangen, ohne dass Ihnen daraus Nachteile irgendwelcher Art entstehen.

Nutzen bei einer Teilnahme

Ihre Teilnahme an dieser Studie dient der klinisch-psychologischen Wissenschaft. Neue Erkenntnisse sollen zum besseren Verständnis der Ursachen von Überessen und starkem Verlangen nach schmackhaftem Essen beitragen. Außerdem profitieren Sie durch die Teilnahme an dem Reaktionszeittraining bezüglich Ihres

For peer review only - http://bmjopen.bmj.com/site/about/guidelines.xhtml

selbstgesteckten Ernährungsziels. Nach Studienende erhalten Sie auf Wunsch ausführliche schriftliche Informationen über Sinn und Zweck der Studie sowie eine individualisierte Rückmeldung und Erklärung ihrer Ergebnisse. Des Weiteren erhalten Sie eine Aufwandsentschädigung.

Aufwandsentschädigung

Während wir davon ausgehen, dass Ihr Essverhalten vom Reaktionszeittraining profitieren wird, entsteht durch die Beantwortung der Fragebögen jedoch zusätzlicher zeitlicher Aufwand, für den wir sie finanziell kompensieren. Abhängig von der Vollständigkeit ihr Daten erhalten Sie sechs bis acht VP-Stunden beziehungsweise zwischen 40 und 60 Euro.

Mögliche Risiken und Unannehmlichkeiten

Die oben beschriebenen Prozeduren sind nicht gesundheitsschädlich und entsprechen wissenschaftlichen Standards. Sie können vorrübergehend negative physische und emotionale Empfindungen hervorrufen. Diese Empfindungen sowie spätere diesbezügliche Erinnerungen sind jedoch erfahrungsgemäß vorübergehender Natur. Sollten Sie unerwarteter Weise unter anhaltenden Belastungen aufgrund Ihrer Studienteilnahme leiden, so melden Sie sich umgehend bei einer der unten aufgeführten Kontaktpersonen.

Vertraulichkeit und Schutz der Daten

Bei den Daten ist zu unterscheiden zwischen personenbezogenen Daten, mit denen Sie direkt identifizierbar sind (z.B.: Name, Telefonnummer, Adresse), und pseudonymisierten (verschlüsselten) Daten, bei denen alle Informationen, die direkten Rückschluss auf Ihre Identität zulassen, durch einen Teilnahmecode ersetzt werden. Der **‚Schlüssel'** (Abgleich von Pseudonym mit personenbezogenen Daten) ist nur der Studienleitung zugänglich, wird nach dem Stand der Technik in passwort-geschützten Dokumenten geschützt und getrennt von den verschlüsselten Datensätzen aufbewahrt. Sämtliche Personen, die Zugang zu personenbezogenen Daten erhalten, unterliegen im Umgang mit den Daten dem österreichischen und europäischen Datenschutzrecht und sind dem Datengeheimnis verpflichtet.

Im Rahmen dieser Studie erfolgt die Verarbeitung von folgenden Daten:

- *Personenbezogenen Daten*: Name, E-mail, Telefonnummer. Diese Daten erleichtern uns die Studienadministration und die Sicherstellung einer hohen Datenqualität.
- *Pseudonymisierte Daten*: Alle Eingaben, die Sie über die App ‚m-Path' machen, werden dort unter einem selbstgewählten Teilnahmecode („Pseudonym") auf Servern der Universität Leuven (Belgien) gespeichert. Ihre Daten sind also innerhalb der App nicht Ihrer Person zu zuordnen. Auch die Daten in der App ‚Picture Game' werden nur über einen pseudonymisierten Teilnahmecode gespeichert und über einen Account der Studienleitung von Google firebase übertragen. Für beide Apps gilt: Eine Zuordnung zu Ihrer Person ist nur mit zusätzlichen Daten möglich, die ausschließlich Mitarbeitenden der PLUS vorliegen (‚Schlüssel', siehe oben).

For peer review only - http://bmjopen.bmj.com/site/about/guidelines.xhtml

Ihnen steht bezüglich Ihrer bei uns gespeicherten pseudonymisierten Daten grundsätzlich das Recht auf Auskunft, Richtigstellung, Löschung und Einschränkung zu. Sie können Ihre Einwilligung auch jederzeit widerrufen. Ein Widerruf hat zur Folge, dass wir Ihre Daten ab diesem Zeitpunkt zu den oben genannten Zwecken nicht mehr verarbeiten. Für einen Widerruf wenden Sie sich bitte an die unten angeführte Studienleitung. Wenn Sie glauben, dass wir gegen datenschutzrechtliche Vorschriften verstoßen, können Sie sich bei dem Datenschutzbeauftragten der Universität Salzburg (datenschutz@sbg.ac.at) oder bei einer Datenschutzbehörde beschweren. Weitere Informationen finden Sie unter www.uni-salzburg.at/impressum.

Wir weisen darauf hin, dass wissenschaftliche Publikationen geplant sind und dabei Studienergebnisse in ausschließlich anonymer Weise veröffentlicht werden.

Die personenbezogenen Daten und der zugehörige Schlüssel wird **1 Jahr nach Studienende gelöscht**. Ebenso werden alle Daten in der App zu dem Zeitpunkt gelöscht. Damit erlöschen auch ihre Möglichkeiten der Einsicht/Berichtigung/Export/Löschung dieser Daten, da wir Sie ihren Daten dann nicht mehr zuordnen können (da der ‚Schlüssel' gelöscht wurde). Wir behalten einzig ihren Namen und diese Einverständniserklärung als Dokumentation ihrer Studienteilname für **30 Jahre**.

Welche Kontaktpersonen stehen zur Verfügung?

Bei anfälligen Fragen, die während oder nach Abschluss der Studie auftreten, können Sie sich unter gesundheitspsychologie07@plus.ac.at oder der +43 677 616 767 07 melden und jederzeit an die Studienleitung wenden:

- Hannah van Alebeek, MSc., Fachbereich Psychologie, Universität Salzburg, Hellbrunnerstrasse 34, 5020 Salzburg, hannah.vanalebeek@plus.ac.at

Wir freuen uns auf die Zusammenarbeit!

Diese Studie wurde von der Ethikkommission der Universität Salzburg evaluiert.
Kontaktperson: Mag.ª Clara Gröblacher, Kapitelgasse 4, A-5020 Salzburg, Tel: +43-662-8044 2391, clara.groeblacher@sbg.ac.at

Einverständniserklärung

Ich habe die oben beschriebenen Informationen vollumfänglich gelesen und verstanden.

Meine Teilnahme erfolgt freiwillig und ich weiß, dass ich mich jederzeit, auch ohne Angabe von Gründen, von der Studie zurückziehen kann, ohne dass mir daraus Nachteile irgendwelcher Art entstehen.

Ich bin bereit, an dieser Studie teilzunehmen und bin mit der Erhebung und Verwendung persönlicher Daten nach Maßgabe der TeilnehmerInneninformation einverstanden.

Ort/Datum ______________________________

Name TeilnehmerIn ______________________________

Unterschrift TeilnehmerIn ______________________________

Unterschrift Studienverantwortliche Person ______________________________

For peer review only - http://bmjopen.bmj.com/site/about/guidelines.xhtml

– Dieses Dokument bleibt bei der Versuchsleitung –

Einverständniserklärung

Ich habe die oben beschriebenen Informationen vollumfänglich gelesen und verstanden. Außerdem nehme ich zur Kenntnis, dass ich für Wege zum/vom Untersuchungsort, nicht wege-/unfallversichert bin.

Meine Teilnahme erfolgt freiwillig und ich weiß, dass ich mich jederzeit, auch ohne Angabe von Gründen, von der Studie zurückziehen kann, ohne dass mir daraus Nachteile irgendwelcher Art entstehen.

Ich bin bereit, an dieser Studie teilzunehmen und bin mit der Erhebung und Verwendung persönlicher Daten nach Maßgabe der TeilnehmerInneninformation einverstanden. Eine Kopie dieser Studieninformation und Einverständniserklärung wurde mir ausgehändigt.

Ort/Datum ____________________________________

Name TeilnehmerIn ____________________________________

Unterschrift TeilnehmerIn ____________________________________

Unterschrift
Studienverantwortliche Person ____________________________________

For peer review only

For peer review only - http://bmjopen.bmj.com/site/about/guidelines.xhtml

Reporting checklist for protocol of a clinical trial.

Based on the SPIRIT guidelines.

Instructions to authors

Complete this checklist by entering the page numbers from your manuscript where readers will find each of the items listed below.

Your article may not currently address all the items on the checklist. Please modify your text to include the missing information. If you are certain that an item does not apply, please write "n/a" and provide a short explanation.

Upload your completed checklist as an extra file when you submit to a journal.

In your methods section, say that you used the SPIRITreporting guidelines, and cite them as:

Chan A-W, Tetzlaff JM, Gøtzsche PC, Altman DG, Mann H, Berlin J, Dickersin K, Hróbjartsson A, Schulz KF, Parulekar WR, Krleža-Jerić K, Laupacis A, Moher D. SPIRIT 2013 Explanation and Elaboration: Guidance for protocols of clinical trials. BMJ. 2013;346:e7586

		Reporting Item	Page Number
**Administrative information**			
Title	#1	Descriptive title identifying the study design, population, interventions, and, if applicable, trial acronym	1
Trial registration	#2a	Trial identifier and registry name. If not yet registered,	2

For peer review only

		name of intended registry	
Trial registration: data set	#2b	All items from the World Health Organization Trial Registration Data Set	n/a
Protocol version	#3	Date and version identifier	n/a
Funding	#4	Sources and types of financial, material, and other support	12
Roles and responsibilities: contributorship	#5a	Names, affiliations, and roles of protocol contributors	12
Roles and responsibilities: sponsor contact information	#5b	Name and contact information for the trial sponsor	1
Roles and responsibilities: sponsor and funder	#5c	Role of study sponsor and funders, if any, in study design; collection, management, analysis, and interpretation of data; writing of the report; and the decision to submit the report for publication, including whether they will have ultimate authority over any of these activities	12
Roles and responsibilities: committees	#5d	Composition, roles, and responsibilities of the coordinating centre, steering committee, endpoint adjudication committee, data management team, and other individuals or groups overseeing the trial, if applicable (see Item 21a for data monitoring committee)	n/a

Introduction

For peer review only

Background and rationale	#6a	Description of research question and justification for undertaking the trial, including summary of relevant studies (published and unpublished) examining benefits and harms for each intervention	3-5
Background and rationale: choice of comparators	#6b	Explanation for choice of comparators	3-5
Objectives	#7	Specific objectives or hypotheses	5
Trial design	#8	Description of trial design including type of trial (eg, parallel group, crossover, factorial, single group), allocation ratio, and framework (eg, superiority, equivalence, non-inferiority, exploratory)	5-7
**Methods: Participants, interventions, and outcomes**			
Study setting	#9	Description of study settings (eg, community clinic, academic hospital) and list of countries where data will be collected. Reference to where list of study sites can be obtained	5-8
Eligibility criteria	#10	Inclusion and exclusion criteria for participants. If applicable, eligibility criteria for study centres and individuals who will perform the interventions (eg,	5

For peer review only

		surgeons, psychotherapists)	
Interventions: description	#11a	Interventions for each group with sufficient detail to allow replication, including how and when they will be administered	6
Interventions: modifications	#11b	Criteria for discontinuing or modifying allocated interventions for a given trial participant (eg, drug dose change in response to harms, participant request, or improving / worsening disease)	n/a
Interventions: adherance	#11c	Strategies to improve adherence to intervention protocols, and any procedures for monitoring adherence (eg, drug tablet return; laboratory tests)	5
Interventions: concomitant care	#11d	Relevant concomitant care and interventions that are permitted or prohibited during the trial	n/a
Outcomes	#12	Primary, secondary, and other outcomes, including the specific measurement variable (eg, systolic blood pressure), analysis metric (eg, change from baseline, final value, time to event), method of aggregation (eg, median, proportion), and time point for each outcome. Explanation of the clinical relevance of chosen efficacy and harm outcomes is strongly recommended	9-10
Participant timeline	#13	Time schedule of enrolment, interventions (including any run-ins and washouts), assessments, and visits for participants. A schematic diagram is highly recommended (see Figure)	9

For peer review only

Sample size	#14	Estimated number of participants needed to achieve study objectives and how it was determined, including clinical and statistical assumptions supporting any sample size calculations	5
Recruitment	#15	Strategies for achieving adequate participant enrolment to reach target sample size	5
**Methods: Assignment of interventions (for controlled trials)**			
Allocation: sequence generation	#16a	Method of generating the allocation sequence (eg, computer-generated random numbers), and list of any factors for stratification. To reduce predictability of a random sequence, details of any planned restriction (eg, blocking) should be provided in a separate document that is unavailable to those who enrol participants or assign interventions	9
Allocation concealment mechanism	#16b	Mechanism of implementing the allocation sequence (eg, central telephone; sequentially numbered, opaque, sealed envelopes), describing any steps to conceal the sequence until interventions are assigned	9
Allocation: implementation	#16c	Who will generate the allocation sequence, who will enrol participants, and who will assign participants to interventions	9

For peer review only

Blinding (masking)	#17a	Who will be blinded after assignment to interventions (eg, trial participants, care providers, outcome assessors, data analysts), and how	9
Blinding (masking): emergency unblinding	#17b	If blinded, circumstances under which unblinding is permissible, and procedure for revealing a participant's allocated intervention during the trial	n/a
**Methods: Data collection, management, and analysis**			
Data collection plan	#18a	Plans for assessment and collection of outcome, baseline, and other trial data, including any related processes to promote data quality (eg, duplicate measurements, training of assessors) and a description of study instruments (eg, questionnaires, laboratory tests) along with their reliability and validity, if known. Reference to where data collection forms can be found, if not in the protocol	6-10
Data collection plan: retention	#18b	Plans to promote participant retention and complete follow-up, including list of any outcome data to be collected for participants who discontinue or deviate from intervention protocols	6-10
Data management	#19	Plans for data entry, coding, security, and storage, including any related processes to promote data quality (eg, double data entry; range checks for data values).	10

For peer review only

		Reference to where details of data management procedures can be found, if not in the protocol	
Statistics: outcomes	#20a	Statistical methods for analysing primary and secondary outcomes. Reference to where other details of the statistical analysis plan can be found, if not in the protocol	9-10
Statistics: additional analyses	#20b	Methods for any additional analyses (eg, subgroup and adjusted analyses)	9-10
Statistics: analysis population and missing data	#20c	Definition of analysis population relating to protocol non-adherence (eg, as randomised analysis), and any statistical methods to handle missing data (eg, multiple imputation)	9-10
**Methods: Monitoring**			
Data monitoring: formal committee	#21a	Composition of data monitoring committee (DMC); summary of its role and reporting structure; statement of whether it is independent from the sponsor and competing interests; and reference to where further details about its charter can be found, if not in the protocol. Alternatively, an explanation of why a DMC is not needed	n/a
Data monitoring: interim analysis	#21b	Description of any interim analyses and stopping guidelines, including who will have access to these interim results and make the final decision to terminate the trial	n/a
Harms	#22	Plans for collecting, assessing, reporting, and managing solicited and spontaneously reported adverse events and other unintended effects of trial interventions or trial	n/a

For peer review only

		conduct	
Auditing	#23	Frequency and procedures for auditing trial conduct, if any, and whether the process will be independent from investigators and the sponsor	n/a
**Ethics and dissemination**			
Research ethics approval	#24	Plans for seeking research ethics committee / institutional review board (REC / IRB) approval	2
Protocol amendments	#25	Plans for communicating important protocol modifications (eg, changes to eligibility criteria, outcomes, analyses) to relevant parties (eg, investigators, REC / IRBs, trial participants, trial registries, journals, regulators)	n/a
Consent or assent	#26a	Who will obtain informed consent or assent from potential trial participants or authorised surrogates, and how (see Item 32)	6
Consent or assent: ancillary studies	#26b	Additional consent provisions for collection and use of participant data and biological specimens in ancillary studies, if applicable	n/a
Confidentiality	#27	How personal information about potential and enrolled participants will be collected, shared, and maintained in order to protect confidentiality before, during, and after the trial	10
Declaration of	#28	Financial and other competing interests for principal	12

For peer review only

interests		investigators for the overall trial and each study site	
Data access	#29	Statement of who will have access to the final trial dataset, and disclosure of contractual agreements that limit such access for investigators	11
Ancillary and post trial care	#30	Provisions, if any, for ancillary and post-trial care, and for compensation to those who suffer harm from trial participation	n/a
Dissemination policy: trial results	#31a	Plans for investigators and sponsor to communicate trial results to participants, healthcare professionals, the public, and other relevant groups (eg, via publication, reporting in results databases, or other data sharing arrangements), including any publication restrictions	11
Dissemination policy: authorship	#31b	Authorship eligibility guidelines and any intended use of professional writers	n/a
Dissemination policy: reproducible research	#31c	Plans, if any, for granting public access to the full protocol, participant-level dataset, and statistical code	11
**Appendices**			
Informed consent materials	#32	Model consent form and other related documentation given to participants and authorised surrogates	n/a
Biological specimens	#33	Plans for collection, laboratory evaluation, and storage of biological specimens for genetic or molecular analysis in the current trial and for future use in ancillary studies, if applicable	n/a

For peer review only

The SPIRIT Explanation and Elaboration paper is distributed under the terms of the Creative Commons Attribution License CC-BY-NC. This checklist was completed on 23. November 2022 using https://www.goodreports.org/, a tool made by the EQUATOR Network in collaboration with Penelope.ai

For peer review only
